# Effect of neural connectivity on autocovariance and cross covariance estimates

**DOI:** 10.1186/1475-925X-6-3

**Published:** 2007-01-16

**Authors:** Mark M Stecker

**Affiliations:** 1 Department of Neurology, Geisinger Medical Center, Danville, PA 17821 USA

## Abstract

**Background:**

Measurements of auto and cross covariance functions are frequently used to investigate neural systems. In interpreting this data, it is commonly assumed that the largest contribution to the recordings comes from sources near the electrode. However, the potential recorded at an electrode represents the superimposition of the potentials generated by large numbers of active neural structures. This creates situations under which the measured auto and cross covariance functions are dominated by the activity in structures far from the electrode and in which the distance dependence of the cross-covariance function differs significantly from that describing the activity in the actual neural structures.

**Methods:**

Direct application of electrostatics to calculate the theoretical auto and cross covariance functions that would be recorded from electrodes immersed in a large volume filled with active neural structures with specific statistical properties.

**Results:**

It is demonstrated that the potentials recorded from a monopolar electrode surrounded by dipole sources in a uniform medium are predominantly due to activity in neural structures far from the electrode when neuronal correlations drop more slowly than 1/r^3 ^or when the size of the neural system is much smaller than a known correlation distance. Recordings from quadrupolar sources are strongly dependent on distant neurons when correlations drop more slowly than 1/r or the size of the system is much smaller than the correlation distance. Differences between bipolar and monopolar recordings are discussed. It is also demonstrated that the cross covariance of the recorded in two spatially separated electrodes declines as a power-law function of the distance between them even when the electrical activity from different neuronal structures is uncorrelated.

**Conclusion:**

When extracellular electrophysiologic recordings are made from systems containing large numbers of neural structures, it is important to interpret measured auto and cross covariance functions cautiously in light of the long range nature of the electric fields. Using recording electrodes that are bipolar or quadrupolar minimizes or eliminates these effects and hence these electrodes are preferred when electrical recordings are made for the purpose of auto and cross correlation analysis of local electrical activity.

## 1. Background

Recordings of spontaneous or evoked electrical activity from large groups of neurons are often used to probe the function of neural systems. One fundamental problem that arises in interpreting such data is finding the location the generators of specific electrical patterns from recordings made at a distance from the sources. This "inverse problem" does not have a unique solution [1,2] since there are localized charge distributions such as the closed dipole sheet that do not produce electric fields outside the region containing the sources [3] and hence do not change the measured potential. However, if additional restrictions can be placed on the types of source distributions that are realistic, a unique solution may be found [4] in certain cases. The most common set of simplifying assumptions is that the source consists of a single or very few dipoles but this assumption may not be reasonable if there are strong correlations between distant neurons. Because of the difficulties inherent in solving the "inverse problem", it is very important to obtain insight into this problem through an understanding of the "forward problem" of predicting the electric fields generated by various known generators.

The primary goal of this paper is to consider the "forward problem" of computing the fields generated by large numbers of correlated neural structures with the specific intent of understanding those features of the generator, recording electrode, and neuronal correlation function which determine whether recordings of electrical activity are dominated by neurons near the electrode or neurons far from the electrode. From the outset, it should be noted that this problem is very different from that of determining the distribution of fields generated by individual sources. Even in that simple problem there are "near field potentials" such as the quadrupolar fields generated by travelling action potentials [5] which are highly peaked near the source and "far field potentials" which can vary more slowly than 1/r at large distances from the source [5]. In this paper, only sources associated with traditional "near field potentials" will be considered. The secondary goal will be to demonstrate the effect that long range nature of electrostatic fields has on the cross covariance between the signals recorded from electrodes separated by a given distance.

Specifically, in this paper, the auto and cross covariance functions for signals recorded from electrodes immersed in a large continuous medium of neural elements will be considered. Since the goals of this paper are qualitative rather than quantitative, a very simplistic model system will be studied in which a group of very small recording electrodes is placed at the center of a very large homogeneous spherical region containing identical neural structures. The dependence of the field recorded from these electrodes on the radius of the spherical region will be taken as an indicator as to whether there is a significant contribution to the recorded potential from neurons far from the electrode. The measured cross covariance function will be compared with the actual cross covariance of activity in spatially separate neural structures.

## 2. Methods/Results

### 2.1 The paradox

Consider the situation in which a small electrode of radius a is placed in a homogeneous spherical region of radius R filled with uniformly distributed neural generators. One goal of this paper will be to compute the cross covariance function: *ν*_*aR *_(x→
 MathType@MTEF@5@5@+=feaafiart1ev1aaatCvAUfKttLearuWrP9MDH5MBPbIqV92AaeXatLxBI9gBaebbnrfifHhDYfgasaacH8akY=wiFfYdH8Gipec8Eeeu0xXdbba9frFj0=OqFfea0dXdd9vqai=hGuQ8kuc9pgc9s8qqaq=dirpe0xb9q8qiLsFr0=vr0=vr0dc8meaabaqaciaacaGaaeqabaqabeGadaaakeaacuWG4baEgaWcaaaa@2E37@, s→
 MathType@MTEF@5@5@+=feaafiart1ev1aaatCvAUfKttLearuWrP9MDH5MBPbIqV92AaeXatLxBI9gBaebbnrfifHhDYfgasaacH8akY=wiFfYdH8Gipec8Eeeu0xXdbba9frFj0=OqFfea0dXdd9vqai=hGuQ8kuc9pgc9s8qqaq=dirpe0xb9q8qiLsFr0=vr0=vr0dc8meaabaqaciaacaGaaeqabaqabeGadaaakeaacuWGZbWCgaWcaaaa@2E2D@, *τ*) which represents the cross covariance between signals recorded from two electrodes one located at position x→
 MathType@MTEF@5@5@+=feaafiart1ev1aaatCvAUfKttLearuWrP9MDH5MBPbIqV92AaeXatLxBI9gBaebbnrfifHhDYfgasaacH8akY=wiFfYdH8Gipec8Eeeu0xXdbba9frFj0=OqFfea0dXdd9vqai=hGuQ8kuc9pgc9s8qqaq=dirpe0xb9q8qiLsFr0=vr0=vr0dc8meaabaqaciaacaGaaeqabaqabeGadaaakeaacuWG4baEgaWcaaaa@2E37@ and another located a distance s→
 MathType@MTEF@5@5@+=feaafiart1ev1aaatCvAUfKttLearuWrP9MDH5MBPbIqV92AaeXatLxBI9gBaebbnrfifHhDYfgasaacH8akY=wiFfYdH8Gipec8Eeeu0xXdbba9frFj0=OqFfea0dXdd9vqai=hGuQ8kuc9pgc9s8qqaq=dirpe0xb9q8qiLsFr0=vr0=vr0dc8meaabaqaciaacaGaaeqabaqabeGadaaakeaacuWGZbWCgaWcaaaa@2E2D@ away from the first as a function of a, R, *τ*(time difference) and the assumptions about the correlation between neural generators throughout the medium. Also of interest will be a study of *ν*_*aR *_(*τ*) = *ν*_*aR *_(0,0, *τ*) which is the autocovariance function for signals recorded from the center of the spherical medium as a function of R. Situations in which the value of lim⁡R→∞νaR(τ)
 MathType@MTEF@5@5@+=feaafiart1ev1aaatCvAUfKttLearuWrP9MDH5MBPbIqV92AaeXatLxBI9gBaebbnrfifHhDYfgasaacH8akY=wiFfYdH8Gipec8Eeeu0xXdbba9frFj0=OqFfea0dXdd9vqai=hGuQ8kuc9pgc9s8qqaq=dirpe0xb9q8qiLsFr0=vr0=vr0dc8meaabaqaciaacaGaaeqabaqabeGadaaakeaadaWfqaqaaiGbcYgaSjabcMgaPjabc2gaTbWcbaGaemOuaiLaeyOKH4QaeyOhIukabeaaiiGakiab=17aUnaaBaaaleaacqWGHbqycqWGsbGuaeqaaOGaeiikaGIae8hXdqNaeiykaKcaaa@3D77@ does not exist suggest that under these conditions, recordings will be dominated by contributions from distant neurons and will be strongly dependent on the size and shape of the region in which the electrode is immersed. The core of the problem can be easily illustrated in a simple argument reminiscent of that which leads to Olber's paradox. If the potential from a set of neural sources a distance r from an electrode falls as 1rm
 MathType@MTEF@5@5@+=feaafiart1ev1aaatCvAUfKttLearuWrP9MDH5MBPbIqV92AaeXatLxBI9gBaebbnrfifHhDYfgasaacH8akY=wiFfYdH8Gipec8Eeeu0xXdbba9frFj0=OqFfea0dXdd9vqai=hGuQ8kuc9pgc9s8qqaq=dirpe0xb9q8qiLsFr0=vr0=vr0dc8meaabaqaciaacaGaaeqabaqabeGadaaakeaadaWcaaqaaiabigdaXaqaaiabdkhaYnaaCaaaleqabaGaemyBa0gaaaaaaaa@30A9@ the net contribution to the potential from all neural sources in a spherical shell located at distances between R and *R *+ Δ*R *from the electrode will be proportional to 4πR2ΔR1Rm
 MathType@MTEF@5@5@+=feaafiart1ev1aaatCvAUfKttLearuWrP9MDH5MBPbIqV92AaeXatLxBI9gBaebbnrfifHhDYfgasaacH8akY=wiFfYdH8Gipec8Eeeu0xXdbba9frFj0=OqFfea0dXdd9vqai=hGuQ8kuc9pgc9s8qqaq=dirpe0xb9q8qiLsFr0=vr0=vr0dc8meaabaqaciaacaGaaeqabaqabeGadaaakeaacqaI0aaniiGacqWFapaCcqWGsbGudaahaaWcbeqaaiabikdaYaaakiabgs5aejabdkfasnaalaaabaGaeGymaedabaGaemOuai1aaWbaaSqabeaacqWGTbqBaaaaaaaa@380D@. If m is less than or equal to 2 each successively more distant shell of sources contributes either a greater or equal amount to the total potential and hence the recorded potential is very sensitive to the exact shape and size of the neural region. When m = 3, the contribution from each successively more distant shell diminishes as 1R
 MathType@MTEF@5@5@+=feaafiart1ev1aaatCvAUfKttLearuWrP9MDH5MBPbIqV92AaeXatLxBI9gBaebbnrfifHhDYfgasaacH8akY=wiFfYdH8Gipec8Eeeu0xXdbba9frFj0=OqFfea0dXdd9vqai=hGuQ8kuc9pgc9s8qqaq=dirpe0xb9q8qiLsFr0=vr0=vr0dc8meaabaqaciaacaGaaeqabaqabeGadaaakeaadaWcaaqaaiabigdaXaqaaiabdkfasbaaaaa@2ED9@ but the total potential diverges logarithmically and so, even in this case, it is expected that the recorded potential will be strongly dependent on the shape and size of the volume in which the recording electrode is placed. Only when m > 3 does the measured potential reach a finite limit for large values of R and hence most of the electrical activity recorded can be considered to come from neurons near the electrode. This result suggests that when recording from highly correlated dipolar or quadrupolar sources most of the electrical activity recorded from an electrode comes from neurons far from the electrode. This paradoxical result arises out of the implicit assumption that neuronal activity is highly correlated even at large distances. The following discussion will demonstrate how the above result depends on the details of the correlation function describing activity in spatially separated neurons.

### 2.2 The multipole expansion

The general expression for the electric potential produced at the location of the electrode x→
 MathType@MTEF@5@5@+=feaafiart1ev1aaatCvAUfKttLearuWrP9MDH5MBPbIqV92AaeXatLxBI9gBaebbnrfifHhDYfgasaacH8akY=wiFfYdH8Gipec8Eeeu0xXdbba9frFj0=OqFfea0dXdd9vqai=hGuQ8kuc9pgc9s8qqaq=dirpe0xb9q8qiLsFr0=vr0=vr0dc8meaabaqaciaacaGaaeqabaqabeGadaaakeaacuWG4baEgaWcaaaa@2E37@ is:

ϕ(x→,t)=∫Vd3x→′ρ(x→′,t)|x→−x→′|     (1)
 MathType@MTEF@5@5@+=feaafiart1ev1aaatCvAUfKttLearuWrP9MDH5MBPbIqV92AaeXatLxBI9gBaebbnrfifHhDYfgasaacH8akY=wiFfYdH8Gipec8Eeeu0xXdbba9frFj0=OqFfea0dXdd9vqai=hGuQ8kuc9pgc9s8qqaq=dirpe0xb9q8qiLsFr0=vr0=vr0dc8meaabaqaciaacaGaaeqabaqabeGadaaakeaaiiGacqWFvpGAcqGGOaakcuWG4baEgaWcaiabcYcaSiabdsha0jabcMcaPiabg2da9maapefabaGaemizaq2aaWbaaSqabeaacqaIZaWmaaGccuWG4baEgaWcgaqbaaWcbaGaemOvayfabeqdcqGHRiI8aOWaaSaaaeaacqWFbpGCcqGGOaakcuWG4baEgaWcgaqbaiabcYcaSiabdsha0jabcMcaPaqaaiabcYha8jqbdIha4zaalaGaeyOeI0IafmiEaGNbaSGbauaacqGG8baFaaGaaCzcaiaaxMaadaqadaqaaiabigdaXaGaayjkaiaawMcaaaaa@4EDD@

where V is the spherical volume of radius R and *ρ*(x→
 MathType@MTEF@5@5@+=feaafiart1ev1aaatCvAUfKttLearuWrP9MDH5MBPbIqV92AaeXatLxBI9gBaebbnrfifHhDYfgasaacH8akY=wiFfYdH8Gipec8Eeeu0xXdbba9frFj0=OqFfea0dXdd9vqai=hGuQ8kuc9pgc9s8qqaq=dirpe0xb9q8qiLsFr0=vr0=vr0dc8meaabaqaciaacaGaaeqabaqabeGadaaakeaacuWG4baEgaWcaaaa@2E37@', *t*) is the charge density at the point x→
 MathType@MTEF@5@5@+=feaafiart1ev1aaatCvAUfKttLearuWrP9MDH5MBPbIqV92AaeXatLxBI9gBaebbnrfifHhDYfgasaacH8akY=wiFfYdH8Gipec8Eeeu0xXdbba9frFj0=OqFfea0dXdd9vqai=hGuQ8kuc9pgc9s8qqaq=dirpe0xb9q8qiLsFr0=vr0=vr0dc8meaabaqaciaacaGaaeqabaqabeGadaaakeaacuWG4baEgaWcaaaa@2E37@' and time t. Consider the contribution to the total potential made by the generators in a small element of space ΔVx→1
 MathType@MTEF@5@5@+=feaafiart1ev1aaatCvAUfKttLearuWrP9MDH5MBPbIqV92AaeXatLxBI9gBaebbnrfifHhDYfgasaacH8akY=wiFfYdH8Gipec8Eeeu0xXdbba9frFj0=OqFfea0dXdd9vqai=hGuQ8kuc9pgc9s8qqaq=dirpe0xb9q8qiLsFr0=vr0=vr0dc8meaabaqaciaacaGaaeqabaqabeGadaaakeaacqGHuoarcqWGwbGvdaWgaaWcbaGafmiEaGNbaSaadaWgaaadbaGaeGymaedabeaaaSqabaaaaa@3227@ centered around the point x→
 MathType@MTEF@5@5@+=feaafiart1ev1aaatCvAUfKttLearuWrP9MDH5MBPbIqV92AaeXatLxBI9gBaebbnrfifHhDYfgasaacH8akY=wiFfYdH8Gipec8Eeeu0xXdbba9frFj0=OqFfea0dXdd9vqai=hGuQ8kuc9pgc9s8qqaq=dirpe0xb9q8qiLsFr0=vr0=vr0dc8meaabaqaciaacaGaaeqabaqabeGadaaakeaacuWG4baEgaWcaaaa@2E37@_1_. This is given by:

ϕ(x→,t,ΔVx→1)=∫ΔVx→1d3x→″ρ(x→1+x→″,t)|x→−x→1−x→″|     (2)
 MathType@MTEF@5@5@+=feaafiart1ev1aaatCvAUfKttLearuWrP9MDH5MBPbIqV92AaeXatLxBI9gBaebbnrfifHhDYfgasaacH8akY=wiFfYdH8Gipec8Eeeu0xXdbba9frFj0=OqFfea0dXdd9vqai=hGuQ8kuc9pgc9s8qqaq=dirpe0xb9q8qiLsFr0=vr0=vr0dc8meaabaqaciaacaGaaeqabaqabeGadaaakeaaiiGacqWFvpGAcqGGOaakcuWG4baEgaWcaiabcYcaSiabdsha0jabcYcaSiabgs5aejabdAfawnaaBaaaleaacuWG4baEgaWcamaaBaaameaacqaIXaqmaeqaaaWcbeaakiabcMcaPiabg2da9maapefabaGaemizaq2aaWbaaSqabeaacqaIZaWmaaGccuWG4baEgaWcgaGbaaWcbaGaeyiLdqKaemOvay1aaSbaaWqaaiqbdIha4zaalaWaaSbaaeaacqaIXaqmaeqaaaqabaaaleqaniabgUIiYdGcdaWcaaqaaiab=f8aYjabcIcaOiqbdIha4zaalaWaaSbaaSqaaiabigdaXaqabaGccqGHRaWkcuWG4baEgaWcgaGbaiabcYcaSiabdsha0jabcMcaPaqaaiabcYha8jqbdIha4zaalaGaeyOeI0IafmiEaGNbaSaadaWgaaWcbaGaeGymaedabeaakiabgkHiTiqbdIha4zaalyaagaGaeiiFaWhaaiaaxMaacaWLjaWaaeWaaeaacqaIYaGmaiaawIcacaGLPaaaaaa@60B3@

This expression can be rearranged to display the potential in terms of the multipole expansion of the charge density in the region by using the relation [3]:

1|x→−x→1−x→″|=4π∑l=0∞∑m=−ll12l+1|x→″|l|x→−x→1|l+1Ylm∗(θ″,φ″)Ylm(θ1,φ1)     (3)
 MathType@MTEF@5@5@+=feaafiart1ev1aaatCvAUfKttLearuWrP9MDH5MBPbIqV92AaeXatLxBI9gBaebbnrfifHhDYfgasaacH8akY=wiFfYdH8Gipec8Eeeu0xXdbba9frFj0=OqFfea0dXdd9vqai=hGuQ8kuc9pgc9s8qqaq=dirpe0xb9q8qiLsFr0=vr0=vr0dc8meaabaqaciaacaGaaeqabaqabeGadaaakeaadaWcaaqaaiabigdaXaqaaiabcYha8jqbdIha4zaalaGaeyOeI0IafmiEaGNbaSaadaWgaaWcbaGaeGymaedabeaakiabgkHiTiqbdIha4zaalyaagaGaeiiFaWhaaiabg2da9iabisda0GGaciab=b8aWnaaqahabaWaaabCaeaadaWcaaqaaiabigdaXaqaaiabikdaYiabdYgaSjabgUcaRiabigdaXaaadaWcaaqaaiabcYha8jqbdIha4zaalyaagaGaeiiFaW3aaWbaaSqabeaacqWGSbaBaaaakeaacqGG8baFcuWG4baEgaWcaiabgkHiTiqbdIha4zaalaWaaSbaaSqaaiabigdaXaqabaGccqGG8baFdaahaaWcbeqaaiabdYgaSjabgUcaRiabigdaXaaaaaaabaGaemyBa0Maeyypa0JaeyOeI0IaemiBaWgabaGaemiBaWganiabggHiLdaaleaacqWGSbaBcqGH9aqpcqaIWaamaeaacqGHEisPa0GaeyyeIuoakiabdMfaznaaDaaaleaacqWGSbaBcqWGTbqBaeaacqGHxiIkaaGccqGGOaakcuWF4oqCgaGbaiabcYcaSiqb=z8aMzaagaGaeiykaKIaemywaK1aaSbaaSqaaiabdYgaSjabd2gaTbqabaGccqGGOaakcqWF4oqCdaWgaaWcbaGaeGymaedabeaakiabcYcaSiab=z8aMnaaBaaaleaacqaIXaqmaeqaaOGaeiykaKIaaCzcaiaaxMaadaqadaqaaiabiodaZaGaayjkaiaawMcaaaaa@7DD6@

where the angles (*θ*", *φ*") describe local coordinates (i.e. orientation of x→
 MathType@MTEF@5@5@+=feaafiart1ev1aaatCvAUfKttLearuWrP9MDH5MBPbIqV92AaeXatLxBI9gBaebbnrfifHhDYfgasaacH8akY=wiFfYdH8Gipec8Eeeu0xXdbba9frFj0=OqFfea0dXdd9vqai=hGuQ8kuc9pgc9s8qqaq=dirpe0xb9q8qiLsFr0=vr0=vr0dc8meaabaqaciaacaGaaeqabaqabeGadaaakeaacuWG4baEgaWcaaaa@2E37@") within ΔVx→1
 MathType@MTEF@5@5@+=feaafiart1ev1aaatCvAUfKttLearuWrP9MDH5MBPbIqV92AaeXatLxBI9gBaebbnrfifHhDYfgasaacH8akY=wiFfYdH8Gipec8Eeeu0xXdbba9frFj0=OqFfea0dXdd9vqai=hGuQ8kuc9pgc9s8qqaq=dirpe0xb9q8qiLsFr0=vr0=vr0dc8meaabaqaciaacaGaaeqabaqabeGadaaakeaacqGHuoarcqWGwbGvdaWgaaWcbaGafmiEaGNbaSaadaWgaaadbaGaeGymaedabeaaaSqabaaaaa@3227@, (*θ*_1_, *φ*_1_") describe the orientation of the region ΔVx→1
 MathType@MTEF@5@5@+=feaafiart1ev1aaatCvAUfKttLearuWrP9MDH5MBPbIqV92AaeXatLxBI9gBaebbnrfifHhDYfgasaacH8akY=wiFfYdH8Gipec8Eeeu0xXdbba9frFj0=OqFfea0dXdd9vqai=hGuQ8kuc9pgc9s8qqaq=dirpe0xb9q8qiLsFr0=vr0=vr0dc8meaabaqaciaacaGaaeqabaqabeGadaaakeaacqGHuoarcqWGwbGvdaWgaaWcbaGafmiEaGNbaSaadaWgaaadbaGaeGymaedabeaaaSqabaaaaa@3227@ in relation to the origin of coordinates, and it is assumed that the region of interest is smaller in its maximum diameter than the distance between the origin of coordinates and the region. The *Y*_*lm *_(*θ*, *φ*) are spherical harmonics and Ylm∗
 MathType@MTEF@5@5@+=feaafiart1ev1aaatCvAUfKttLearuWrP9MDH5MBPbIqV92AaeXatLxBI9gBaebbnrfifHhDYfgasaacH8akY=wiFfYdH8Gipec8Eeeu0xXdbba9frFj0=OqFfea0dXdd9vqai=hGuQ8kuc9pgc9s8qqaq=dirpe0xb9q8qiLsFr0=vr0=vr0dc8meaabaqaciaacaGaaeqabaqabeGadaaakeaacqWGzbqwdaqhaaWcbaGaemiBaWMaemyBa0gabaGaey4fIOcaaaaa@31C7@ (*θ*, *φ*) are their complex conjugates. Substituting (3) into (2) demonstrates that the total contribution to the potential from sources in the region ΔVx→1
 MathType@MTEF@5@5@+=feaafiart1ev1aaatCvAUfKttLearuWrP9MDH5MBPbIqV92AaeXatLxBI9gBaebbnrfifHhDYfgasaacH8akY=wiFfYdH8Gipec8Eeeu0xXdbba9frFj0=OqFfea0dXdd9vqai=hGuQ8kuc9pgc9s8qqaq=dirpe0xb9q8qiLsFr0=vr0=vr0dc8meaabaqaciaacaGaaeqabaqabeGadaaakeaacqGHuoarcqWGwbGvdaWgaaWcbaGafmiEaGNbaSaadaWgaaadbaGaeGymaedabeaaaSqabaaaaa@3227@ can be written as:

ϕ(x→,t,ΔVx→1)=4π∑l=0∞∑m=−ll12l+11|x→−x→1|l+1Ylm(θ1,φ1)Qlm(x→1,t)ΔVx→1     (4)
 MathType@MTEF@5@5@+=feaafiart1ev1aaatCvAUfKttLearuWrP9MDH5MBPbIqV92AaeXatLxBI9gBaebbnrfifHhDYfgasaacH8akY=wiFfYdH8Gipec8Eeeu0xXdbba9frFj0=OqFfea0dXdd9vqai=hGuQ8kuc9pgc9s8qqaq=dirpe0xb9q8qiLsFr0=vr0=vr0dc8meaabaqaciaacaGaaeqabaqabeGadaaakeaaiiGacqWFvpGAcqGGOaakcuWG4baEgaWcaiabcYcaSiabdsha0jabcYcaSiabgs5aejabdAfawnaaBaaaleaacuWG4baEgaWcamaaBaaameaacqaIXaqmaeqaaaWcbeaakiabcMcaPiabg2da9iabisda0iab=b8aWnaaqahabaWaaabCaeaadaWcaaqaaiabigdaXaqaaiabikdaYiabdYgaSjabgUcaRiabigdaXaaadaWcaaqaaiabigdaXaqaaiabcYha8jqbdIha4zaalaGaeyOeI0IafmiEaGNbaSaadaWgaaWcbaGaeGymaedabeaakiabcYha8naaCaaaleqabaGaemiBaWMaey4kaSIaeGymaedaaaaaaeaacqWGTbqBcqGH9aqpcqGHsislcqWGSbaBaeaacqWGSbaBa0GaeyyeIuoaaSqaaiabdYgaSjabg2da9iabicdaWaqaaiabg6HiLcqdcqGHris5aOGaemywaK1aaSbaaSqaaiabdYgaSjabd2gaTbqabaGccqGGOaakcqWF4oqCdaWgaaWcbaGaeGymaedabeaakiabcYcaSiab=z8aMnaaBaaaleaacqaIXaqmaeqaaOGaeiykaKIaemyuae1aaSbaaSqaaiabdYgaSjabd2gaTbqabaGccqGGOaakcuWG4baEgaWcamaaBaaaleaacqaIXaqmaeqaaOGaeiilaWIaemiDaqNaeiykaKIaeyiLdqKaemOvay1aaSbaaSqaaiqbdIha4zaalaWaaSbaaWqaaiabigdaXaqabaaaleqaaOGaaCzcaiaaxMaadaqadaqaaiabisda0aGaayjkaiaawMcaaaaa@7FCE@

where

Qlm(x→1,t)=1ΔVx→1∫ΔVx→1|x→″|lYlm∗(θ″,φ″)ρ(x→1+x→″,t)d3x→″     (5)
 MathType@MTEF@5@5@+=feaafiart1ev1aaatCvAUfKttLearuWrP9MDH5MBPbIqV92AaeXatLxBI9gBaebbnrfifHhDYfgasaacH8akY=wiFfYdH8Gipec8Eeeu0xXdbba9frFj0=OqFfea0dXdd9vqai=hGuQ8kuc9pgc9s8qqaq=dirpe0xb9q8qiLsFr0=vr0=vr0dc8meaabaqaciaacaGaaeqabaqabeGadaaakeaacqWGrbqudaWgaaWcbaGaemiBaWMaemyBa0gabeaakiabcIcaOiqbdIha4zaalaWaaSbaaSqaaiabigdaXaqabaGccqGGSaalcqWG0baDcqGGPaqkcqGH9aqpdaWcaaqaaiabigdaXaqaaiabgs5aejabdAfawnaaBaaaleaacuWG4baEgaWcamaaBaaameaacqaIXaqmaeqaaaWcbeaaaaGcdaWdrbqaaiabcYha8bWcbaGaeyiLdqKaemOvay1aaSbaaWqaaiqbdIha4zaalaWaaSbaaeaacqaIXaqmaeqaaaqabaaaleqaniabgUIiYdGccuWG4baEgaWcgaGbaiabcYha8naaCaaaleqabaGaemiBaWgaaOGaemywaK1aa0baaSqaaiabdYgaSjabd2gaTbqaaiabgEHiQaaakiabcIcaOGGaciqb=H7aXzaagaGaeiilaWIaf8NXdyMbayaacqGGPaqkcqWFbpGCcqGGOaakcuWG4baEgaWcamaaBaaaleaacqaIXaqmaeqaaOGaey4kaSIafmiEaGNbaSGbayaacqGGSaalcqWG0baDcqGGPaqkcqWGKbazdaahaaWcbeqaaiabiodaZaaakiqbdIha4zaalyaagaGaaCzcaiaaxMaadaqadaqaaiabiwda1aGaayjkaiaawMcaaaaa@6B05@

Q_lm _is the average multipole moment per unit volume at x→
 MathType@MTEF@5@5@+=feaafiart1ev1aaatCvAUfKttLearuWrP9MDH5MBPbIqV92AaeXatLxBI9gBaebbnrfifHhDYfgasaacH8akY=wiFfYdH8Gipec8Eeeu0xXdbba9frFj0=OqFfea0dXdd9vqai=hGuQ8kuc9pgc9s8qqaq=dirpe0xb9q8qiLsFr0=vr0=vr0dc8meaabaqaciaacaGaaeqabaqabeGadaaakeaacuWG4baEgaWcaaaa@2E37@_1 _and time t (ie the multipole moment density). It is important to understand the appropriate choice of the volume. It should be chosen to be large enough that each volume consists of a very similar collection of neural elements but small enough that its leading multipole moments are similar each time the region is activated. This restriction can be relaxed greatly without altering the underlying conclusions of this study if one considers the fields generated by coupled neural structures of different types. The total potential will simply be the sum of the potentials generated by the structures of each type.

Integrating (4) over all the volume elements in the sphere system yields the following expression for the total recorded potential as:

ϕ(x→,t)=4π∫V∑l=0∞∑m=−ll12l+11|x→−x→1|l+1Ylm(θ1,φ1)Qlm(x→1,t)d3x→1     (6)
 MathType@MTEF@5@5@+=feaafiart1ev1aaatCvAUfKttLearuWrP9MDH5MBPbIqV92AaeXatLxBI9gBaebbnrfifHhDYfgasaacH8akY=wiFfYdH8Gipec8Eeeu0xXdbba9frFj0=OqFfea0dXdd9vqai=hGuQ8kuc9pgc9s8qqaq=dirpe0xb9q8qiLsFr0=vr0=vr0dc8meaabaqaciaacaGaaeqabaqabeGadaaakeaaiiGacqWFvpGAcqGGOaakcuWG4baEgaWcaiabcYcaSiabdsha0jabcMcaPiabg2da9iabisda0iab=b8aWnaapefabaWaaabCaeaadaaeWbqaamaalaaabaGaeGymaedabaGaeGOmaiJaemiBaWMaey4kaSIaeGymaedaamaalaaabaGaeGymaedabaGaeiiFaWNafmiEaGNbaSaacqGHsislcuWG4baEgaWcamaaBaaaleaacqaIXaqmaeqaaOGaeiiFaW3aaWbaaSqabeaacqWGSbaBcqGHRaWkcqaIXaqmaaaaaaqaaiabd2gaTjabg2da9iabgkHiTiabdYgaSbqaaiabdYgaSbqdcqGHris5aaWcbaGaemiBaWMaeyypa0JaeGimaadabaGaeyOhIukaniabggHiLdGccqWGzbqwdaWgaaWcbaGaemiBaWMaemyBa0gabeaakiabcIcaOiab=H7aXnaaBaaaleaacqaIXaqmaeqaaOGaeiilaWIae8NXdy2aaSbaaSqaaiabigdaXaqabaGccqGGPaqkcqWGrbqudaWgaaWcbaGaemiBaWMaemyBa0gabeaakiabcIcaOiqbdIha4zaalaWaaSbaaSqaaiabigdaXaqabaGccqGGSaalcqWG0baDcqGGPaqkcqWGKbazdaahaaWcbeqaaiabiodaZaaakiqbdIha4zaalaWaaSbaaSqaaiabigdaXaqabaaabaGaemOvayfabeqdcqGHRiI8aOGaaCzcaiaaxMaadaqadaqaaiabiAda2aGaayjkaiaawMcaaaaa@7C89@

where (*θ*_1_, *φ*_1_) are the angles describing the location of the element under study relative to the center of the coordinate system. The advantage of this expansion over (1) is that in many neural systems the fields generated are dominated by either quadrupolar (l = 2) or dipolar (l = 1) components and so only theses components of the above sum are of practical importance.

### 2.3 The covariance function

One of the most commonly used descriptors of spontaneous electrical activity is the cross-covariance function. The goal of this section is to relate the patterns of multipole moment activation to the covariance between the signal recorded by an electrode of radius a and a similar electrode a distance s→
 MathType@MTEF@5@5@+=feaafiart1ev1aaatCvAUfKttLearuWrP9MDH5MBPbIqV92AaeXatLxBI9gBaebbnrfifHhDYfgasaacH8akY=wiFfYdH8Gipec8Eeeu0xXdbba9frFj0=OqFfea0dXdd9vqai=hGuQ8kuc9pgc9s8qqaq=dirpe0xb9q8qiLsFr0=vr0=vr0dc8meaabaqaciaacaGaaeqabaqabeGadaaakeaacuWGZbWCgaWcaaaa@2E2D@ from the first electrode embedded in spherical volume of radius R. One standard definition of the cross covariance function is:

νaR(x→,s→,τ)=〈(ϕ(x→,t)−〈ϕ(x→,t)〉)(ϕ(x→+s→,t+τ)−〈ϕ(x→+s→,t+τ)〉)〉     (7)
 MathType@MTEF@5@5@+=feaafiart1ev1aaatCvAUfKttLearuWrP9MDH5MBPbIqV92AaeXatLxBI9gBaebbnrfifHhDYfgasaacH8akY=wiFfYdH8Gipec8Eeeu0xXdbba9frFj0=OqFfea0dXdd9vqai=hGuQ8kuc9pgc9s8qqaq=dirpe0xb9q8qiLsFr0=vr0=vr0dc8meaabaqaciaacaGaaeqabaqabeGadaaakeaaiiGacqWF9oGBdaWgaaWcbaGaemyyaeMaemOuaifabeaakiabcIcaOiqbdIha4zaalaGaeiilaWIafm4CamNbaSaacqGGSaalcqWFepaDcqGGPaqkcqGH9aqpdaaadeqaamaabmaabaGae8x1dOMaeiikaGIafmiEaGNbaSaacqGGSaalcqWG0baDcqGGPaqkcqGHsisldaaadeqaaiab=v9aQjabcIcaOiqbdIha4zaalaGaeiilaWIaemiDaqNaeiykaKcacaGLPmIaayPkJaaacaGLOaGaayzkaaWaaeWaaeaacqWFvpGAcqGGOaakcuWG4baEgaWcaiabgUcaRiqbdohaZzaalaGaeiilaWIaemiDaqNaey4kaSIae8hXdqNaeiykaKIaeyOeI0YaaaWabeaacqWFvpGAcqGGOaakcuWG4baEgaWcaiabgUcaRiqbdohaZzaalaGaeiilaWIaemiDaqNaey4kaSIae8hXdqNaeiykaKcacaGLPmIaayPkJaaacaGLOaGaayzkaaaacaGLPmIaayPkJaGaaCzcaiaaxMaadaqadaqaaiabiEda3aGaayjkaiaawMcaaaaa@6FE3@

where the following notation is used:

〈f(t)〉=lim⁡T→∞12T∫−TTf(t)dt     (8)
MathType@MTEF@5@5@+=feaafiart1ev1aaatCvAUfKttLearuWrP9MDH5MBPbIqV92AaeXatLxBI9gBaebbnrfifHhDYfgasaacH8akY=wiFfYdH8Gipec8Eeeu0xXdbba9frFj0=OqFfea0dXdd9vqai=hGuQ8kuc9pgc9s8qqaq=dirpe0xb9q8qiLsFr0=vr0=vr0dc8meaabaqaciaacaGaaeqabaqabeGadaaakeaadaaadeqaaiabdAgaMjabcIcaOiabdsha0jabcMcaPaGaayzkJiaawQYiaiabg2da9maaxababaGagiiBaWMaeiyAaKMaeiyBa0galeaacqWGubavcqGHsgIRcqGHEisPaeqaaOWaaSaaaeaacqaIXaqmaeaacqaIYaGmcqWGubavaaWaa8qCaeaacqWGMbGzcqGGOaakcqWG0baDcqGGPaqkcqWGKbazcqWG0baDaSqaaiabgkHiTiabdsfaubqaaiabdsfaubqdcqGHRiI8aOGaaCzcaiaaxMaadaqadaqaaiabiIda4aGaayjkaiaawMcaaaaa@50D5@

for any function of time f(t). Substituting (6) into (7) yields:

νaR(x→,s→,τ)(4π)2=∫Vd3x→′1d3x→1∑l=0∞∑l′=0∞∑m=−ll∑m′=−l′l′12l+112l′+11|x→−x→1|l+1Ylm(θ1,φ1)Yl′m′(θ′1,φ′1)|x→+s→−x→′1|l′+1〈[Qlm(x→1,t)−〈Qlm(x→1,t)〉][Ql′m′(x→′1,t+τ)−〈Ql′m′(x→′1,t+τ)〉]〉     (9)
 MathType@MTEF@5@5@+=feaafiart1ev1aaatCvAUfKttLearuWrP9MDH5MBPbIqV92AaeXatLxBI9gBaebbnrfifHhDYfgasaacH8akY=wiFfYdH8Gipec8Eeeu0xXdbba9frFj0=OqFfea0dXdd9vqai=hGuQ8kuc9pgc9s8qqaq=dirpe0xb9q8qiLsFr0=vr0=vr0dc8meaabaqaciaacaGaaeqabaqabeGadaaakqaabeqaamaalaaabaacciGae8xVd42aaSbaaSqaaiabdggaHjabdkfasbqabaGccqGGOaakcuWG4baEgaWcaiabcYcaSiqbdohaZzaalaGaeiilaWIae8hXdqNaeiykaKcabaGaeiikaGIaeGinaqJae8hWdaNaeiykaKYaaWbaaSqabeaacqaIYaGmaaaaaOGaeyypa0dabaWaa8quaeaacqWGKbazdaahaaWcbeqaaiabiodaZaaakiqbdIha4zaalyaafaWaaSbaaSqaaiabigdaXaqabaGccqWGKbazdaahaaWcbeqaaiabiodaZaaakiqbdIha4zaalaWaaSbaaSqaaiabigdaXaqabaaabaGaemOvayfabeqdcqGHRiI8aOWaaabCaeaadaaeWbqaamaaqahabaWaaabCaeaadaWcaaqaaiabigdaXaqaaiabikdaYiabdYgaSjabgUcaRiabigdaXaaadaWcaaqaaiabigdaXaqaaiabikdaYiqbdYgaSzaafaGaey4kaSIaeGymaedaamaalaaabaGaeGymaedabaWaaqWaaeaacuWG4baEgaWcaiabgkHiTiqbdIha4zaalaWaaSbaaSqaaiabigdaXaqabaaakiaawEa7caGLiWoadaahaaWcbeqaaiabdYgaSjabgUcaRiabigdaXaaaaaaabaGafmyBa0MbauaacqGH9aqpcqGHsislcuWGSbaBgaqbaaqaaiqbdYgaSzaafaaaniabggHiLdaaleaacqWGTbqBcqGH9aqpcqGHsislcqWGSbaBaeaacqWGSbaBa0GaeyyeIuoaaSqaaiqbdYgaSzaafaGaeyypa0JaeGimaadabaGaeyOhIukaniabggHiLdaaleaacqWGSbaBcqGH9aqpcqaIWaamaeaacqGHEisPa0GaeyyeIuoakmaalaaabaGaemywaK1aaSbaaSqaaiabdYgaSjabd2gaTbqabaGccqGGOaakcqWF4oqCdaWgaaWcbaGaeGymaedabeaakiabcYcaSiab=z8aMnaaBaaaleaacqaIXaqmaeqaaOGaeiykaKIaemywaK1aaSbaaSqaaiqbdYgaSzaafaGafmyBa0MbauaaaeqaaOGaeiikaGIaf8hUdeNbauaadaWgaaWcbaGaeGymaedabeaakiabcYcaSiqb=z8aMzaafaWaaSbaaSqaaiabigdaXaqabaGccqGGPaqkaeaadaabdaqaaiqbdIha4zaalaGaey4kaSIafm4CamNbaSaacqGHsislcuWG4baEgaWcgaqbamaaBaaaleaacqaIXaqmaeqaaaGccaGLhWUaayjcSdWaaWbaaSqabeaacuWGSbaBgaqbaiabgUcaRiabigdaXaaaaaGcdaaadeqaamaadmaabaGaemyuae1aaSbaaSqaaiabdYgaSjabd2gaTbqabaGccqGGOaakcuWG4baEgaWcamaaBaaaleaacqaIXaqmaeqaaOGaeiilaWIaemiDaqNaeiykaKIaeyOeI0YaaaWabeaacqWGrbqudaWgaaWcbaGaemiBaWMaemyBa0gabeaakiabcIcaOiqbdIha4zaalaWaaSbaaSqaaiabigdaXaqabaGccqGGSaalcqWG0baDcqGGPaqkaiaawMYicaGLQmcaaiaawUfacaGLDbaadaWadaqaaiabdgfarnaaBaaaleaacuWGSbaBgaqbaiqbd2gaTzaafaaabeaakiabcIcaOiqbdIha4zaalyaafaWaaSbaaSqaaiabigdaXaqabaGccqGGSaalcqWG0baDcqGHRaWkcqWFepaDcqGGPaqkcqGHsisldaaadeqaaiabdgfarnaaBaaaleaacuWGSbaBgaqbaiqbd2gaTzaafaaabeaakiabcIcaOiqbdIha4zaalyaafaWaaSbaaSqaaiabigdaXaqabaGccqGGSaalcqWG0baDcqGHRaWkcqWFepaDcqGGPaqkaiaawMYicaGLQmcaaiaawUfacaGLDbaaaiaawMYicaGLQmcacaWLjaGaaCzcamaabmaabaGaeGyoaKdacaGLOaGaayzkaaaaaaa@EB15@

where V is now taken as the region bounded by the surface of both electrodes and the sphere of radius R which bounds the region filled with neural structures.

One very important simplification occurs when it can be assumed that the structure of each neural region remains constant over time and that the fluctuations in neural activity over time in each region just modify the magnitude of the multipole moments over time. In particular, this means that it is possible to write:

Qlm(x→1,t)=ℚlm(x→1)σ(x→1,t)     (10)
 MathType@MTEF@5@5@+=feaafiart1ev1aaatCvAUfKttLearuWrP9MDH5MBPbIqV92AaeXatLxBI9gBaebbnrfifHhDYfgasaacH8akY=wiFfYdH8Gipec8Eeeu0xXdbba9frFj0=OqFfea0dXdd9vqai=hGuQ8kuc9pgc9s8qqaq=dirpe0xb9q8qiLsFr0=vr0=vr0dc8meaabaqaciaacaGaaeqabaqabeGadaaakeaacqWGrbqudaWgaaWcbaGaemiBaWMaemyBa0gabeaakiabcIcaOiqbdIha4zaalaWaaSbaaSqaaiabigdaXaqabaGccqGGSaalcqWG0baDcqGGPaqkcqGH9aqpcqWIAecPdaWgaaWcbaGaemiBaWMaemyBa0gabeaakiabcIcaOiqbdIha4zaalaWaaSbaaSqaaiabigdaXaqabaGccqGGPaqkiiGacqWFdpWCcqGGOaakcuWG4baEgaWcamaaBaaaleaacqaIXaqmaeqaaOGaeiilaWIaemiDaqNaeiykaKIaaCzcaiaaxMaadaqadaqaaiabigdaXiabicdaWaGaayjkaiaawMcaaaaa@4E81@

where ℚ_*lm*_(x→
 MathType@MTEF@5@5@+=feaafiart1ev1aaatCvAUfKttLearuWrP9MDH5MBPbIqV92AaeXatLxBI9gBaebbnrfifHhDYfgasaacH8akY=wiFfYdH8Gipec8Eeeu0xXdbba9frFj0=OqFfea0dXdd9vqai=hGuQ8kuc9pgc9s8qqaq=dirpe0xb9q8qiLsFr0=vr0=vr0dc8meaabaqaciaacaGaaeqabaqabeGadaaakeaacuWG4baEgaWcaaaa@2E37@_1_) gives the magnitude of the maximal multipole moment generated when each region is fully activated and *σ*(x→
 MathType@MTEF@5@5@+=feaafiart1ev1aaatCvAUfKttLearuWrP9MDH5MBPbIqV92AaeXatLxBI9gBaebbnrfifHhDYfgasaacH8akY=wiFfYdH8Gipec8Eeeu0xXdbba9frFj0=OqFfea0dXdd9vqai=hGuQ8kuc9pgc9s8qqaq=dirpe0xb9q8qiLsFr0=vr0=vr0dc8meaabaqaciaacaGaaeqabaqabeGadaaakeaacuWG4baEgaWcaaaa@2E37@_1_, *t*) is a scalar function describing the degree of activation of neural elements at position x→
 MathType@MTEF@5@5@+=feaafiart1ev1aaatCvAUfKttLearuWrP9MDH5MBPbIqV92AaeXatLxBI9gBaebbnrfifHhDYfgasaacH8akY=wiFfYdH8Gipec8Eeeu0xXdbba9frFj0=OqFfea0dXdd9vqai=hGuQ8kuc9pgc9s8qqaq=dirpe0xb9q8qiLsFr0=vr0=vr0dc8meaabaqaciaacaGaaeqabaqabeGadaaakeaacuWG4baEgaWcaaaa@2E37@_1 _at time t. Note that in this representation, the orientation of a specific multipole moments is specified by the relative weights of the moment for the different values of m for a given value of 1 and so this theory also allows for arbitrary variations of the multipole moments from location to location. This means that:

〈[Qlm(x→1,t)−〈Qlm(x→1,t)〉][Ql′m′(x→1′,t+τ)−〈Ql′m′(x→1′,t+τ)〉]〉=ℚlm(x→1)ℚl′m′(x→1′)〈[σ(x→1,t)−〈σ(x→1,t)〉][σ(x→1′,t+τ)−〈σ(x→1′,t+τ)〉]〉     (11)
 MathType@MTEF@5@5@+=feaafiart1ev1aaatCvAUfKttLearuWrP9MDH5MBPbIqV92AaeXatLxBI9gBaebbnrfifHhDYfgasaacH8akY=wiFfYdH8Gipec8Eeeu0xXdbba9frFj0=OqFfea0dXdd9vqai=hGuQ8kuc9pgc9s8qqaq=dirpe0xb9q8qiLsFr0=vr0=vr0dc8meaabaqaciaacaGaaeqabaqabeGadaaakeaafaqaaeGabaaabaWaaaWabeaadaWadaqaaiabdgfarnaaBaaaleaacqWGSbaBcqWGTbqBaeqaaOGaeiikaGIafmiEaGNbaSaadaWgaaWcbaGaeGymaedabeaakiabcYcaSiabdsha0jabcMcaPiabgkHiTmaaamqabaGaemyuae1aaSbaaSqaaiabdYgaSjabd2gaTbqabaGccqGGOaakcuWG4baEgaWcamaaBaaaleaacqaIXaqmaeqaaOGaeiilaWIaemiDaqNaeiykaKcacaGLPmIaayPkJaaacaGLBbGaayzxaaWaamWaaeaacqWGrbqudaWgaaWcbaGafmiBaWMbauaacuWGTbqBgaqbaaqabaGccqGGOaakcuWG4baEgaWcamaaBaaaleaacqaIXaqmaeqaaGGaaOGae8NmGiQaeiilaWIaemiDaqNaey4kaSccciGae4hXdqNaeiykaKIaeyOeI0YaaaWabeaacqWGrbqudaWgaaWcbaGafmiBaWMbauaacuWGTbqBgaqbaaqabaGccqGGOaakcuWG4baEgaWcamaaBaaaleaacqaIXaqmaeqaaOGae8NmGiQaeiilaWIaemiDaqNaey4kaSIae4hXdqNaeiykaKcacaGLPmIaayPkJaaacaGLBbGaayzxaaaacaGLPmIaayPkJaaabaGaeyypa0JaeSOgHq6aaSbaaSqaaiabdYgaSjabd2gaTbqabaGccqGGOaakcuWG4baEgaWcamaaBaaaleaacqaIXaqmaeqaaOGaeiykaKIaeSOgHq6aaSbaaSqaaiqbdYgaSzaafaGafmyBa0MbauaaaeqaaOGaeiikaGIafmiEaGNbaSaadaWgaaWcbaGaeGymaedabeaakiab=jdiIkabcMcaPmaaamqabaWaamWaaeaacqGFdpWCcqGGOaakcuWG4baEgaWcamaaBaaaleaacqaIXaqmaeqaaOGaeiilaWIaemiDaqNaeiykaKIaeyOeI0YaaaWabeaacqGFdpWCcqGGOaakcuWG4baEgaWcamaaBaaaleaacqaIXaqmaeqaaOGaeiilaWIaemiDaqNaeiykaKcacaGLPmIaayPkJaaacaGLBbGaayzxaaWaamWaaeaacqGFdpWCcqGGOaakcuWG4baEgaWcamaaBaaaleaacqaIXaqmaeqaaOGae8NmGiQaeiilaWIaemiDaqNaey4kaSIae4hXdqNaeiykaKIaeyOeI0YaaaWabeaacqGFdpWCcqGGOaakcuWG4baEgaWcamaaBaaaleaacqaIXaqmaeqaaOGae8NmGiQaeiilaWIaemiDaqNaey4kaSIae4hXdqNaeiykaKcacaGLPmIaayPkJaaacaGLBbGaayzxaaaacaGLPmIaayPkJaaaaiaaxMaacaWLjaWaaeWaaeaacqaIXaqmcqaIXaqmaiaawIcacaGLPaaaaaa@B9CE@

Substituting into (10) yields:

νaR(x→,s→,τ)(4π)2=∫Vd3x→′1d3x→1∑l=0∞∑l′=0∞∑m=−ll∑m′=−l′l′12l+112l′+1Ylm(θ1,φ1)Yl′m′(θ′1,φ′1)|x→−x→1|l+1ℚlm(x→1)ℚl′m′(x→1′)|x→+s→−x→′1|l′+1g(x→1−x→1′,x→1,τ)     (12)
 MathType@MTEF@5@5@+=feaafiart1ev1aaatCvAUfKttLearuWrP9MDH5MBPbIqV92AaeXatLxBI9gBaebbnrfifHhDYfgasaacH8akY=wiFfYdH8Gipec8Eeeu0xXdbba9frFj0=OqFfea0dXdd9vqai=hGuQ8kuc9pgc9s8qqaq=dirpe0xb9q8qiLsFr0=vr0=vr0dc8meaabaqaciaacaGaaeqabaqabeGadaaakqaabeqaamaalaaabaacciGae8xVd42aaSbaaSqaaiabdggaHjabdkfasbqabaGccqGGOaakcuWG4baEgaWcaiabcYcaSiqbdohaZzaalaGaeiilaWIae8hXdqNaeiykaKcabaGaeiikaGIaeGinaqJae8hWdaNaeiykaKYaaWbaaSqabeaacqaIYaGmaaaaaOGaeyypa0dabaWaa8quaeaacqWGKbazdaahaaWcbeqaaiabiodaZaaakiqbdIha4zaalyaafaWaaSbaaSqaaiabigdaXaqabaGccqWGKbazdaahaaWcbeqaaiabiodaZaaakiqbdIha4zaalaWaaSbaaSqaaiabigdaXaqabaGcdaaeWbqaamaaqahabaWaaabCaeaadaaeWbqaamaalaaabaGaeGymaedabaGaeGOmaiJaemiBaWMaey4kaSIaeGymaedaamaalaaabaGaeGymaedabaGaeGOmaiJafmiBaWMbauaacqGHRaWkcqaIXaqmaaaaleaacuWGTbqBgaqbaiabg2da9iabgkHiTiqbdYgaSzaafaaabaGafmiBaWMbauaaa0GaeyyeIuoaaSqaaiabd2gaTjabg2da9iabgkHiTiabdYgaSbqaaiabdYgaSbqdcqGHris5aaWcbaGafmiBaWMbauaacqGH9aqpcqaIWaamaeaacqGHEisPa0GaeyyeIuoaaSqaaiabdYgaSjabg2da9iabicdaWaqaaiabg6HiLcqdcqGHris5aaWcbaGaemOvayfabeqdcqGHRiI8aOWaaSaaaeaacqWGzbqwdaWgaaWcbaGaemiBaWMaemyBa0gabeaakiabcIcaOiab=H7aXnaaBaaaleaacqaIXaqmaeqaaOGaeiilaWIae8NXdy2aaSbaaSqaaiabigdaXaqabaGccqGGPaqkcqWGzbqwdaWgaaWcbaGafmiBaWMbauaacuWGTbqBgaqbaaqabaGccqGGOaakcuWF4oqCgaqbamaaBaaaleaacqaIXaqmaeqaaOGaeiilaWIaf8NXdyMbauaadaWgaaWcbaacbaGae4xmaedabeaakiabcMcaPaqaaiabcYha8jqbdIha4zaalaGaeyOeI0IafmiEaGNbaSaadaWgaaWcbaGaeGymaedabeaakiabcYha8naaCaaaleqabaGaemiBaWMaey4kaSIaeGymaedaaaaakmaalaaabaGaeSOgHq6aaSbaaSqaaiabdYgaSjabd2gaTbqabaGccqGGOaakcuWG4baEgaWcamaaBaaaleaacqaIXaqmaeqaaOGaeiykaKIaeSOgHq6aaSbaaSqaaiqbdYgaSzaafaGafmyBa0MbauaaaeqaaOGaeiikaGIafmiEaGNbaSaadaWgaaWcbaGaeGymaedabeaaiiaakiab9jdiIkabcMcaPaqaaiabcYha8jqbdIha4zaalaGaey4kaSIafm4CamNbaSaacqGHsislcuWG4baEgaWcgaqbamaaBaaaleaacqaIXaqmaeqaaOGaeiiFaW3aaWbaaSqabeaacuWGSbaBgaqbaiabgUcaRiabigdaXaaaaaGccqWGNbWzcqGGOaakcuWG4baEgaWcamaaBaaaleaacqaIXaqmaeqaaOGaeyOeI0IafmiEaGNbaSaadaWgaaWcbaGaeGymaedabeaakiab9jdiIkabcYcaSiqbdIha4zaalaWaaSbaaSqaaiabigdaXaqabaGccqGGSaalcqWFepaDcqGGPaqkcaWLjaGaaCzcamaabmaabaGaeGymaeJaeGOmaidacaGLOaGaayzkaaaaaaa@D2F6@

where:

g(x→1−x→1′,x→1,τ)=〈[σ(x→1,t)−〈σ(x→1,t)〉][σ(x→1′,t+τ)−〈σ(x→1′,t+τ)〉]〉     (13)
 MathType@MTEF@5@5@+=feaafiart1ev1aaatCvAUfKttLearuWrP9MDH5MBPbIqV92AaeXatLxBI9gBaebbnrfifHhDYfgasaacH8akY=wiFfYdH8Gipec8Eeeu0xXdbba9frFj0=OqFfea0dXdd9vqai=hGuQ8kuc9pgc9s8qqaq=dirpe0xb9q8qiLsFr0=vr0=vr0dc8meaabaqaciaacaGaaeqabaqabeGadaaakeaacqWGNbWzcqGGOaakcuWG4baEgaWcamaaBaaaleaacqaIXaqmaeqaaOGaeyOeI0IafmiEaGNbaSaadaWgaaWcbaGaeGymaedabeaaiiaakiab=jdiIkabcYcaSiqbdIha4zaalaWaaSbaaSqaaiabigdaXaqabaGccqGGSaaliiGacqGFepaDcqGGPaqkcqGH9aqpdaaadeqaamaadmaabaGae43WdmNaeiikaGIafmiEaGNbaSaadaWgaaWcbaGaeGymaedabeaakiabcYcaSiabdsha0jabcMcaPiabgkHiTmaaamqabaGae43WdmNaeiikaGIafmiEaGNbaSaadaWgaaWcbaGaeGymaedabeaakiabcYcaSiabdsha0jabcMcaPaGaayzkJiaawQYiaaGaay5waiaaw2faamaadmaabaGae43WdmNaeiikaGIafmiEaGNbaSaadaWgaaWcbaGaeGymaedabeaakiab=jdiIkabcYcaSiabdsha0jabgUcaRiab+r8a0jabcMcaPiabgkHiTmaaamqabaGae43WdmNaeiikaGIafmiEaGNbaSaadaWgaaWcbaGaeGymaedabeaakiab=jdiIkabcYcaSiabdsha0jabgUcaRiab+r8a0jabcMcaPaGaayzkJiaawQYiaaGaay5waiaaw2faaaGaayzkJiaawQYiaiaaxMaacaWLjaWaaeWaaeaacqaIXaqmcqaIZaWmaiaawIcacaGLPaaaaaa@78A3@

is the paired covariance between the level of activity in different regions of the neural network. It should be noted that the covariance has been assumed to depend only on the distance between the regions and the location of one of the regions.

Before passing onto the more general case discussed in Appendix B, it is instructive to consider the case in which the level of activation at in each region is totally uncorrelatated:

g(x→1−x→1′,x→1,τ)=δ(x→1−x→1′)h(x→1,τ)     (14)
 MathType@MTEF@5@5@+=feaafiart1ev1aaatCvAUfKttLearuWrP9MDH5MBPbIqV92AaeXatLxBI9gBaebbnrfifHhDYfgasaacH8akY=wiFfYdH8Gipec8Eeeu0xXdbba9frFj0=OqFfea0dXdd9vqai=hGuQ8kuc9pgc9s8qqaq=dirpe0xb9q8qiLsFr0=vr0=vr0dc8meaabaqaciaacaGaaeqabaqabeGadaaakeaacqWGNbWzcqGGOaakcuWG4baEgaWcamaaBaaaleaacqaIXaqmaeqaaOGaeyOeI0IafmiEaGNbaSaadaWgaaWcbaGaeGymaedabeaaiiaakiab=jdiIkabcYcaSiqbdIha4zaalaWaaSbaaSqaaiabigdaXaqabaGccqGGSaaliiGacqGFepaDcqGGPaqkcqGH9aqpcqGF0oazcqGGOaakcuWG4baEgaWcamaaBaaaleaacqaIXaqmaeqaaOGaeyOeI0IafmiEaGNbaSaadaWgaaWcbaGaeGymaedabeaakiab=jdiIkabcMcaPiabdIgaOjabcIcaOiqbdIha4zaalaWaaSbaaSqaaiabigdaXaqabaGccqGGSaalcqGFepaDcqGGPaqkcaWLjaGaaCzcamaabmaabaGaeGymaeJaeGinaqdacaGLOaGaayzkaaaaaa@56F0@

for any function *h*(x→
 MathType@MTEF@5@5@+=feaafiart1ev1aaatCvAUfKttLearuWrP9MDH5MBPbIqV92AaeXatLxBI9gBaebbnrfifHhDYfgasaacH8akY=wiFfYdH8Gipec8Eeeu0xXdbba9frFj0=OqFfea0dXdd9vqai=hGuQ8kuc9pgc9s8qqaq=dirpe0xb9q8qiLsFr0=vr0=vr0dc8meaabaqaciaacaGaaeqabaqabeGadaaakeaacuWG4baEgaWcaaaa@2E37@_1_, *τ*). This implies that:

νaR(x→,s→,τ)(4π)2=∫Vd3x→1∑l=0∞∑l′=0∞∑m=−ll∑m′=−l′l′12l+112l′+11|x→−x→1|l+11|x→−s→−x→1|l′+1Ylm(θ1,φ1)Yl′m′(θ1,φ1)ℚlm(x→1)ℚl′m′(x→1)h(x→1,τ)     (15)
 MathType@MTEF@5@5@+=feaafiart1ev1aaatCvAUfKttLearuWrP9MDH5MBPbIqV92AaeXatLxBI9gBaebbnrfifHhDYfgasaacH8akY=wiFfYdH8Gipec8Eeeu0xXdbba9frFj0=OqFfea0dXdd9vqai=hGuQ8kuc9pgc9s8qqaq=dirpe0xb9q8qiLsFr0=vr0=vr0dc8meaabaqaciaacaGaaeqabaqabeGadaaakqaabeqaamaalaaabaacciGae8xVd42aaSbaaSqaaiabdggaHjabdkfasbqabaGccqGGOaakcuWG4baEgaWcaiabcYcaSiqbdohaZzaalaGaeiilaWIae8hXdqNaeiykaKcabaGaeiikaGIaeGinaqJae8hWdaNaeiykaKYaaWbaaSqabeaacqaIYaGmaaaaaOGaeyypa0dabaWaa8quaeaacqWGKbazdaahaaWcbeqaaiabiodaZaaakiqbdIha4zaalaWaaSbaaSqaaiabigdaXaqabaGcdaaeWbqaamaaqahabaWaaabCaeaadaaeWbqaamaalaaabaGaeGymaedabaGaeGOmaiJaemiBaWMaey4kaSIaeGymaedaamaalaaabaGaeGymaedabaGaeGOmaiJafmiBaWMbauaacqGHRaWkcqaIXaqmaaWaaSaaaeaacqaIXaqmaeaacqGG8baFcuWG4baEgaWcaiabgkHiTiqbdIha4zaalaWaaSbaaSqaaiabigdaXaqabaGccqGG8baFdaahaaWcbeqaaiabdYgaSjabgUcaRiabigdaXaaaaaGcdaWcaaqaaiabigdaXaqaaiabcYha8jqbdIha4zaalaGaeyOeI0Iafm4CamNbaSaacqGHsislcuWG4baEgaWcamaaBaaaleaacqaIXaqmaeqaaOGaeiiFaW3aaWbaaSqabeaacuWGSbaBgaqbaiabgUcaRiabigdaXaaaaaaabaGafmyBa0MbauaacqGH9aqpcqGHsislcuWGSbaBgaqbaaqaaiqbdYgaSzaafaaaniabggHiLdaaleaacqWGTbqBcqGH9aqpcqGHsislcqWGSbaBaeaacqWGSbaBa0GaeyyeIuoaaSqaaiqbdYgaSzaafaGaeyypa0JaeGimaadabaGaeyOhIukaniabggHiLdaaleaacqWGSbaBcqGH9aqpcqaIWaamaeaacqGHEisPa0GaeyyeIuoaaSqaaiabdAfawbqab0Gaey4kIipakiabdMfaznaaBaaaleaacqWGSbaBcqWGTbqBaeqaaOGaeiikaGIae8hUde3aaSbaaSqaaiabigdaXaqabaGccqGGSaalcqWFgpGzdaWgaaWcbaGaeGymaedabeaakiabcMcaPiabdMfaznaaBaaaleaacuWGSbaBgaqbaiqbd2gaTzaafaaabeaakiabcIcaOiab=H7aXnaaBaaaleaacqaIXaqmaeqaaOGaeiilaWIae8NXdy2aaSbaaSqaaiabigdaXaqabaGccqGGPaqkcqWIAecPdaWgaaWcbaGaemiBaWMaemyBa0gabeaakiabcIcaOiqbdIha4zaalaWaaSbaaSqaaiabigdaXaqabaGccqGGPaqkcqWIAecPdaWgaaWcbaGafmiBaWMbauaacuWGTbqBgaqbaaqabaGccqGGOaakcuWG4baEgaWcamaaBaaaleaacqaIXaqmaeqaaOGaeiykaKIaemiAaGMaeiikaGIafmiEaGNbaSaadaWgaaWcbaGaeGymaedabeaakiabcYcaSiab=r8a0jabcMcaPiaaxMaacaWLjaWaaeWaaeaacqaIXaqmcqaI1aqnaiaawIcacaGLPaaaaaaa@C553@

In Appendix D, the situation where s→
 MathType@MTEF@5@5@+=feaafiart1ev1aaatCvAUfKttLearuWrP9MDH5MBPbIqV92AaeXatLxBI9gBaebbnrfifHhDYfgasaacH8akY=wiFfYdH8Gipec8Eeeu0xXdbba9frFj0=OqFfea0dXdd9vqai=hGuQ8kuc9pgc9s8qqaq=dirpe0xb9q8qiLsFr0=vr0=vr0dc8meaabaqaciaacaGaaeqabaqabeGadaaakeaacuWGZbWCgaWcaaaa@2E2D@ is not zero is discussed, but in the case where both s→
 MathType@MTEF@5@5@+=feaafiart1ev1aaatCvAUfKttLearuWrP9MDH5MBPbIqV92AaeXatLxBI9gBaebbnrfifHhDYfgasaacH8akY=wiFfYdH8Gipec8Eeeu0xXdbba9frFj0=OqFfea0dXdd9vqai=hGuQ8kuc9pgc9s8qqaq=dirpe0xb9q8qiLsFr0=vr0=vr0dc8meaabaqaciaacaGaaeqabaqabeGadaaakeaacuWGZbWCgaWcaaaa@2E2D@ = x→
 MathType@MTEF@5@5@+=feaafiart1ev1aaatCvAUfKttLearuWrP9MDH5MBPbIqV92AaeXatLxBI9gBaebbnrfifHhDYfgasaacH8akY=wiFfYdH8Gipec8Eeeu0xXdbba9frFj0=OqFfea0dXdd9vqai=hGuQ8kuc9pgc9s8qqaq=dirpe0xb9q8qiLsFr0=vr0=vr0dc8meaabaqaciaacaGaaeqabaqabeGadaaakeaacuWG4baEgaWcaaaa@2E37@ = 0:

νaR(τ)=(4π)2∫Vd3x→1∑l=0∞∑l′=0∞∑m=−ll∑m′=−l′l′12l+112l′+11|x→1|l+l′+2Ylm(θ1,φ1)Yl′m′(θ1,φ1)ℚlm(x→1)ℚl′m′(x→1)h(x→1,τ)     (16)
 MathType@MTEF@5@5@+=feaafiart1ev1aaatCvAUfKttLearuWrP9MDH5MBPbIqV92AaeXatLxBI9gBaebbnrfifHhDYfgasaacH8akY=wiFfYdH8Gipec8Eeeu0xXdbba9frFj0=OqFfea0dXdd9vqai=hGuQ8kuc9pgc9s8qqaq=dirpe0xb9q8qiLsFr0=vr0=vr0dc8meaabaqaciaacaGaaeqabaqabeGadaaakeaaiiGacqWF9oGBdaWgaaWcbaGaemyyaeMaemOuaifabeaakiabcIcaOiab=r8a0jabcMcaPiabg2da9iabcIcaOiabisda0iab=b8aWjabcMcaPmaaCaaaleqabaGaeGOmaidaaOWaa8quaeaacqWGKbazdaahaaWcbeqaaiabiodaZaaakiqbdIha4zaalaWaaSbaaSqaaiabigdaXaqabaGcdaaeWbqaamaaqahabaWaaabCaeaadaaeWbqaamaalaaabaGaeGymaedabaGaeGOmaiJaemiBaWMaey4kaSIaeGymaedaamaalaaabaGaeGymaedabaGaeGOmaiJafmiBaWMbauaacqGHRaWkcqaIXaqmaaWaaSaaaeaacqaIXaqmaeaacqGG8baFcuWG4baEgaWcamaaBaaaleaacqaIXaqmaeqaaOGaeiiFaW3aaWbaaSqabeaacqWGSbaBcqGHRaWkcuWGSbaBgaqbaiabgUcaRiabikdaYaaaaaaabaGafmyBa0MbauaacqGH9aqpcqGHsislcuWGSbaBgaqbaaqaaiqbdYgaSzaafaaaniabggHiLdaaleaacqWGTbqBcqGH9aqpcqGHsislcqWGSbaBaeaacqWGSbaBa0GaeyyeIuoaaSqaaiqbdYgaSzaafaGaeyypa0JaeGimaadabaGaeyOhIukaniabggHiLdaaleaacqWGSbaBcqGH9aqpcqaIWaamaeaacqGHEisPa0GaeyyeIuoaaSqaaiabdAfawbqab0Gaey4kIipakiabdMfaznaaBaaaleaacqWGSbaBcqWGTbqBaeqaaOGaeiikaGIae8hUde3aaSbaaSqaaiabigdaXaqabaGccqGGSaalcqWFgpGzdaWgaaWcbaGaeGymaedabeaakiabcMcaPiabdMfaznaaBaaaleaacuWGSbaBgaqbaiqbd2gaTzaafaaabeaakiabcIcaOiab=H7aXnaaBaaaleaacqaIXaqmaeqaaOGaeiilaWIae8NXdy2aaSbaaSqaaiabigdaXaqabaGccqGGPaqkcqWIAecPdaWgaaWcbaGaemiBaWMaemyBa0gabeaakiabcIcaOiqbdIha4zaalaWaaSbaaSqaaiabigdaXaqabaGccqGGPaqkcqWIAecPdaWgaaWcbaGafmiBaWMbauaacuWGTbqBgaqbaaqabaGccqGGOaakcuWG4baEgaWcamaaBaaaleaacqaIXaqmaeqaaOGaeiykaKIaemiAaGMaeiikaGIafmiEaGNbaSaadaWgaaWcbaGaeGymaedabeaakiabcYcaSiab=r8a0jabcMcaPiaaxMaacaWLjaWaaeWaaeaacqaIXaqmcqaI2aGnaiaawIcacaGLPaaaaaa@B13E@

The next simplification comes when dividing the volume integral into its radial and angular components:

∫Vd3x→=∫aR|x→|2d|x→|∫ΩdΩ∫ΩdΩ=∫02πdφ∫0πsin⁡(θ)dθ
MathType@MTEF@5@5@+=feaafiart1ev1aaatCvAUfKttLearuWrP9MDH5MBPbIqV92AaeXatLxBI9gBaebbnrfifHhDYfgasaacH8akY=wiFfYdH8Gipec8Eeeu0xXdbba9frFj0=OqFfea0dXdd9vqai=hGuQ8kuc9pgc9s8qqaq=dirpe0xb9q8qiLsFr0=vr0=vr0dc8meaabaqaciaacaGaaeqabaqabeGadaaakeaafaqaaeGabaaabaWaa8quaeaacqWGKbazaSqaaiabdAfawbqab0Gaey4kIipakmaaCaaaleqabaGaeG4mamdaaOGafmiEaGNbaSaacqGH9aqpdaWdXbqaaiabcYha8jqbdIha4zaalaGaeiiFaW3aaWbaaSqabeaacqaIYaGmaaGccqWGKbazcqGG8baFcuWG4baEgaWcaiabcYha8bWcbaGaemyyaegabaGaemOuaifaniabgUIiYdGcdaWdrbqaaiabdsgaKjabgM6axbWcbaGaeyyQdCfabeqdcqGHRiI8aaGcbaWaa8quaeaacqWGKbazcqGHPoWvaSqaaiabgM6axbqab0Gaey4kIipakiabg2da9maapehabaGaemizaqgaleaacqaIWaamaeaacqaIYaGmiiGacqWFapaCa0Gaey4kIipakiab=z8aMnaapehabaGagi4CamNaeiyAaKMaeiOBa4MaeiikaGIae8hUdeNaeiykaKIaemizaqMae8hUdehaleaacqaIWaamaeaacqWFapaCa0Gaey4kIipaaaaaaa@6D13@

Defining:

qll′(|x→1|,τ)=∑m=−ll∑m′=−l′l′12l+112l′+1∫Ω1dΩ1Ylm(θ1,φ1)Yl′m′(θ1,φ1)ℚlm(x→1)ℚl′m′(x→1)h(x→1,τ)     (17)
 MathType@MTEF@5@5@+=feaafiart1ev1aaatCvAUfKttLearuWrP9MDH5MBPbIqV92AaeXatLxBI9gBaebbnrfifHhDYfgasaacH8akY=wiFfYdH8Gipec8Eeeu0xXdbba9frFj0=OqFfea0dXdd9vqai=hGuQ8kuc9pgc9s8qqaq=dirpe0xb9q8qiLsFr0=vr0=vr0dc8meaabaqaciaacaGaaeqabaqabeGadaaakeaacqWGXbqCdaWgaaWcbaGaemiBaWMafmiBaWMbauaaaeqaaOGaeiikaGIaeiiFaWNafmiEaGNbaSaadaWgaaWcbaGaeGymaedabeaakiabcYha8jabcYcaSGGaciab=r8a0jabcMcaPiabg2da9maaqahabaWaaabCaeaadaWcaaqaaiabigdaXaqaaiabikdaYiabdYgaSjabgUcaRiabigdaXaaadaWcaaqaaiabigdaXaqaaiabikdaYiqbdYgaSzaafaGaey4kaSIaeGymaedaaaWcbaGafmyBa0MbauaacqGH9aqpcqGHsislcuWGSbaBgaqbaaqaaiqbdYgaSzaafaaaniabggHiLdaaleaacqWGTbqBcqGH9aqpcqGHsislcqWGSbaBaeaacqWGSbaBa0GaeyyeIuoakmaapefabaGaemizaqgaleaacqGHPoWvdaWgaaadbaGaeGymaedabeaaaSqab0Gaey4kIipakiabgM6axnaaBaaaleaacqaIXaqmaeqaaOGaemywaK1aaSbaaSqaaiabdYgaSjabd2gaTbqabaGccqGGOaakcqWF4oqCdaWgaaWcbaGaeGymaedabeaakiabcYcaSiab=z8aMnaaBaaaleaacqaIXaqmaeqaaOGaeiykaKIaemywaK1aaSbaaSqaaiqbdYgaSzaafaGafmyBa0MbauaaaeqaaOGaeiikaGIae8hUde3aaSbaaSqaaiabigdaXaqabaGccqGGSaalcqWFgpGzdaWgaaWcbaGaeGymaedabeaakiabcMcaPiablQriKoaaBaaaleaacqWGSbaBcqWGTbqBaeqaaOGaeiikaGIafmiEaGNbaSaadaWgaaWcbaGaeGymaedabeaakiabcMcaPiablQriKoaaBaaaleaacuWGSbaBgaqbaiqbd2gaTzaafaaabeaakiabcIcaOiqbdIha4zaalaWaaSbaaSqaaiabigdaXaqabaGccqGGPaqkcqWGObaAcqGGOaakcuWG4baEgaWcamaaBaaaleaacqaIXaqmaeqaaOGaeiilaWIae8hXdqNaeiykaKIaaCzcaiaaxMaadaqadaqaaiabigdaXiabiEda3aGaayjkaiaawMcaaaaa@9848@

it is possible to write:

νaR(τ)=(4π)2∑l=0∞∑l′=0∞∫aRd|x→1|1|x→1|l+l′qll′(|x→1|,τ)     (18)
MathType@MTEF@5@5@+=feaafiart1ev1aaatCvAUfKttLearuWrP9MDH5MBPbIqV92AaeXatLxBI9gBaebbnrfifHhDYfgasaacH8akY=wiFfYdH8Gipec8Eeeu0xXdbba9frFj0=OqFfea0dXdd9vqai=hGuQ8kuc9pgc9s8qqaq=dirpe0xb9q8qiLsFr0=vr0=vr0dc8meaabaqaciaacaGaaeqabaqabeGadaaakeaaiiGacqWF9oGBdaWgaaWcbaGaemyyaeMaemOuaifabeaakiabcIcaOiab=r8a0jabcMcaPiabg2da9iabcIcaOiabisda0iab=b8aWjabcMcaPmaaCaaaleqabaGaeGOmaidaaOWaaabCaeaadaaeWbqaamaapehabaGaemizaqgaleaacqWGHbqyaeaacqWGsbGua0Gaey4kIipaaSqaaiqbdYgaSzaafaGaeyypa0JaeGimaadabaGaeyOhIukaniabggHiLdaaleaacqWGSbaBcqGH9aqpcqaIWaamaeaacqGHEisPa0GaeyyeIuoakiabcYha8jqbdIha4zaalaWaaSbaaSqaaiabigdaXaqabaGccqGG8baFdaWcaaqaaiabigdaXaqaaiabcYha8jqbdIha4zaalaWaaSbaaSqaaiabigdaXaqabaGccqGG8baFdaahaaWcbeqaaiabdYgaSjabgUcaRiqbdYgaSzaafaaaaaaakiabdghaXnaaBaaaleaacqWGSbaBcuWGSbaBgaqbaaqabaGccqGGOaakcqGG8baFcuWG4baEgaWcamaaBaaaleaacqaIXaqmaeqaaOGaeiiFaWNaeiilaWIae8hXdqNaeiykaKIaaCzcaiaaxMaadaqadaqaaiabigdaXiabiIda4aGaayjkaiaawMcaaaaa@72E8@

Now, the angular integral in (17) is over a finite region and since the ℚ and *h*(x→
 MathType@MTEF@5@5@+=feaafiart1ev1aaatCvAUfKttLearuWrP9MDH5MBPbIqV92AaeXatLxBI9gBaebbnrfifHhDYfgasaacH8akY=wiFfYdH8Gipec8Eeeu0xXdbba9frFj0=OqFfea0dXdd9vqai=hGuQ8kuc9pgc9s8qqaq=dirpe0xb9q8qiLsFr0=vr0=vr0dc8meaabaqaciaacaGaaeqabaqabeGadaaakeaacuWG4baEgaWcaaaa@2E37@_1_, *τ*) are bounded then so is *q*_*ll'*_(|x→
 MathType@MTEF@5@5@+=feaafiart1ev1aaatCvAUfKttLearuWrP9MDH5MBPbIqV92AaeXatLxBI9gBaebbnrfifHhDYfgasaacH8akY=wiFfYdH8Gipec8Eeeu0xXdbba9frFj0=OqFfea0dXdd9vqai=hGuQ8kuc9pgc9s8qqaq=dirpe0xb9q8qiLsFr0=vr0=vr0dc8meaabaqaciaacaGaaeqabaqabeGadaaakeaacuWG4baEgaWcaaaa@2E37@_1_|, *τ*). Let:

|qll′(|x→1|,τ)|<qll′∗(τ);a≤|x→1|≤R     (19)
 MathType@MTEF@5@5@+=feaafiart1ev1aaatCvAUfKttLearuWrP9MDH5MBPbIqV92AaeXatLxBI9gBaebbnrfifHhDYfgasaacH8akY=wiFfYdH8Gipec8Eeeu0xXdbba9frFj0=OqFfea0dXdd9vqai=hGuQ8kuc9pgc9s8qqaq=dirpe0xb9q8qiLsFr0=vr0=vr0dc8meaabaqaciaacaGaaeqabaqabeGadaaakeaadaabdaqaaiabdghaXnaaBaaaleaacqWGSbaBcuWGSbaBgaqbaaqabaGcdaqadaqaamaaemaabaGafmiEaGNbaSaadaWgaaWcbaGaeGymaedabeaaaOGaay5bSlaawIa7aiabcYcaSGGaciab=r8a0bGaayjkaiaawMcaaaGaay5bSlaawIa7aiabgYda8iabdghaXnaaDaaaleaacqWGSbaBcuWGSbaBgaqbaaqaaiabgEHiQaaakiabcIcaOiab=r8a0jabcMcaPiabcUda7iabdggaHjabgsMiJoaaemaabaGafmiEaGNbaSaadaWgaaWcbaGaeGymaedabeaaaOGaay5bSlaawIa7aiabgsMiJkabdkfasjaaxMaacaWLjaWaaeWaaeaacqaIXaqmcqaI5aqoaiaawIcacaGLPaaaaaa@598A@

then:

|νaR(τ)|≤(4π)2∑l=0∞∑l′=0∞qll′∗(τ)∫aRd|x→1|1|x→1|l+l′≤(4π)2∑l=0∞∑l′=0∞qll′∗(τ)1l+l′−1(1al+l′−1−1Rl+l′−1)     (20)
MathType@MTEF@5@5@+=feaafiart1ev1aaatCvAUfKttLearuWrP9MDH5MBPbIqV92AaeXatLxBI9gBaebbnrfifHhDYfgasaacH8akY=wiFfYdH8Gipec8Eeeu0xXdbba9frFj0=OqFfea0dXdd9vqai=hGuQ8kuc9pgc9s8qqaq=dirpe0xb9q8qiLsFr0=vr0=vr0dc8meaabaqaciaacaGaaeqabaqabeGadaaakeaacqGG8baFiiGacqWF9oGBdaWgaaWcbaGaemyyaeMaemOuaifabeaakiabcIcaOiab=r8a0jabcMcaPiabcYha8jabgsMiJkabcIcaOiabisda0iab=b8aWjabcMcaPmaaCaaaleqabaGaeGOmaidaaOWaaabCaeaadaaeWbqaaiabdghaXnaaDaaaleaacqWGSbaBcuWGSbaBgaqbaaqaaiabgEHiQaaaaeaacuWGSbaBgaqbaiabg2da9iabicdaWaqaaiabg6HiLcqdcqGHris5aaWcbaGaemiBaWMaeyypa0JaeGimaadabaGaeyOhIukaniabggHiLdGccqGGOaakcqWFepaDcqGGPaqkdaWdXbqaaiabdsgaKjabcYha8jqbdIha4zaalaWaaSbaaSqaaiabigdaXaqabaGccqGG8baFdaWcaaqaaiabigdaXaqaaiabcYha8jqbdIha4zaalaWaaSbaaSqaaiabigdaXaqabaGccqGG8baFdaahaaWcbeqaaiabdYgaSjabgUcaRiqbdYgaSzaafaaaaaaaaeaacqWGHbqyaeaacqWGsbGua0Gaey4kIipakiabgsMiJkabcIcaOiabisda0iab=b8aWjabcMcaPmaaCaaaleqabaGaeGOmaidaaOWaaabCaeaadaaeWbqaaiabdghaXnaaDaaaleaacqWGSbaBcuWGSbaBgaqbaaqaaiabgEHiQaaaaeaacuWGSbaBgaqbaiabg2da9iabicdaWaqaaiabg6HiLcqdcqGHris5aaWcbaGaemiBaWMaeyypa0JaeGimaadabaGaeyOhIukaniabggHiLdGccqGGOaakcqWFepaDcqGGPaqkdaWcaaqaaiabigdaXaqaaiabdYgaSjabgUcaRiqbdYgaSzaafaGaeyOeI0IaeGymaedaamaabmaabaWaaSaaaeaacqaIXaqmaeaacqWGHbqydaahaaWcbeqaaiabdYgaSjabgUcaRiqbdYgaSzaafaGaeyOeI0IaeGymaedaaaaakiabgkHiTmaalaaabaGaeGymaedabaGaemOuai1aaWbaaSqabeaacqWGSbaBcqGHRaWkcuWGSbaBgaqbaiabgkHiTiabigdaXaaaaaaakiaawIcacaGLPaaacaWLjaGaaCzcamaabmaabaGaeGOmaiJaeGimaadacaGLOaGaayzkaaaaaa@A7EB@

Note that since the sources are either dipolar or quadrupolar then:

*q*_*ll' *_(|x→
 MathType@MTEF@5@5@+=feaafiart1ev1aaatCvAUfKttLearuWrP9MDH5MBPbIqV92AaeXatLxBI9gBaebbnrfifHhDYfgasaacH8akY=wiFfYdH8Gipec8Eeeu0xXdbba9frFj0=OqFfea0dXdd9vqai=hGuQ8kuc9pgc9s8qqaq=dirpe0xb9q8qiLsFr0=vr0=vr0dc8meaabaqaciaacaGaaeqabaqabeGadaaakeaacuWG4baEgaWcaaaa@2E37@_1_|, *τ*) = 0 unless l = l' = 1 or 2

so that l+l'-l > 1. This means that as R increases without limit the covariance function remains bounded by the quantity:

lim⁡x→∞|νaR(τ)|<(4π)2∑l=0∞∑l′=0∞qll′∗(τ)1l+l′−1(1al+l′−1)
 MathType@MTEF@5@5@+=feaafiart1ev1aaatCvAUfKttLearuWrP9MDH5MBPbIqV92AaeXatLxBI9gBaebbnrfifHhDYfgasaacH8akY=wiFfYdH8Gipec8Eeeu0xXdbba9frFj0=OqFfea0dXdd9vqai=hGuQ8kuc9pgc9s8qqaq=dirpe0xb9q8qiLsFr0=vr0=vr0dc8meaabaqaciaacaGaaeqabaqabeGadaaakeaadaWfqaqaaiGbcYgaSjabcMgaPjabc2gaTbWcbaGaemiEaGNaeyOKH4QaeyOhIukabeaakiabcYha8HGaciab=17aUnaaBaaaleaacqWGHbqycqWGsbGuaeqaaOGaeiikaGIae8hXdqNaeiykaKIaeiiFaWNaeyipaWJaeiikaGIaeGinaqJae8hWdaNaeiykaKYaaWbaaSqabeaacqaIYaGmaaGcdaaeWbqaamaaqahabaGaemyCae3aa0baaSqaaiabdYgaSjqbdYgaSzaafaaabaGaey4fIOcaaaqaaiqbdYgaSzaafaGaeyypa0JaeGimaadabaGaeyOhIukaniabggHiLdaaleaacqWGSbaBcqGH9aqpcqaIWaamaeaacqGHEisPa0GaeyyeIuoakiabcIcaOiab=r8a0jabcMcaPmaalaaabaGaeGymaedabaGaemiBaWMaey4kaSIafmiBaWMbauaacqGHsislcqaIXaqmaaWaaeWaaeaadaWcaaqaaiabigdaXaqaaiabdggaHnaaCaaaleqabaGaemiBaWMaey4kaSIafmiBaWMbauaacqGHsislcqaIXaqmaaaaaaGccaGLOaGaayzkaaaaaa@6E57@

which is finite. Thus, contributions to the autocovariance function from neural structures far from the electrode are small when the activity in different regions is not correlated and as expected from the prominent appearance of the radius of the electrode, *a*, is mainly determined by neural structures near the electrode. This conclusion is not influenced by the orientation or the distribution of multipoles within the volume as long as the multipole density remains finite.

In Appendix A, the signal autocovariance function *ν*_*aR *_(*τ*) is computed for a more general covariance function for integer values of p:

g(x→1−x→1′,x→1,τ)=∑p=pmin⁡pmax⁡hp(x→1,τ)|x→1−x→1′|p     (21)
 MathType@MTEF@5@5@+=feaafiart1ev1aaatCvAUfKttLearuWrP9MDH5MBPbIqV92AaeXatLxBI9gBaebbnrfifHhDYfgasaacH8akY=wiFfYdH8Gipec8Eeeu0xXdbba9frFj0=OqFfea0dXdd9vqai=hGuQ8kuc9pgc9s8qqaq=dirpe0xb9q8qiLsFr0=vr0=vr0dc8meaabaqaciaacaGaaeqabaqabeGadaaakeaacqWGNbWzcqGGOaakcuWG4baEgaWcamaaBaaaleaacqaIXaqmaeqaaOGaeyOeI0IafmiEaGNbaSaadaWgaaWcbaGaeGymaedabeaaiiaakiab=jdiIkabcYcaSiqbdIha4zaalaWaaSbaaSqaaiabigdaXaqabaGccqGGSaaliiGacqGFepaDcqGGPaqkcqGH9aqpdaaeWbqaamaalaaabaGaemiAaG2aaSbaaSqaaiabdchaWbqabaGccqGGOaakcuWG4baEgaWcamaaBaaaleaacqaIXaqmaeqaaOGaeiilaWIae4hXdqNaeiykaKcabaGaeiiFaWNafmiEaGNbaSaadaWgaaWcbaGaeGymaedabeaakiabgkHiTiqbdIha4zaalaWaaSbaaSqaaiabigdaXaqabaGccqWFYaIOcqGG8baFdaahaaWcbeqaaiabdchaWbaaaaaabaGaemiCaaNaeyypa0JaemiCaa3aaSbaaWqaaiGbc2gaTjabcMgaPjabc6gaUbqabaaaleaacqWGWbaCdaWgaaadbaGagiyBa0MaeiyyaeMaeiiEaGhabeaaa0GaeyyeIuoakiaaxMaacaWLjaWaaeWaaeaacqaIYaGmcqaIXaqmaiaawIcacaGLPaaaaaa@6A0F@

Although this mathematical argument is more complex, the conclusion is that quadrupolar (l = 2) sources far from an electrode will not significantly contribute to the signal covariance function as long as p_min _> 0 and p_max _is finite. For dipolar sources (l = 1) the neuronal correlation function, the autocovariance function in general does not have a defined limit as *R *→ ∞ for correlation functions of the form (21) with p_max _< 3 as required for convergence of the integrals required for computation of the autocorrelation function.

It is also possible to estimate the dependence of the cross covariance function on the distance between the recording electrodes. In Appendix C it is demonstrated that, in the case where the activity in various neural structures is uncorrelated (i.e. source correlation function of the type (14)), if the sources are dipolar (l, l' = 1) then νaR(0,s→,τ)(4π)2=1|s→|
 MathType@MTEF@5@5@+=feaafiart1ev1aaatCvAUfKttLearuWrP9MDH5MBPbIqV92AaeXatLxBI9gBaebbnrfifHhDYfgasaacH8akY=wiFfYdH8Gipec8Eeeu0xXdbba9frFj0=OqFfea0dXdd9vqai=hGuQ8kuc9pgc9s8qqaq=dirpe0xb9q8qiLsFr0=vr0=vr0dc8meaabaqaciaacaGaaeqabaqabeGadaaakeaadaWcaaqaaGGaciab=17aUnaaBaaaleaacqWGHbqycqWGsbGuaeqaaOGaeiikaGIaeGimaaJaeiilaWIafm4CamNbaSaacqGGSaalcqWFepaDcqGGPaqkaeaacqGGOaakcqaI0aancqWFapaCcqGGPaqkdaahaaWcbeqaaiabikdaYaaaaaGccqGH9aqpdaWcaaqaaiabigdaXaqaaiabcYha8jqbdohaZzaalaGaeiiFaWhaaaaa@44DA@. If the sources are quadrupolar, then l, l' = 2 and νaR(0,s→,τ)(4π)2=1|s→|3
 MathType@MTEF@5@5@+=feaafiart1ev1aaatCvAUfKttLearuWrP9MDH5MBPbIqV92AaeXatLxBI9gBaebbnrfifHhDYfgasaacH8akY=wiFfYdH8Gipec8Eeeu0xXdbba9frFj0=OqFfea0dXdd9vqai=hGuQ8kuc9pgc9s8qqaq=dirpe0xb9q8qiLsFr0=vr0=vr0dc8meaabaqaciaacaGaaeqabaqabeGadaaakeaadaWcaaqaaGGaciab=17aUnaaBaaaleaacqWGHbqycqWGsbGuaeqaaOGaeiikaGIaeGimaaJaeiilaWIafm4CamNbaSaacqGGSaalcqWFepaDcqGGPaqkaeaacqGGOaakcqaI0aancqWFapaCcqGGPaqkdaahaaWcbeqaaiabikdaYaaaaaGccqGH9aqpdaWcaaqaaiabigdaXaqaaiabcYha8jqbdohaZzaalaGaeiiFaW3aaWbaaSqabeaacqaIZaWmaaaaaaaa@45FB@. The critical observation is that even when the activity in the neural structures is uncorrelated, the measured cross covariance functions decline as power-law function of the distance between the electrodes and do not display the expected delta function behavior.

Furthermore, as shown in Appendix D, when the neuronal correlation function is of the power law form and the neuronal structures produce a predominantly dipolar field, the measured correlation declines more slowly with distance than the neuronal correlation function. In the setting of quadrupolar sources, the measured cross covariance function has the same dependence on the distance between the electrodes as the neuronal correlation function.

### 2.4 More complex recording electrodes

The arguments presented above and in Appendix A formally refer to recordings from a monopolar electrode. However, as demonstrated in Appendix B, similar conclusions can be drawn for a complex electrode whose potential is the linear combination of the potentials at a number of other electrodes. In particular, in Appendix B, it is demonstrated that bipolar recording of dipolar sources is equivalent to monopolar recording of quadrupolar sources. Thus, although monopolar recordings from highly correlated dipole sources (as characterized by 0 <*p*_min _≤ 2 in equation (21)) are dominated by sources distance from the electrode, bipolar recordings from such highly correlated dipole sources receive the largest contribution from sources close to the electrode. Thus, the recordings from different electrodes can be vastly different when they are recording from extended neural structures with long-range correlations.

### 2.5 The qualitative argument

In order to obtain a qualitative understanding of these mathematical arguments, it is helpful to return to the original argument of section 2.1. From the discussion above, it is clear that that the key element left out of the original argument was the finite range of neuronal correlations. It is instructive to consider a very crude qualitative argument which takes into account these finite range effects. Assume that the neural system can be divided up into discrete elements of volume v_0 _and label each element with the index i, then the recorded potential can be written:

V=ℚ0∑i=1N1rimσi
 MathType@MTEF@5@5@+=feaafiart1ev1aaatCvAUfKttLearuWrP9MDH5MBPbIqV92AaeXatLxBI9gBaebbnrfifHhDYfgasaacH8akY=wiFfYdH8Gipec8Eeeu0xXdbba9frFj0=OqFfea0dXdd9vqai=hGuQ8kuc9pgc9s8qqaq=dirpe0xb9q8qiLsFr0=vr0=vr0dc8meaabaqaciaacaGaaeqabaqabeGadaaakeaacqWGwbGvcqGH9aqpcqWIAecPdaWgaaWcbaGaeGimaadabeaakmaaqahabaWaaSaaaeaacqaIXaqmaeaacqWGYbGCdaqhaaWcbaGaemyAaKgabaGaemyBa0gaaaaaaeaacqWGPbqAcqGH9aqpcqaIXaqmaeaacqWGobGta0GaeyyeIuoaiiGakiab=n8aZnaaBaaaleaacqWGPbqAaeqaaaaa@40DB@

where ℚ_0 _is the moment associated with each element, r_i _is the distance of the i' th element from the electrode, *σ*_*i *_reflects that state of activation of the i'th element and N is the total number of elements:

N=43πR3V0
 MathType@MTEF@5@5@+=feaafiart1ev1aaatCvAUfKttLearuWrP9MDH5MBPbIqV92AaeXatLxBI9gBaebbnrfifHhDYfgasaacH8akY=wiFfYdH8Gipec8Eeeu0xXdbba9frFj0=OqFfea0dXdd9vqai=hGuQ8kuc9pgc9s8qqaq=dirpe0xb9q8qiLsFr0=vr0=vr0dc8meaabaqaciaacaGaaeqabaqabeGadaaakeaacqWGobGtcqGH9aqpdaWcaaqaamaalaaabaGaeGinaqdabaGaeG4mamdaaGGaciab=b8aWjabdkfasnaaCaaaleqabaGaeG4mamdaaaGcbaacbaGae4Nvay1aaSbaaSqaaiabicdaWaqabaaaaaaa@3750@

where R is the radius of the neural system. In order to estimate the potential, it is reasonable to note that most of the distances, r_i_, are on the order of R so that:

V≈ℚ0Rm∑i=1Nσi
 MathType@MTEF@5@5@+=feaafiart1ev1aaatCvAUfKttLearuWrP9MDH5MBPbIqV92AaeXatLxBI9gBaebbnrfifHhDYfgasaacH8akY=wiFfYdH8Gipec8Eeeu0xXdbba9frFj0=OqFfea0dXdd9vqai=hGuQ8kuc9pgc9s8qqaq=dirpe0xb9q8qiLsFr0=vr0=vr0dc8meaabaqaciaacaGaaeqabaqabeGadaaakeaacqWGwbGvcqGHijYUdaWcaaqaaiablQriKoaaBaaaleaacqaIWaamaeqaaaGcbaGaemOuai1aaWbaaSqabeaacqWGTbqBaaaaaOWaaabCaeaaiiGacqWFdpWCdaWgaaWcbaGaemyAaKgabeaaaeaacqWGPbqAcqGH9aqpcqaIXaqmaeaacqWGobGta0GaeyyeIuoaaaa@3EFB@

The signal variance is given by:

〈(V2−〈V〉)2〉≈(ℚ0Rm)2〈(∑i=1N[σi−〈σi〉])2〉
 MathType@MTEF@5@5@+=feaafiart1ev1aaatCvAUfKttLearuWrP9MDH5MBPbIqV92AaeXatLxBI9gBaebbnrfifHhDYfgasaacH8akY=wiFfYdH8Gipec8Eeeu0xXdbba9frFj0=OqFfea0dXdd9vqai=hGuQ8kuc9pgc9s8qqaq=dirpe0xb9q8qiLsFr0=vr0=vr0dc8meaabaqaciaacaGaaeqabaqabeGadaaakeaadaaadeqaamaabmaabaGaemOvay1aaWbaaSqabeaacqaIYaGmaaGccqGHsisldaaadeqaaiabdAfawbGaayzkJiaawQYiaaGaayjkaiaawMcaamaaCaaaleqabaGaeGOmaidaaaGccaGLPmIaayPkJaGaeyisIS7aaeWaaeaadaWcaaqaaiablQriKoaaBaaaleaacqaIWaamaeqaaaGcbaGaemOuai1aaWbaaSqabeaacqWGTbqBaaaaaaGccaGLOaGaayzkaaWaaWbaaSqabeaacqaIYaGmaaGcdaaadeqaamaabmaabaWaaabCaeaadaWadaqaaGGaciab=n8aZnaaBaaaleaacqWGPbqAaeqaaOGaeyOeI0YaaaWabeaacqWFdpWCdaWgaaWcbaGaemyAaKgabeaaaOGaayzkJiaawQYiaaGaay5waiaaw2faaaWcbaGaemyAaKMaeyypa0JaeGymaedabaGaemOta4eaniabggHiLdaakiaawIcacaGLPaaadaahaaWcbeqaaiabikdaYaaaaOGaayzkJiaawQYiaaaa@57ED@

Defining:

〈(σi−〈σi〉)2〉=σ02
 MathType@MTEF@5@5@+=feaafiart1ev1aaatCvAUfKttLearuWrP9MDH5MBPbIqV92AaeXatLxBI9gBaebbnrfifHhDYfgasaacH8akY=wiFfYdH8Gipec8Eeeu0xXdbba9frFj0=OqFfea0dXdd9vqai=hGuQ8kuc9pgc9s8qqaq=dirpe0xb9q8qiLsFr0=vr0=vr0dc8meaabaqaciaacaGaaeqabaqabeGadaaakeaadaaadeqaamaabmaabaacciGae83Wdm3aaSbaaSqaaiabdMgaPbqabaGccqGHsisldaaadeqaaiab=n8aZnaaBaaaleaacqWGPbqAaeqaaaGccaGLPmIaayPkJaaacaGLOaGaayzkaaWaaWbaaSqabeaacqaIYaGmaaaakiaawMYicaGLQmcacqGH9aqpcqWFdpWCdaqhaaWcbaGaeGimaadabaGaeGOmaidaaaaa@3F68@

the term:

〈(∑i=1N[σi−〈σi〉])2〉≈σ02NNc
 MathType@MTEF@5@5@+=feaafiart1ev1aaatCvAUfKttLearuWrP9MDH5MBPbIqV92AaeXatLxBI9gBaebbnrfifHhDYfgasaacH8akY=wiFfYdH8Gipec8Eeeu0xXdbba9frFj0=OqFfea0dXdd9vqai=hGuQ8kuc9pgc9s8qqaq=dirpe0xb9q8qiLsFr0=vr0=vr0dc8meaabaqaciaacaGaaeqabaqabeGadaaakeaadaaadeqaamaabmaabaWaaabCaeaadaWadaqaaGGaciab=n8aZnaaBaaaleaacqWGPbqAaeqaaOGaeyOeI0YaaaWabeaacqWFdpWCdaWgaaWcbaGaemyAaKgabeaaaOGaayzkJiaawQYiaaGaay5waiaaw2faaaWcbaGaemyAaKMaeyypa0JaeGymaedabaGaemOta4eaniabggHiLdaakiaawIcacaGLPaaadaahaaWcbeqaaiabikdaYaaaaOGaayzkJiaawQYiaiabgIKi7kab=n8aZnaaDaaaleaacqaIWaamaeaacqaIYaGmaaGccqWGobGtcqWGobGtdaWgaaWcbaGaem4yamgabeaaaaa@4C96@

where N_c _is the number of other elements with which the given element is correlated. If the correlation distance is R_C_, then:

Nc≈Min(43πR3V0,43πRc3V0)
 MathType@MTEF@5@5@+=feaafiart1ev1aaatCvAUfKttLearuWrP9MDH5MBPbIqV92AaeXatLxBI9gBaebbnrfifHhDYfgasaacH8akY=wiFfYdH8Gipec8Eeeu0xXdbba9frFj0=OqFfea0dXdd9vqai=hGuQ8kuc9pgc9s8qqaq=dirpe0xb9q8qiLsFr0=vr0=vr0dc8meaabaqaciaacaGaaeqabaqabeGadaaakeaacqWGobGtdaWgaaWcbaGaem4yamgabeaakiabgIKi7Iqaaiab=1eanjab=LgaPjab=5gaUnaabmaabaWaaSaaaeaadaWcaaqaaiabisda0aqaaiabiodaZaaaiiGacqGFapaCcqWGsbGudaahaaWcbeqaaiabiodaZaaaaOqaaiab=zfawnaaBaaaleaacqaIWaamaeqaaaaakiabcYcaSmaalaaabaWaaSaaaeaacqaI0aanaeaacqaIZaWmaaGae4hWdaNaemOuai1aa0baaSqaaiabdogaJbqaaiabiodaZaaaaOqaaiab=zfawnaaBaaaleaacqaIWaamaeqaaaaaaOGaayjkaiaawMcaaaaa@4987@

since the number of elements correlated with a given element can never be larger than the total number of elements in the system. Thus:

〈(V2−〈V〉)2〉=ℚ02(43πV0)2Min(R,Rc)3R2m−3     (22)
 MathType@MTEF@5@5@+=feaafiart1ev1aaatCvAUfKttLearuWrP9MDH5MBPbIqV92AaeXatLxBI9gBaebbnrfifHhDYfgasaacH8akY=wiFfYdH8Gipec8Eeeu0xXdbba9frFj0=OqFfea0dXdd9vqai=hGuQ8kuc9pgc9s8qqaq=dirpe0xb9q8qiLsFr0=vr0=vr0dc8meaabaqaciaacaGaaeqabaqabeGadaaakeaadaaadeqaamaabmaabaGaemOvay1aaWbaaSqabeaacqaIYaGmaaGccqGHsisldaaadeqaaiabdAfawbGaayzkJiaawQYiaaGaayjkaiaawMcaamaaCaaaleqabaGaeGOmaidaaaGccaGLPmIaayPkJaGaeyypa0JaeSOgHq6aa0baaSqaaiabicdaWaqaaiabikdaYaaakmaabmaabaWaaSaaaeaadaWcaaqaaiabisda0aqaaiabiodaZaaaiiGacqWFapaCaeaaieaacqGFwbGvdaWgaaWcbaGaeGimaadabeaaaaaakiaawIcacaGLPaaadaahaaWcbeqaaiabikdaYaaakmaalaaabaGaemyta0KaemyAaKMaemOBa4MaeiikaGIaemOuaiLaeiilaWIaemOuai1aaSbaaSqaaiabdogaJbqabaGccqGGPaqkdaahaaWcbeqaaiabiodaZaaaaOqaaiabdkfasnaaCaaaleqabaGaeGOmaiJaemyBa0MaeyOeI0IaeG4mamdaaaaakiaaxMaacaWLjaWaaeWaaeaacqaIYaGmcqaIYaGmaiaawIcacaGLPaaaaaa@5AC4@

so that:

〈(V2−〈V〉)2〉∼1R2m−6;R<<Rc〈(V2−〈V〉)2〉∼1R2m−3;R>>Rc
 MathType@MTEF@5@5@+=feaafiart1ev1aaatCvAUfKttLearuWrP9MDH5MBPbIqV92AaeXatLxBI9gBaebbnrfifHhDYfgasaacH8akY=wiFfYdH8Gipec8Eeeu0xXdbba9frFj0=OqFfea0dXdd9vqai=hGuQ8kuc9pgc9s8qqaq=dirpe0xb9q8qiLsFr0=vr0=vr0dc8meaabaqaciaacaGaaeqabaqabeGadaaakqaabeqaamaaamqabaWaaeWaaeaacqWGwbGvdaahaaWcbeqaaiabikdaYaaakiabgkHiTmaaamqabaGaemOvayfacaGLPmIaayPkJaaacaGLOaGaayzkaaWaaWbaaSqabeaacqaIYaGmaaaakiaawMYicaGLQmcacqWI8iIodaWcaaqaaiabigdaXaqaaiabdkfasnaaCaaaleqabaGaeGOmaiJaemyBa0MaeyOeI0IaeGOnaydaaaaakiabcUda7iabdkfasjabgYda8iabgYda8iabdkfasnaaBaaaleaacqWGJbWyaeqaaaGcbaWaaaWabeaadaqadaqaaiabdAfawnaaCaaaleqabaGaeGOmaidaaOGaeyOeI0YaaaWabeaacqWGwbGvaiaawMYicaGLQmcaaiaawIcacaGLPaaadaahaaWcbeqaaiabikdaYaaaaOGaayzkJiaawQYiaiablYJi6maalaaabaGaeGymaedabaGaemOuai1aaWbaaSqabeaacqaIYaGmcqWGTbqBcqGHsislcqaIZaWmaaaaaOGaei4oaSJaemOuaiLaeyOpa4JaeyOpa4JaemOuai1aaSbaaSqaaiabdogaJbqabaaaaaa@5FAF@

This qualitative argument suggests that recordings from dipole sources m = 2 are strongly dependent on the size of the neural system when the size of the system is much smaller than the correlation distance but is stable to changes in R when the system is much larger than the correlation distance. The recorded signal from quadrupolar sources m = 3 is independent of R only for the large systems while those from higher order moments *m *≥ 4 are always independent of R.

## 3. Discussion

The first result of this paper is that when there are strong long-range correlations between neural structures, the potential recorded at a monopolar electrode can be dominated by the activity in neural structures far from the electrode even if the potential from each group of neurons is of the "near field" type (declining as 1/r^2 ^or faster).

Specifically, when the correlations have a finite range R_C_, dominant contributions come from the distant neurons when the size of the system, R, is much smaller than R_C_. In the case where the underlying neuronal correlation functions have a power law dependence on the distance, it was found that when the correlation between electrical activity in different neuronal structures declines more slowly than 1/r^3^, the autocovariance function of the recorded signals from a monopolar electrode is dominated by activity from distant dipolar generators. Recordings from quadrupolar generators are dominated by neurons near the electrode as long as the correlation between the activity in neuronal structures decreases as 1/r^*ε *^with *ε *> **0**. Another important observation is that recordings of dipolar sources from bipolar electrodes have similar dependences on the size of the neural system as do monopolar recordings from quadrupolar sources. Thus, when there are long range correlations between the activity in neurons, it is possible that there may be major qualitative differences between the recordings made from pure monopolar and bipolar electrodes. This observation may have practical importance for the selection of the best electrodes for recording events associated with long-range correlations such as seizures or in finding the best electrodes with which to perform coherence analysis [9].

The second result of the analysis performed in this paper is the fact the dependence of the cross covariance of the electric potential recorded from physically separated electrodes has a very different distance dependence than the cross correlation function describing the activity in different neural structures. This is true for both dipolar and quadrupolar sources if the activity in the different neural structures is uncorrelated. However, when the underlying source correlation function has a power law structure, the measured cross covariance function is similar to the underlying neuronal correlation function when the sources are quadrupolar but decay more slowly with distance than the neuronal correlation function when the sources are dipolar. This, in conjunction with above discussion of the effect of different electrode types, suggests that recording with bipolar electrodes for studies of cross covariance functions will provide a better estimate of the underlying neuronal cross correlation function than recordings with monopolar electrodes.

The principles set forth in this paper are of interest only if there are actually long range correlations in real neural systems. Clear data on the range of neuronal correlations in humans is limited but coherences between widely separated parts of cortex have been demonstrated [7] using monopolar electrodes. In addition, the "global wave" theory of cortical oscillations proposed by Nunez [6] is based on estimations of length of cortico-cortical fibres in humans extending up to 10–20 cm. This estimate is simply the range of the connections between neurons. The actual spatial extent of correlations can be much longer. Neckelmann [10] has demonstrated correlations between neurons in cat brain decrease very slowly as a function of distance, extending beyond 1 cm and that the spatial extent of this correlation increases during seizures. Also, it should be noted that in simple statistical models of systems near critical points such as the Ising model with only nearest neighbor couplings, the correlation between spins separated by a distance R varies (in 3 dimensions) as 1R1+η
 MathType@MTEF@5@5@+=feaafiart1ev1aaatCvAUfKttLearuWrP9MDH5MBPbIqV92AaeXatLxBI9gBaebbnrfifHhDYfgasaacH8akY=wiFfYdH8Gipec8Eeeu0xXdbba9frFj0=OqFfea0dXdd9vqai=hGuQ8kuc9pgc9s8qqaq=dirpe0xb9q8qiLsFr0=vr0=vr0dc8meaabaqaciaacaGaaeqabaqabeGadaaakeaadaWcaaqaaiabigdaXaqaaiabdkfasnaaCaaaleqabaGaeGymaeJaey4kaSccciGae83TdGgaaaaaaaa@328B@ near the critical point where *η *≈ 0.028 [8]. This provides additional evidence for the likelihood that neural elements cold be synchronized over large distances especially when there are large scale coherent oscillations such as during a seizure.

It should be noted that, in actual applications, raw unprocessed data from electrodes is rarely used and a number of techniques are used to extract data that is considered most relevant for the particular application. One of the most common signal processing techniques is high pass filtering of the data. As described in a previous paper [5], the potentials from a quadrupolar sources moving at constant velocity such as those accompanying an action potential have a distance dependent power spectrum with the signal containing progressively lower frequencies as the distance between the source and recording electrode increases. Thus, if a fixed high pass filter is used, the actual contribution from moving quadrupoles (but not quadrupolar sources with fixed spatial location and varying intensity) may drop off faster than predicted by the model used in this paper and hence there is a reduced likelihood of contributions from distant structures to the recorded potential. However, dipolar sources typically occur at synapses, bends in axons, or regions where there is a change in axon diameter [5] and do not propagate. Hence, in neural systems dipole sources are generally in fixed positions and the result obtained in this paper is more likely to apply. Of course, there are a great number of other signal processing techniques that can be used to extract specific elements of the recorded signal and may be used to enhance the contributions from various structures if there is a priori information that distinguishes the recordings from these different structures.

## 4. Conclusion

Recordings of electrical activity from extended neural structures such as a brain are commonly used to understand the basic mechanisms underlying various brain functions. One critical question is whether the electrical activity that is recorded from an electrode comes from generators near the electrode or generators far from the electrode. The distinction between "near field" and "far field" potentials was introduced to describe situations in which a localized generator may produce responses that either decline very quickly or very slowly with distance from the generator. However, even if a single localized generator contributes minimally to the recorded electrical activity, the resultant effect of many correlated neurons distributed over a large region of space may be significant.

In this paper, it is demonstrated that for dipole sources in a uniform medium, the recorded potential is strongly dependent on the neurons far from the electrode when neuronal correlations drop more slowly than 1/r^3 ^or when the size of the neural structure is much smaller than a known correlation distance. Recordings from quadrupolar sources are strongly dependent on distant neurons when correlations drop more slowly than 1/r or the size of the system is much smaller than the correlation distance. Bipolar recordings from dipolar sources produce responses that have the same properties of quadrupolar sources and bipolar recordings of quadrupolar sources are always dominated by local generators.

In addition, it is demonstrated that the cross covariance functions computed from recordings of electric potential in extended neural systems do not reflect the underlying neuronal correlation function when correlations between neural generators are very short range. However, when the correlations between different neural regions decline as a power law function of distance, the measured cross covariance function declines more slowly with distance when the sources are dipolar. When the sources are quadrupolar, the recorded cross covariance function has similar distance dependence as the neuronal correlation function. This suggests that bipolar electrodes should be used for recording cross covariance functions in the setting of primarily dipolar sources.

## Appendix A-Power law neuronal correlation functions

The purpose of this appendix is to derive expressions for the autocovariance function of the recorded signal for a general class of neuronal correlation functions. As derived in the main text, the general relationship between the neuronal covariance function *g*(x→
 MathType@MTEF@5@5@+=feaafiart1ev1aaatCvAUfKttLearuWrP9MDH5MBPbIqV92AaeXatLxBI9gBaebbnrfifHhDYfgasaacH8akY=wiFfYdH8Gipec8Eeeu0xXdbba9frFj0=OqFfea0dXdd9vqai=hGuQ8kuc9pgc9s8qqaq=dirpe0xb9q8qiLsFr0=vr0=vr0dc8meaabaqaciaacaGaaeqabaqabeGadaaakeaacuWG4baEgaWcaaaa@2E37@_1 _- x→
 MathType@MTEF@5@5@+=feaafiart1ev1aaatCvAUfKttLearuWrP9MDH5MBPbIqV92AaeXatLxBI9gBaebbnrfifHhDYfgasaacH8akY=wiFfYdH8Gipec8Eeeu0xXdbba9frFj0=OqFfea0dXdd9vqai=hGuQ8kuc9pgc9s8qqaq=dirpe0xb9q8qiLsFr0=vr0=vr0dc8meaabaqaciaacaGaaeqabaqabeGadaaakeaacuWG4baEgaWcaaaa@2E37@_1_', x→
 MathType@MTEF@5@5@+=feaafiart1ev1aaatCvAUfKttLearuWrP9MDH5MBPbIqV92AaeXatLxBI9gBaebbnrfifHhDYfgasaacH8akY=wiFfYdH8Gipec8Eeeu0xXdbba9frFj0=OqFfea0dXdd9vqai=hGuQ8kuc9pgc9s8qqaq=dirpe0xb9q8qiLsFr0=vr0=vr0dc8meaabaqaciaacaGaaeqabaqabeGadaaakeaacuWG4baEgaWcaaaa@2E37@_1_, *τ*) and the signal autocovariance function *ν*_*aR*_(*τ*) is (13):

νaR(τ)(4π)2=∫Vd3x→′1d3x→1∑l=0∞∑l′=0∞∑m=−ll∑m′=−l′l′12l+112l′+1Ylm(θ1,φ1)Yl′m′(θ′1,φ′1)|x→1|l+1ℚlm(x→1)ℚl′m′(x→1′)|x→′1|l′+1g(x→1−x→1′,x→1,τ)     (23)
 MathType@MTEF@5@5@+=feaafiart1ev1aaatCvAUfKttLearuWrP9MDH5MBPbIqV92AaeXatLxBI9gBaebbnrfifHhDYfgasaacH8akY=wiFfYdH8Gipec8Eeeu0xXdbba9frFj0=OqFfea0dXdd9vqai=hGuQ8kuc9pgc9s8qqaq=dirpe0xb9q8qiLsFr0=vr0=vr0dc8meaabaqaciaacaGaaeqabaqabeGadaaakqaabeqaamaalaaabaacciGae8xVd42aaSbaaSqaaiabdggaHjabdkfasbqabaGccqGGOaakcqWFepaDcqGGPaqkaeaacqGGOaakcqaI0aancqWFapaCcqGGPaqkdaahaaWcbeqaaiabikdaYaaaaaGccqGH9aqpaeaadaWdrbqaaiabdsgaKnaaCaaaleqabaGaeG4mamdaaOGafmiEaGNbaSGbauaadaWgaaWcbaGaeGymaedabeaakiabdsgaKnaaCaaaleqabaGaeG4mamdaaOGafmiEaGNbaSaadaWgaaWcbaGaeGymaedabeaakmaaqahabaWaaabCaeaadaaeWbqaamaaqahabaWaaSaaaeaacqaIXaqmaeaacqaIYaGmcqWGSbaBcqGHRaWkcqaIXaqmaaWaaSaaaeaacqaIXaqmaeaacqaIYaGmcuWGSbaBgaqbaiabgUcaRiabigdaXaaaaSqaaiqbd2gaTzaafaGaeyypa0JaeyOeI0IafmiBaWMbauaaaeaacuWGSbaBgaqbaaqdcqGHris5aaWcbaGaemyBa0Maeyypa0JaeyOeI0IaemiBaWgabaGaemiBaWganiabggHiLdaaleaacuWGSbaBgaqbaiabg2da9iabicdaWaqaaiabg6HiLcqdcqGHris5aaWcbaGaemiBaWMaeyypa0JaeGimaadabaGaeyOhIukaniabggHiLdaaleaacqWGwbGvaeqaniabgUIiYdGcdaWcaaqaaiabdMfaznaaBaaaleaacqWGSbaBcqWGTbqBaeqaaOGaeiikaGIae8hUde3aaSbaaSqaaiabigdaXaqabaGccqGGSaalcqWFgpGzdaWgaaWcbaGaeGymaedabeaakiabcMcaPiabdMfaznaaBaaaleaacuWGSbaBgaqbaiqbd2gaTzaafaaabeaakiabcIcaOiqb=H7aXzaafaWaaSbaaSqaaiabigdaXaqabaGccqGGSaalcuWFgpGzgaqbamaaBaaaleaaieaacqGFXaqmaeqaaOGaeiykaKcabaGaeiiFaWNafmiEaGNbaSaadaWgaaWcbaGaeGymaedabeaakiabcYha8naaCaaaleqabaGaemiBaWMaey4kaSIaeGymaedaaaaakmaalaaabaGaeSOgHq6aaSbaaSqaaiabdYgaSjabd2gaTbqabaGccqGGOaakcuWG4baEgaWcamaaBaaaleaacqaIXaqmaeqaaOGaeiykaKIaeSOgHq6aaSbaaSqaaiqbdYgaSzaafaGafmyBa0MbauaaaeqaaOGaeiikaGIafmiEaGNbaSaadaWgaaWcbaGaeGymaedabeaaiiaakiab9jdiIkabcMcaPaqaaiabcYha8jqbdIha4zaalyaafaWaaSbaaSqaaiabigdaXaqabaGccqGG8baFdaahaaWcbeqaaiqbdYgaSzaafaGaey4kaSIaeGymaedaaaaakiabdEgaNjabcIcaOiqbdIha4zaalaWaaSbaaSqaaiabigdaXaqabaGccqGHsislcuWG4baEgaWcamaaBaaaleaacqaIXaqmaeqaaOGae0NmGiQaeiilaWIafmiEaGNbaSaadaWgaaWcbaGaeGymaedabeaakiabcYcaSiab=r8a0jabcMcaPiaaxMaacaWLjaWaaeWaaeaacqaIYaGmcqaIZaWmaiaawIcacaGLPaaaaaaa@C6DB@

Specifically, the dependence of *ν*_*aR *_(*τ*) on R will be studied as a function of p_min _for covariance functions of the form:

g(x→1−x→1′,x→1,τ)=∑p=pmin⁡pmax⁡hp(x→1,τ)|x→1−x→1′|p     (24)
 MathType@MTEF@5@5@+=feaafiart1ev1aaatCvAUfKttLearuWrP9MDH5MBPbIqV92AaeXatLxBI9gBaebbnrfifHhDYfgasaacH8akY=wiFfYdH8Gipec8Eeeu0xXdbba9frFj0=OqFfea0dXdd9vqai=hGuQ8kuc9pgc9s8qqaq=dirpe0xb9q8qiLsFr0=vr0=vr0dc8meaabaqaciaacaGaaeqabaqabeGadaaakeaacqWGNbWzcqGGOaakcuWG4baEgaWcamaaBaaaleaacqaIXaqmaeqaaOGaeyOeI0IafmiEaGNbaSaadaWgaaWcbaGaeGymaedabeaaiiaakiab=jdiIkabcYcaSiqbdIha4zaalaWaaSbaaSqaaiabigdaXaqabaGccqGGSaaliiGacqGFepaDcqGGPaqkcqGH9aqpdaaeWbqaamaalaaabaGaemiAaG2aaSbaaSqaaiabdchaWbqabaGccqGGOaakcuWG4baEgaWcamaaBaaaleaacqaIXaqmaeqaaOGaeiilaWIae4hXdqNaeiykaKcabaGaeiiFaWNafmiEaGNbaSaadaWgaaWcbaGaeGymaedabeaakiabgkHiTiqbdIha4zaalaWaaSbaaSqaaiabigdaXaqabaGccqWFYaIOcqGG8baFdaahaaWcbeqaaiabdchaWbaaaaaabaGaemiCaaNaeyypa0JaemiCaa3aaSbaaWqaaiGbc2gaTjabcMgaPjabc6gaUbqabaaaleaacqWGWbaCdaWgaaadbaGagiyBa0MaeiyyaeMaeiiEaGhabeaaa0GaeyyeIuoakiaaxMaacaWLjaWaaeWaaeaacqaIYaGmcqaI0aanaiaawIcacaGLPaaaaaa@6A15@

for some integers p_max _and p_min _> 0. It is important to realize that the degree to which the spatial extent of the covariance is strongly dependent on the value of p_min_. If p_min _is zero, the correlation extends over all spaces and decreases more quickly with distance as the value of p_min _increases. It is also important to note that p_max _must be less than 3 or else the integrals to be discussed below do not converge. Although a more general expression in terms of Gegenbauer polynomials is possible for non-integer values of p, attention will be focused on the case where p is restricted to integer values. In this case, equation (3) can be used to write:

g(x→1−x→1′,x→1,τ)=∑p=pmin⁡pmax⁡hp(x→1,τ)|x→1,x→1′|p=∑p=pmin⁡pmax⁡hp(x→1,τ)[4π∑L=0∞∑M=−LL12L+1{|x→′1|L|x→1|L+1;|x→′1|<|x→1||x→1|L|x→′1|L+1;|x→′1|≥|x→1|}YLM∗(θ1′,φ1′)YLM(θ1,φ1)]p=∑p=pmin⁡pmax⁡hp(x→1,τ)(4π)p∑Lp=0∞∑Mp=−LpLp⋯∑L1=0∞∑M1=−L1L1{|x→′1|∑i=1pLi|x→1|∑i=1pLi+p;|x→′1|<|x→1||x→1|∑i=1pLi|x→′1|∑i=1pLi+p;|x→′1|≥|x→1|}∏i=1p[12Li+1YLiMi∗(θ1′,φ1′)YLiMi(θ1,φ1)]     (25)
 MathType@MTEF@5@5@+=feaafiart1ev1aaatCvAUfKttLearuWrP9MDH5MBPbIqV92AaeXatLxBI9gBaebbnrfifHhDYfgasaacH8akY=wiFfYdH8Gipec8Eeeu0xXdbba9frFj0=OqFfea0dXdd9vqai=hGuQ8kuc9pgc9s8qqaq=dirpe0xb9q8qiLsFr0=vr0=vr0dc8meaabaqaciaacaGaaeqabaqabeGadaaakqaabeqaaiabdEgaNjabcIcaOiqbdIha4zaalaWaaSbaaSqaaiabigdaXaqabaGccqGHsislcuWG4baEgaWcamaaBaaaleaacqaIXaqmaeqaaGGaaOGae8NmGiQaeiilaWIafmiEaGNbaSaadaWgaaWcbaGaeGymaedabeaakiabcYcaSGGaciab+r8a0jabcMcaPiabg2da9maaqahabaWaaSaaaeaacqWGObaAdaWgaaWcbaGaemiCaahabeaakiabcIcaOiqbdIha4zaalaWaaSbaaSqaaiabigdaXaqabaGccqGGSaalcqGFepaDcqGGPaqkaeaadaabdaqaaiqbdIha4zaalaWaaSbaaSqaaiabigdaXaqabaGccqGGSaalcuWG4baEgaWcamaaBaaaleaacqaIXaqmaeqaaOGae8NmGikacaGLhWUaayjcSdWaaWbaaSqabeaacqWGWbaCaaaaaaqaaiabdchaWjabg2da9iabdchaWnaaBaaameaacyGGTbqBcqGGPbqAcqGGUbGBaeqaaaWcbaGaemiCaa3aaSbaaWqaaiGbc2gaTjabcggaHjabcIha4bqabaaaniabggHiLdGccqGH9aqpdaaeWbqaaiabdIgaOnaaBaaaleaacqWGWbaCaeqaaOGaeiikaGIafmiEaGNbaSaadaWgaaWcbaGaeGymaedabeaakiabcYcaSiab+r8a0bWcbaGaemiCaaNaeyypa0JaemiCaa3aaSbaaWqaaiGbc2gaTjabcMgaPjabc6gaUbqabaaaleaacqWGWbaCdaWgaaadbaGagiyBa0MaeiyyaeMaeiiEaGhabeaaa0GaeyyeIuoakiabcMcaPmaadmaabaGaeGinaqJae4hWda3aaabCaeaadaaeWbqaamaalaaabaGaeGymaedabaGaeGOmaiJaemitaWKaey4kaSIaeGymaedaaaWcbaGaemyta0Kaeyypa0JaeyOeI0IaemitaWeabaGaemitaWeaniabggHiLdGcdaGadeqaauaabeqaceaaaeaadaWcaaqaamaaemaabaGafmiEaGNbaSGbauaadaWgaaWcbaGaeGymaedabeaaaOGaay5bSlaawIa7amaaCaaaleqabaGaemitaWeaaaGcbaWaaqWaaeaacuWG4baEgaWcamaaBaaaleaacqaIXaqmaeqaaaGccaGLhWUaayjcSdWaaWbaaSqabeaacqWGmbatcqGHRaWkcqaIXaqmaaaaaOGaei4oaSZaaqWaaeaacuWG4baEgaWcgaqbamaaBaaaleaacqaIXaqmaeqaaaGccaGLhWUaayjcSdGaeyipaWZaaqWaaeaacuWG4baEgaWcamaaBaaaleaacqaIXaqmaeqaaaGccaGLhWUaayjcSdaabaWaaSaaaeaadaabdaqaaiqbdIha4zaalaWaaSbaaSqaaiabigdaXaqabaaakiaawEa7caGLiWoadaahaaWcbeqaaiabdYeambaaaOqaamaaemaabaGafmiEaGNbaSGbauaadaWgaaWcbaGaeGymaedabeaaaOGaay5bSlaawIa7amaaCaaaleqabaGaemitaWKaey4kaSIaeGymaedaaaaakiabcUda7maaemaabaGafmiEaGNbaSGbauaadaWgaaWcbaGaeGymaedabeaaaOGaay5bSlaawIa7aiabgwMiZoaaemaabaGafmiEaGNbaSaadaWgaaWcbaGaeGymaedabeaaaOGaay5bSlaawIa7aaaaaiaawUhacaGL9baacqWGzbqwdaqhaaWcbaGaemitaWKaemyta0eabaGaey4fIOcaaOGaeiikaGIae4hUde3aaSbaaSqaaiabigdaXaqabaGccqWFYaIOcqGGSaalcqGFgpGzdaWgaaWcbaGaeGymaedabeaakiab=jdiIkabcMcaPiabdMfaznaaBaaaleaacqWGmbatcqWGnbqtaeqaaOGaeiikaGIae4hUde3aaSbaaSqaaiabigdaXaqabaGccqGGSaalcqGFgpGzdaWgaaWcbaGaeGymaedabeaakiabcMcaPaWcbaGaemitaWKaeyypa0JaeGimaadabaGaeyOhIukaniabggHiLdaakiaawUfacaGLDbaadaahaaWcbeqaaiabdchaWbaaaOqaaiabg2da9maaqahabaGaemiAaG2aaSbaaSqaaiabdchaWbqabaGccqGGOaakcuWG4baEgaWcamaaBaaaleaacqaIXaqmaeqaaOGaeiilaWIae4hXdqNaeiykaKIaeiikaGIaeGinaqJae4hWdaNaeiykaKYaaWbaaSqabeaacqWGWbaCaaaabaGaemiCaaNaeyypa0JaemiCaa3aaSbaaWqaaiGbc2gaTjabcMgaPjabc6gaUbqabaaaleaacqWGWbaCdaWgaaadbaGagiyBa0MaeiyyaeMaeiiEaGhabeaaa0GaeyyeIuoakmaaqahabaWaaabCaeaacqWIVlctdaaeWbqaamaaqahabaWaaiWabeaafaqabeGabaaabaWaaSaaaeaadaabdaqaaiqbdIha4zaalyaafaWaaSbaaSqaaiabigdaXaqabaaakiaawEa7caGLiWoadaahaaWcbeqaamaaqahabaGaemitaW0aaSbaaWqaaiabdMgaPbqabaaabaGaemyAaKMaeyypa0JaeGymaedabaGaemiCaahaoiabggHiLdaaaaGcbaWaaqWaaeaacuWG4baEgaWcamaaBaaaleaacqaIXaqmaeqaaaGccaGLhWUaayjcSdWaaWbaaSqabeaadaaeWbqaaiabdYeamnaaBaaameaacqWGPbqAaeqaaSGaey4kaSIaemiCaahameaacqWGPbqAcqGH9aqpcqaIXaqmaeaacqWGWbaCa4GaeyyeIuoaaaaaaOGaei4oaSZaaqWaaeaacuWG4baEgaWcgaqbamaaBaaaleaacqaIXaqmaeqaaaGccaGLhWUaayjcSdGaeyipaWZaaqWaaeaacuWG4baEgaWcamaaBaaaleaacqaIXaqmaeqaaaGccaGLhWUaayjcSdaabaWaaSaaaeaadaabdaqaaiqbdIha4zaalaWaaSbaaSqaaiabigdaXaqabaaakiaawEa7caGLiWoadaahaaWcbeqaamaaqahabaGaemitaW0aaSbaaWqaaiabdMgaPbqabaaabaGaemyAaKMaeyypa0JaeGymaedabaGaemiCaahaoiabggHiLdaaaaGcbaWaaqWaaeaacuWG4baEgaWcgaqbamaaBaaaleaacqaIXaqmaeqaaaGccaGLhWUaayjcSdWaaWbaaSqabeaadaaeWbqaaiabdYeamnaaBaaameaacqWGPbqAaeqaaSGaey4kaSIaemiCaahameaacqWGPbqAcqGH9aqpcqaIXaqmaeaacqWGWbaCa4GaeyyeIuoaaaaaaOGaei4oaSZaaqWaaeaacuWG4baEgaWcgaqbamaaBaaaleaacqaIXaqmaeqaaaGccaGLhWUaayjcSdGaeyyzIm7aaqWaaeaacuWG4baEgaWcamaaBaaaleaacqaIXaqmaeqaaaGccaGLhWUaayjcSdaaaaGaay5Eaiaaw2haamaarahabaWaamWaaeaadaWcaaqaaiabigdaXaqaaiabikdaYiabdYeamnaaBaaaleaacqWGPbqAaeqaaOGaey4kaSIaeGymaedaaiabdMfaznaaDaaaleaacqWGmbatdaWgaaadbaGaemyAaKgabeaaliabd2eannaaBaaameaacqWGPbqAaeqaaaWcbaGaey4fIOcaaOGaeiikaGIae4hUde3aaSbaaSqaaiabigdaXaqabaGccqWFYaIOcqGGSaalcqGFgpGzdaWgaaWcbaGaeGymaedabeaakiab=jdiIkabcMcaPiabdMfaznaaBaaaleaacqWGmbatdaWgaaadbaGaemyAaKgabeaaliabd2eannaaBaaameaacqWGPbqAaeqaaaWcbeaakiabcIcaOiab+H7aXnaaBaaaleaacqaIXaqmaeqaaOGaeiilaWIae4NXdy2aaSbaaSqaaiabigdaXaqabaGccqGGPaqkaiaawUfacaGLDbaaaSqaaiabdMgaPjabg2da9iabigdaXaqaaiabdchaWbqdcqGHpis1aaWcbaGaemyta00aaSbaaWqaaiabigdaXaqabaWccqGH9aqpcqGHsislcqWGmbatdaWgaaadbaGaeGymaedabeaaaSqaaiabdYeamnaaBaaameaacqaIXaqmaeqaaaqdcqGHris5aaWcbaGaemitaW0aaSbaaWqaaiabigdaXaqabaWccqGH9aqpcqaIWaamaeaacqGHEisPa0GaeyyeIuoaaSqaaiabd2eannaaBaaameaacqWGWbaCaeqaaSGaeyypa0JaeyOeI0IaemitaW0aaSbaaWqaaiabdchaWbqabaaaleaacqWGmbatdaWgaaadbaGaemiCaahabeaaa0GaeyyeIuoaaSqaaiabdYeamnaaBaaameaacqWGWbaCaeqaaSGaeyypa0JaeGimaadabaGaeyOhIukaniabggHiLdGccaWLjaGaaCzcamaabmaabaGaeGOmaiJaeGynaudacaGLOaGaayzkaaaaaaa@D812@

Defining:

G(p,l,l′,L1...Lp,|x→1|,|x→1′|,τ)=∫dΩ1dΩ1′12l+112l′+1hp(x→1,τ)∑m=−ll∑m′=−l′l′ℚlm(x→1)ℚl′m′(x→1′)Ylm(θ1,φ1)Yl′m′(θ′1,φ′1)∑M1=−L1L1⋯∑Mp=−LpLp∏i=1p[12Li+1YLiMi∗(θ1′,φ1′)YLiMi(θ1,φ1)]     (27)
 MathType@MTEF@5@5@+=feaafiart1ev1aaatCvAUfKttLearuWrP9MDH5MBPbIqV92AaeXatLxBI9gBaebbnrfifHhDYfgasaacH8akY=wiFfYdH8Gipec8Eeeu0xXdbba9frFj0=OqFfea0dXdd9vqai=hGuQ8kuc9pgc9s8qqaq=dirpe0xb9q8qiLsFr0=vr0=vr0dc8meaabaqaciaacaGaaeqabaqabeGadaaakqaabeqaaiabdEeahnaabmaabaGaemiCaaNaeiilaWIaemiBaWMaeiilaWIafmiBaWMbauaacqGGSaalcqWGmbatdaWgaaWcbaGaeGymaedabeaakiabc6caUiabc6caUiabc6caUiabdYeamnaaBaaaleaacqWGWbaCaeqaaOGaeiilaWYaaqWaaeaacuWG4baEgaWcamaaBaaaleaacqaIXaqmaeqaaaGccaGLhWUaayjcSdGaeiilaWYaaqWaaeaacuWG4baEgaWcamaaBaaaleaacqaIXaqmaeqaaGGaaOGae8NmGikacaGLhWUaayjcSdGaeiilaWccciGae4hXdqhacaGLOaGaayzkaaGaeyypa0Zaa8qaaeaacqWGKbazcqqHPoWvdaWgaaWcbaGaeGymaedabeaakiabdsgaKjabfM6axnaaBaaaleaacqaIXaqmaeqaaOGae8NmGikaleqabeqdcqGHRiI8aOWaaSaaaeaacqaIXaqmaeaacqaIYaGmcqWGSbaBcqGHRaWkcqaIXaqmaaWaaSaaaeaacqaIXaqmaeaacqaIYaGmcuWGSbaBgaqbaiabgUcaRiabigdaXaaacqWGObaAdaWgaaWcbaGaemiCaahabeaakiabcIcaOiqbdIha4zaalaWaaSbaaSqaaiabigdaXaqabaGccqGGSaalcqGFepaDcqGGPaqkaeaadaaeWbqaamaaqahabaWefv3ySLgznfgDOjdaryqr1ngBPrginfgDObcv39gaiqaacqqFAesudaWgaaWcbaGaemiBaWMaemyBa0gabeaaaeaacuWGTbqBgaqbaiabg2da9iabgkHiTiqbdYgaSzaafaaabaGafmiBaWMbauaaa0GaeyyeIuoaaSqaaiabd2gaTjabg2da9iabgkHiTiabdYgaSbqaaiabdYgaSbqdcqGHris5aOGaeiikaGIafmiEaGNbaSaadaWgaaWcbaGaeGymaedabeaakiabcMcaPiab9PrirnaaBaaaleaacuWGSbaBgaqbaiqbd2gaTzaafaaabeaakiabcIcaOiqbdIha4zaalaWaaSbaaSqaaiabigdaXaqabaGccqWFYaIOcqGGPaqkcqWGzbqwdaWgaaWcbaGaemiBaWMaemyBa0gabeaakiabcIcaOiab+H7aXnaaBaaaleaacqaIXaqmaeqaaOGaeiilaWIae4NXdy2aaSbaaSqaaiabigdaXaqabaGccqGGPaqkcqWGzbqwdaWgaaWcbaGafmiBaWMbauaacuWGTbqBgaqbaaqabaGccqGGOaakcuGF4oqCgaqbamaaBaaaleaacqaIXaqmaeqaaOGaeiilaWIaf4NXdyMbauaadaWgaaWcbaGaeGymaedabeaakiabcMcaPmaaqahabaGaeS47IW0aaabCaeaadaqeWbqaamaadmaabaWaaSaaaeaacqaIXaqmaeaacqaIYaGmcqWGmbatdaWgaaWcbaGaemyAaKgabeaakiabgUcaRiabigdaXaaacqWGzbqwdaqhaaWcbaGaemitaW0aaSbaaWqaaiabdMgaPbqabaWccqWGnbqtdaWgaaadbaGaemyAaKgabeaaaSqaaiabgEHiQaaakiabcIcaOiab+H7aXnaaBaaaleaacqaIXaqmaeqaaOGae8NmGiQaeiilaWIae4NXdy2aaSbaaSqaaiabigdaXaqabaGccqWFYaIOcqGGPaqkcqWGzbqwdaWgaaWcbaGaemitaW0aaSbaaWqaaiabdMgaPbqabaWccqWGnbqtdaWgaaadbaGaemyAaKgabeaaaSqabaGccqGGOaakcqGF4oqCdaWgaaWcbaGaeGymaedabeaakiabcYcaSiab+z8aMnaaBaaaleaacqaIXaqmaeqaaOGaeiykaKcacaGLBbGaayzxaaaaleaacqWGPbqAcqGH9aqpcqaIXaqmaeaacqWGWbaCa0Gaey4dIunaaSqaaiabd2eannaaBaaameaacqWGWbaCaeqaaSGaeyypa0JaeyOeI0IaemitaW0aaSbaaWqaaiabdchaWbqabaaaleaacqWGmbatdaWgaaadbaGaemiCaahabeaaa0GaeyyeIuoaaSqaaiabd2eannaaBaaameaacqaIXaqmaeqaaSGaeyypa0JaeyOeI0IaemitaW0aaSbaaWqaaiabigdaXaqabaaaleaacqWGmbatdaWgaaadbaGaeGymaedabeaaa0GaeyyeIuoakiaaxMaacaWLjaWaaeWaaeaacqaIYaGmcqaI3aWnaiaawIcacaGLPaaaaaaa@05DC@

the expression for the autocovariance function becomes:

νaR(τ)=∑p=pmin⁡pmax⁡(4π)2+p∫aR∫aRd|x→1|d|x→′1|∑l=0∞∑l′=0∞1|x→1|l−11|x→′1|l′−1∑Lp=0∞⋯∑L1=0∞{|x→′1|∑i=1pLi|x→1|∑i=1pLi+p;|x→′1|<|x→1||x→1|∑i=1pLi|x→′1|∑i=1pLi+p;|x→′1|≥|x→1|}G(p,l,l′,L1...Lp,|x→1|,|x→1′|,τ)     (27)
MathType@MTEF@5@5@+=feaafiart1ev1aaatCvAUfKttLearuWrP9MDH5MBPbIqV92AaeXatLxBI9gBaebbnrfifHhDYfgasaacH8akY=wiFfYdH8Gipec8Eeeu0xXdbba9frFj0=OqFfea0dXdd9vqai=hGuQ8kuc9pgc9s8qqaq=dirpe0xb9q8qiLsFr0=vr0=vr0dc8meaabaqaciaacaGaaeqabaqabeGadaaakqaabeqaaGGaciab=17aUnaaBaaaleaacqWGHbqycqWGsbGuaeqaaOGaeiikaGIae8hXdqNaeiykaKIaeyypa0dabaWaaabCaeaacqGGOaakcqaI0aancqWFapaCcqGGPaqkdaahaaWcbeqaaiabikdaYiabgUcaRiabdchaWbaaaeaacqWGWbaCcqGH9aqpcqWGWbaCdaWgaaadbaGagiyBa0MaeiyAaKMaeiOBa4gabeaaaSqaaiabdchaWnaaBaaameaacyGGTbqBcqGGHbqycqGG4baEaeqaaaqdcqGHris5aOWaa8qCaeaadaWdXbqaaiabdsgaKnaaemaabaGafmiEaGNbaSaadaWgaaWcbaGaeGymaedabeaaaOGaay5bSlaawIa7aiabdsgaKnaaemaabaGafmiEaGNbaSGbauaadaWgaaWcbaGaeGymaedabeaaaOGaay5bSlaawIa7aaWcbaGaemyyaegabaGaemOuaifaniabgUIiYdaaleaacqWGHbqyaeaacqWGsbGua0Gaey4kIipakmaaqahabaWaaabCaeaadaWcaaqaaiabigdaXaqaamaaemaabaGafmiEaGNbaSaadaWgaaWcbaGaeGymaedabeaaaOGaay5bSlaawIa7amaaCaaaleqabaGaemiBaWMaeyOeI0IaeGymaedaaaaakmaalaaabaGaeGymaedabaWaaqWaaeaacuWG4baEgaWcgaqbamaaBaaaleaacqaIXaqmaeqaaaGccaGLhWUaayjcSdWaaWbaaSqabeaacuWGSbaBgaqbaiabgkHiTiabigdaXaaaaaaabaGafmiBaWMbauaacqGH9aqpcqaIWaamaeaacqGHEisPa0GaeyyeIuoaaSqaaiabdYgaSjabg2da9iabicdaWaqaaiabg6HiLcqdcqGHris5aOWaaabCaeaacqWIVlctdaaeWbqaamaacmqabaqbaeqabiqaaaqaamaalaaabaWaaqWaaeaacuWG4baEgaWcgaqbamaaBaaaleaacqaIXaqmaeqaaaGccaGLhWUaayjcSdWaaWbaaSqabeaadaaeWbqaaiabdYeamnaaBaaameaacqWGPbqAaeqaaaqaaiabdMgaPjabg2da9iabigdaXaqaaiabdchaWbGdcqGHris5aaaaaOqaamaaemaabaGafmiEaGNbaSaadaWgaaWcbaGaeGymaedabeaaaOGaay5bSlaawIa7amaaCaaaleqabaWaaabCaeaacqWGmbatdaWgaaadbaGaemyAaKgabeaaliabgUcaRiabdchaWbadbaGaemyAaKMaeyypa0JaeGymaedabaGaemiCaahaoiabggHiLdaaaaaakiabcUda7maaemaabaGafmiEaGNbaSGbauaadaWgaaWcbaGaeGymaedabeaaaOGaay5bSlaawIa7aiabgYda8maaemaabaGafmiEaGNbaSaadaWgaaWcbaGaeGymaedabeaaaOGaay5bSlaawIa7aaqaamaalaaabaWaaqWaaeaacuWG4baEgaWcamaaBaaaleaacqaIXaqmaeqaaaGccaGLhWUaayjcSdWaaWbaaSqabeaadaaeWbqaaiabdYeamnaaBaaameaacqWGPbqAaeqaaaqaaiabdMgaPjabg2da9iabigdaXaqaaiabdchaWbGdcqGHris5aaaaaOqaamaaemaabaGafmiEaGNbaSGbauaadaWgaaWcbaGaeGymaedabeaaaOGaay5bSlaawIa7amaaCaaaleqabaWaaabCaeaacqWGmbatdaWgaaadbaGaemyAaKgabeaaliabgUcaRiabdchaWbadbaGaemyAaKMaeyypa0JaeGymaedabaGaemiCaahaoiabggHiLdaaaaaakiabcUda7maaemaabaGafmiEaGNbaSGbauaadaWgaaWcbaGaeGymaedabeaaaOGaay5bSlaawIa7aiabgwMiZoaaemaabaGafmiEaGNbaSaadaWgaaWcbaGaeGymaedabeaaaOGaay5bSlaawIa7aaaaaiaawUhacaGL9baaaSqaaiabdYeamnaaBaaameaacqaIXaqmaeqaaSGaeyypa0JaeGimaadabaGaeyOhIukaniabggHiLdaaleaacqWGmbatdaWgaaadbaGaemiCaahabeaaliabg2da9iabicdaWaqaaiabg6HiLcqdcqGHris5aOGaem4raCKaeiikaGIaemiCaaNaeiilaWIaemiBaWMaeiilaWIafmiBaWMbauaacqGGSaalcqWGmbatdaWgaaWcbaGaeGymaedabeaakiabc6caUiabc6caUiabc6caUiabdYeamnaaBaaaleaacqWGWbaCaeqaaOGaeiilaWYaaqWaaeaacuWG4baEgaWcamaaBaaaleaacqaIXaqmaeqaaaGccaGLhWUaayjcSdGaeiilaWYaaqWaaeaacuWG4baEgaWcamaaBaaaleaacqaIXaqmaeqaaGGaaOGae4NmGikacaGLhWUaayjcSdGaeiilaWIae8hXdqNaeiykaKIaaCzcaiaaxMaadaqadaqaaiabikdaYiabiEda3aGaayjkaiaawMcaaaaaaa@2444@

The first step is to look at the upper bound for the autocovariance function. There must be a function G˜
 MathType@MTEF@5@5@+=feaafiart1ev1aaatCvAUfKttLearuWrP9MDH5MBPbIqV92AaeXatLxBI9gBaebbnrfifHhDYfgasaacH8akY=wiFfYdH8Gipec8Eeeu0xXdbba9frFj0=OqFfea0dXdd9vqai=hGuQ8kuc9pgc9s8qqaq=dirpe0xb9q8qiLsFr0=vr0=vr0dc8meaabaqaciaacaGaaeqabaqabeGadaaakeaacuWGhbWrgaacaaaa@2DD2@(*p*, *l*, *l*', *L*_1_,...*L*_*p*_, *τ*) satisfying:

|G(p,l,l′,L1...Lp,|x→1|,|x→1′|,τ)|≤G˜(p,l,l′,L1...Lp,τ);a≤|x→1|≤R,a≤|x→1′|≤R
 MathType@MTEF@5@5@+=feaafiart1ev1aaatCvAUfKttLearuWrP9MDH5MBPbIqV92AaeXatLxBI9gBaebbnrfifHhDYfgasaacH8akY=wiFfYdH8Gipec8Eeeu0xXdbba9frFj0=OqFfea0dXdd9vqai=hGuQ8kuc9pgc9s8qqaq=dirpe0xb9q8qiLsFr0=vr0=vr0dc8meaabaqaciaacaGaaeqabaqabeGadaaakeaadaabdaqaaiabdEeahnaabmaabaGaemiCaaNaeiilaWIaemiBaWMaeiilaWIafmiBaWMbauaacqGGSaalcqWGmbatdaWgaaWcbaGaeGymaedabeaakiabc6caUiabc6caUiabc6caUiabdYeamnaaBaaaleaacqWGWbaCaeqaaOGaeiilaWYaaqWaaeaacuWG4baEgaWcamaaBaaaleaacqaIXaqmaeqaaaGccaGLhWUaayjcSdGaeiilaWYaaqWaaeaacuWG4baEgaWcamaaBaaaleaacqaIXaqmaeqaaGGaaOGae8NmGikacaGLhWUaayjcSdGaeiilaWccciGae4hXdqhacaGLOaGaayzkaaaacaGLhWUaayjcSdGaeyizImQafm4raCKbaGaadaqadaqaaiabdchaWjabcYcaSiabdYgaSjabcYcaSiqbdYgaSzaafaGaeiilaWIaemitaW0aaSbaaSqaaiabigdaXaqabaGccqGGUaGlcqGGUaGlcqGGUaGlcqWGmbatdaWgaaWcbaGaemiCaahabeaakiabcYcaSiab+r8a0bGaayjkaiaawMcaaiabcUda7iabdggaHjabgsMiJoaaemaabaGafmiEaGNbaSaadaWgaaWcbaGaeGymaedabeaaaOGaay5bSlaawIa7aiabgsMiJkabdkfasjabcYcaSiabdggaHjabgsMiJoaaemaabaGafmiEaGNbaSaadaWgaaWcbaGaeGymaedabeaakiab=jdiIcGaay5bSlaawIa7aiabgsMiJkabdkfasbaa@82DA@

since the integral defining G is over a bounded region and the integrand is finite everywhere as long as p is finite. This implies that

|νaR(τ)|≤∑p=1pmax⁡(4π)2+p∑l=0∞∑l′=0∞∑Lp=0∞⋯∑L1=0∞G˜(p,l,l′,L1...Lp,τ)∫aR∫aRd|x→1|d|x→′1|1|x→1|l−11|x→′1|l′−1{|x→′1|∑i=1pLi|x→1|∑i=1pLi+p;|x→′1|<|x→1||x→1|∑i=1pLi|x→′1|∑i=1pLi+p;|x→′1|≥|x→1|}     (28)
MathType@MTEF@5@5@+=feaafiart1ev1aaatCvAUfKttLearuWrP9MDH5MBPbIqV92AaeXatLxBI9gBaebbnrfifHhDYfgasaacH8akY=wiFfYdH8Gipec8Eeeu0xXdbba9frFj0=OqFfea0dXdd9vqai=hGuQ8kuc9pgc9s8qqaq=dirpe0xb9q8qiLsFr0=vr0=vr0dc8meaabaqaciaacaGaaeqabaqabeGadaaakqaabeqaamaaemaabaacciGae8xVd42aaSbaaSqaaiabdggaHjabdkfasbqabaGccqGGOaakcqWFepaDcqGGPaqkaiaawEa7caGLiWoacqGHKjYOaeaadaaeWbqaaiabcIcaOiabisda0iab=b8aWjabcMcaPmaaCaaaleqabaGaeGOmaiJaey4kaSIaemiCaahaaaqaaiabdchaWjabg2da9iabigdaXaqaaiabdchaWnaaBaaameaacyGGTbqBcqGGHbqycqGG4baEaeqaaaqdcqGHris5aOWaaabCaeaadaaeWbqaamaaqahabaGaeS47IW0aaabCaeaacuWGhbWrgaacaaWcbaGaemitaW0aaSbaaWqaaiabigdaXaqabaWccqGH9aqpcqaIWaamaeaacqGHEisPa0GaeyyeIuoaaSqaaiabdYeamnaaBaaameaacqWGWbaCaeqaaSGaeyypa0JaeGimaadabaGaeyOhIukaniabggHiLdGcdaqadaqaaiabdchaWjabcYcaSiabdYgaSjabcYcaSiqbdYgaSzaafaGaeiilaWIaemitaW0aaSbaaSqaaiabigdaXaqabaGccqGGUaGlcqGGUaGlcqGGUaGlcqWGmbatdaWgaaWcbaGaemiCaahabeaakiabcYcaSiab=r8a0bGaayjkaiaawMcaaaWcbaGafmiBaWMbauaacqGH9aqpcqaIWaamaeaacqGHEisPa0GaeyyeIuoaaSqaaiabdYgaSjabg2da9iabicdaWaqaaiabg6HiLcqdcqGHris5aOWaa8qCaeaadaWdXbqaaiabdsgaKnaaemaabaGafmiEaGNbaSaadaWgaaWcbaGaeGymaedabeaaaOGaay5bSlaawIa7aiabdsgaKnaaemaabaGafmiEaGNbaSGbauaadaWgaaWcbaGaeGymaedabeaaaOGaay5bSlaawIa7aaWcbaGaemyyaegabaGaemOuaifaniabgUIiYdaaleaacqWGHbqyaeaacqWGsbGua0Gaey4kIipakmaalaaabaGaeGymaedabaWaaqWaaeaacuWG4baEgaWcamaaBaaaleaacqaIXaqmaeqaaaGccaGLhWUaayjcSdWaaWbaaSqabeaacqWGSbaBcqGHsislcqaIXaqmaaaaaOWaaSaaaeaacqaIXaqmaeaadaabdaqaaiqbdIha4zaalyaafaWaaSbaaSqaaiabigdaXaqabaaakiaawEa7caGLiWoadaahaaWcbeqaaiqbdYgaSzaafaGaeyOeI0IaeGymaedaaaaakmaacmqabaqbaeqabiqaaaqaamaalaaabaWaaqWaaeaacuWG4baEgaWcgaqbamaaBaaaleaacqaIXaqmaeqaaaGccaGLhWUaayjcSdWaaWbaaSqabeaadaaeWbqaaiabdYeamnaaBaaameaacqWGPbqAaeqaaaqaaiabdMgaPjabg2da9iabigdaXaqaaiabdchaWbGdcqGHris5aaaaaOqaamaaemaabaGafmiEaGNbaSaadaWgaaWcbaGaeGymaedabeaaaOGaay5bSlaawIa7amaaCaaaleqabaWaaabCaeaacqWGmbatdaWgaaadbaGaemyAaKgabeaaliabgUcaRiabdchaWbadbaGaemyAaKMaeyypa0JaeGymaedabaGaemiCaahaoiabggHiLdaaaaaakiabcUda7maaemaabaGafmiEaGNbaSGbauaadaWgaaWcbaGaeGymaedabeaaaOGaay5bSlaawIa7aiabgYda8maaemaabaGafmiEaGNbaSaadaWgaaWcbaGaeGymaedabeaaaOGaay5bSlaawIa7aaqaamaalaaabaWaaqWaaeaacuWG4baEgaWcamaaBaaaleaacqaIXaqmaeqaaaGccaGLhWUaayjcSdWaaWbaaSqabeaadaaeWbqaaiabdYeamnaaBaaameaacqWGPbqAaeqaaaqaaiabdMgaPjabg2da9iabigdaXaqaaiabdchaWbGdcqGHris5aaaaaOqaamaaemaabaGafmiEaGNbaSGbauaadaWgaaWcbaGaeGymaedabeaaaOGaay5bSlaawIa7amaaCaaaleqabaWaaabCaeaacqWGmbatdaWgaaadbaGaemyAaKgabeaaliabgUcaRiabdchaWbadbaGaemyAaKMaeyypa0JaeGymaedabaGaemiCaahaoiabggHiLdaaaaaakiabcUda7maaemaabaGafmiEaGNbaSGbauaadaWgaaWcbaGaeGymaedabeaaaOGaay5bSlaawIa7aiabgwMiZoaaemaabaGafmiEaGNbaSaadaWgaaWcbaGaeGymaedabeaaaOGaay5bSlaawIa7aaaaaiaawUhacaGL9baacaWLjaGaaCzcamaabmaabaGaeGOmaiJaeGioaGdacaGLOaGaayzkaaaaaaa@1457@

in order to simplify the calculations let |x→
 MathType@MTEF@5@5@+=feaafiart1ev1aaatCvAUfKttLearuWrP9MDH5MBPbIqV92AaeXatLxBI9gBaebbnrfifHhDYfgasaacH8akY=wiFfYdH8Gipec8Eeeu0xXdbba9frFj0=OqFfea0dXdd9vqai=hGuQ8kuc9pgc9s8qqaq=dirpe0xb9q8qiLsFr0=vr0=vr0dc8meaabaqaciaacaGaaeqabaqabeGadaaakeaacuWG4baEgaWcaaaa@2E37@_1_| = *r*,|x→
 MathType@MTEF@5@5@+=feaafiart1ev1aaatCvAUfKttLearuWrP9MDH5MBPbIqV92AaeXatLxBI9gBaebbnrfifHhDYfgasaacH8akY=wiFfYdH8Gipec8Eeeu0xXdbba9frFj0=OqFfea0dXdd9vqai=hGuQ8kuc9pgc9s8qqaq=dirpe0xb9q8qiLsFr0=vr0=vr0dc8meaabaqaciaacaGaaeqabaqabeGadaaakeaacuWG4baEgaWcaaaa@2E37@'_1_| = *r' *and define L∗=∑i=1pLp
 MathType@MTEF@5@5@+=feaafiart1ev1aaatCvAUfKttLearuWrP9MDH5MBPbIqV92AaeXatLxBI9gBaebbnrfifHhDYfgasaacH8akY=wiFfYdH8Gipec8Eeeu0xXdbba9frFj0=OqFfea0dXdd9vqai=hGuQ8kuc9pgc9s8qqaq=dirpe0xb9q8qiLsFr0=vr0=vr0dc8meaabaqaciaacaGaaeqabaqabeGadaaakeaacqWGmbatdaahaaWcbeqaaiabgEHiQaaakiabg2da9maaqahabaGaemitaW0aaSbaaSqaaiabdchaWbqabaaabaGaemyAaKMaeyypa0JaeGymaedabaGaemiCaahaniabggHiLdaaaa@39A0@. It can be seen that the dependence of the covariance function on R will be determined by the integrals:

∫aR∫aRdrdr′1rl−11r′l′−1{r′L∗rL∗+p;r′<rrL∗r′L∗+p;r′≥r}=∫aRdr[∫ardr′r′L∗−l′+1rL∗+p+l−1+∫rRdr′rL∗−l+1r′L∗+p+l′−1]     (29)
MathType@MTEF@5@5@+=feaafiart1ev1aaatCvAUfKttLearuWrP9MDH5MBPbIqV92AaeXatLxBI9gBaebbnrfifHhDYfgasaacH8akY=wiFfYdH8Gipec8Eeeu0xXdbba9frFj0=OqFfea0dXdd9vqai=hGuQ8kuc9pgc9s8qqaq=dirpe0xb9q8qiLsFr0=vr0=vr0dc8meaabaqaciaacaGaaeqabaqabeGadaaakeaadaWdXbqaamaapehabaGaemizaqMaemOCaiNaemizaqMafmOCaiNbauaaaSqaaiabdggaHbqaaiabdkfasbqdcqGHRiI8aaWcbaGaemyyaegabaGaemOuaifaniabgUIiYdGcdaWcaaqaaiabigdaXaqaaiabdkhaYnaaCaaaleqabaGaemiBaWMaeyOeI0IaeGymaedaaaaakmaalaaabaGaeGymaedabaGafmOCaiNbauaadaahaaWcbeqaaiqbdYgaSzaafaGaeyOeI0IaeGymaedaaaaakmaacmqaeaqabeaadaWcaaqaaiqbdkhaYzaafaWaaWbaaSqabeaacqWGmbatdaahaaadbeqaaiabgEHiQaaaaaaakeaacqWGYbGCdaahaaWcbeqaaiabdYeamnaaCaaameqabaGaey4fIOcaaSGaey4kaSIaemiCaahaaaaakiabcUda7iqbdkhaYzaafaGaeyipaWJaemOCaihabaWaaSaaaeaacqWGYbGCdaahaaWcbeqaaiabdYeamnaaCaaameqabaGaey4fIOcaaaaaaOqaaiqbdkhaYzaafaWaaWbaaSqabeaacqWGmbatdaahaaadbeqaaiabgEHiQaaaliabgUcaRiabdchaWbaaaaGccqGG7aWocuWGYbGCgaqbaiabgwMiZkabdkhaYbaacaGL7bGaayzFaaGaeyypa0Zaa8qCaeaacqWGKbazcqWGYbGCaSqaaiabdggaHbqaaiabdkfasbqdcqGHRiI8aOWaamWaaeaadaWdXbqaaiabdsgaKjqbdkhaYzaafaaaleaacqWGHbqyaeaacqWGYbGCa0Gaey4kIipakmaalaaabaGafmOCaiNbauaadaahaaWcbeqaaiabdYeamnaaCaaameqabaGaey4fIOcaaSGaeyOeI0IafmiBaWMbauaacqGHRaWkcqaIXaqmaaaakeaacqWGYbGCdaahaaWcbeqaaiabdYeamnaaCaaameqabaGaey4fIOcaaSGaey4kaSIaemiCaaNaey4kaSIaemiBaWMaeyOeI0IaeGymaedaaaaakiabgUcaRmaapehabaGaemizaqMafmOCaiNbauaaaSqaaiabdkhaYbqaaiabdkfasbqdcqGHRiI8aOWaaSaaaeaacqWGYbGCdaahaaWcbeqaaiabdYeamnaaCaaameqabaGaey4fIOcaaSGaeyOeI0IaemiBaWMaey4kaSIaeGymaedaaaGcbaGafmOCaiNbauaadaahaaWcbeqaaiabdYeamnaaCaaameqabaGaey4fIOcaaSGaey4kaSIaemiCaaNaey4kaSIafmiBaWMbauaacqGHsislcqaIXaqmaaaaaaGccaGLBbGaayzxaaGaaCzcaiaaxMaadaqadaqaaiabikdaYiabiMda5aGaayjkaiaawMcaaaaa@AE3B@

Evaluating this integral is straightforward but tedious with the result:

∫aRdr∫ardr′r′L∗−l′+1rL∗+p+l−1=∫ardr{r′L∗−l′+2−aL∗−l′+2(L∗−l′+2)rL∗+p+l−1;(L∗−l′+2≠0ln⁡(ra)rL∗+p+l−1;(L*−l′+2)=0}={[R(−p−l′−l+4)−a(−p−l′−l+4)(−p−l′−l+4)(L∗−l′+2)−(R(−L∗−p−l+2)−a(−L*−p−l+2))aL∗−l′+2(L∗−l′+2)(−L∗−p−l+2)];(L∗−l′+2)≠0,(−p−l′−l+4)≠0,(−L∗−p−l+2)≠0[ln⁡(Ra)(L∗−l′+2)−(R(−L∗−p−l+2)−a(−L*−p−l+2))aL∗−l′+2(L∗−l′+2)(−L∗−p−l+2)];(L∗−l′+2)≠0,(−p−l′−l+4)=0,(−L∗−p−l+2)≠0[R(−p−l′−l+4)−a(−p−l′−l+4)(−p−l′−l+4)(−L∗−l′+2)−ln⁡(Ra)aL∗−l′+2(L∗−l′+2)];(L∗−l′+2)≠0,(−p−l′−l+4)≠0,(−L∗−p−l+2)=012ln⁡2(Ra);(L*−l′+2)=0,(2−L∗−p−l)=0R(2−L∗−p−1)((2−L∗−p−l)∗ln⁡(Ra)−1)(2−L∗−p−l)2+a(2−L∗−p−l)(2−L∗−p−l)2;(L∗−l′+2)=0,(2−L∗−p−l)≠0}     (30)
MathType@MTEF@5@5@+=feaafiart1ev1aaatCvAUfKttLearuWrP9MDH5MBPbIqV92AaeXatLxBI9gBaebbnrfifHhDYfgasaacH8akY=wiFfYdH8Gipec8Eeeu0xXdbba9frFj0=OqFfea0dXdd9vqai=hGuQ8kuc9pgc9s8qqaq=dirpe0xb9q8qiLsFr0=vr0=vr0dc8meaabaqaciaacaGaaeqabaqabeGadaaakeaafaqaaeGabaaabaWaa8qCaeaacqWGKbazcqWGYbGCaSqaaiabdggaHbqaaiabdkfasbqdcqGHRiI8aOWaa8qCaeaacqWGKbazcuWGYbGCgaqbaaWcbaGaemyyaegabaGaemOCaihaniabgUIiYdGcdaWcaaqaaiqbdkhaYzaafaWaaWbaaSqabeaacqWGmbatdaahaaadbeqaaiabgEHiQaaaliabgkHiTiqbdYgaSzaafaGaey4kaSIaeGymaedaaaGcbaGaemOCai3aaWbaaSqabeaacqWGmbatdaahaaadbeqaaiabgEHiQaaaliabgUcaRiabdchaWjabgUcaRiabdYgaSjabgkHiTiabigdaXaaaaaGccqGH9aqpdaWdXbqaaiabdsgaKjabdkhaYbWcbaGaemyyaegabaGaemOCaihaniabgUIiYdGcdaGadeqaauaabeqaceaaaeaadaWcaaqaaiqbdkhaYzaafaWaaWbaaSqabeaacqWGmbatdaahaaadbeqaaiabgEHiQaaaliabgkHiTiqbdYgaSzaafaGaey4kaSIaeGOmaidaaOGaeyOeI0Iaemyyae2aaWbaaSqabeaacqWGmbatdaahaaadbeqaaiabgEHiQaaaliabgkHiTiqbdYgaSzaafaGaey4kaSIaeGOmaidaaaGcbaGaeiikaGIaemitaW0aaWbaaSqabeaacqGHxiIkaaGccqGHsislcuWGSbaBgaqbaiabgUcaRiabikdaYiabcMcaPiabdkhaYnaaCaaaleqabaGaemitaW0aaWbaaWqabeaacqGHxiIkaaWccqGHRaWkcqWGWbaCcqGHRaWkcqWGSbaBcqGHsislcqaIXaqmaaaaaOGaei4oaSJaeiikaGIaemitaW0aaWbaaSqabeaacqGHxiIkaaGccqGHsislcuWGSbaBgaqbaiabgUcaRiabikdaYiabgcMi5kabicdaWaqaamaalaaabaGagiiBaWMaeiOBa42aaeWaaeaadaWcaaqaaiabdkhaYbqaaiabdggaHbaaaiaawIcacaGLPaaaaeaacqWGYbGCdaahaaWcbeqaaiabdYeamnaaCaaameqabaGaey4fIOcaaSGaey4kaSIaemiCaaNaey4kaSIaemiBaWMaeyOeI0IaeGymaedaaaaakiabcUda7iabcIcaOiabdYeamnaaCaaaleqabaGaeiOkaOcaaOGaeyOeI0IafmiBaWMbauaacqGHRaWkcqaIYaGmcqGGPaqkcqGH9aqpcqaIWaamaaaacaGL7bGaayzFaaaabaGaeyypa0ZaaiWabeaafaqabeqbbaaaaeaadaWadaqaamaalaaabaGaemOuai1aaWbaaSqabeaacqGGOaakcqGHsislcqWGWbaCcqGHsislcuWGSbaBgaqbaiabgkHiTiabdYgaSjabgUcaRiabisda0iabcMcaPaaakiabgkHiTiabdggaHnaaCaaaleqabaGaeiikaGIaeyOeI0IaemiCaaNaeyOeI0IafmiBaWMbauaacqGHsislcqWGSbaBcqGHRaWkcqaI0aancqGGPaqkaaaakeaacqGGOaakcqGHsislcqWGWbaCcqGHsislcuWGSbaBgaqbaiabgkHiTiabdYgaSjabgUcaRiabisda0iabcMcaPiabcIcaOiabdYeamnaaCaaaleqabaGaey4fIOcaaOGaeyOeI0IafmiBaWMbauaacqGHRaWkcqaIYaGmcqGGPaqkaaGaeyOeI0YaaSaaaeaadaqadaqaaiabdkfasnaaCaaaleqabaGaeiikaGIaeyOeI0IaemitaW0aaWbaaWqabeaacqGHxiIkaaWccqGHsislcqWGWbaCcqGHsislcqWGSbaBcqGHRaWkcqaIYaGmcqGGPaqkaaGccqGHsislcqWGHbqydaahaaWcbeqaaiabcIcaOiabgkHiTiabdYeamnaaCaaameqabaGaeiOkaOcaaSGaeyOeI0IaemiCaaNaeyOeI0IaemiBaWMaey4kaSIaeGOmaiJaeiykaKcaaaGccaGLOaGaayzkaaGaemyyae2aaWbaaSqabeaacqWGmbatdaahaaadbeqaaiabgEHiQaaaliabgkHiTiqbdYgaSzaafaGaey4kaSIaeGOmaidaaaGcbaGaeiikaGIaemitaW0aaWbaaSqabeaacqGHxiIkaaGccqGHsislcuWGSbaBgaqbaiabgUcaRiabikdaYiabcMcaPiabcIcaOiabgkHiTiabdYeamnaaCaaaleqabaGaey4fIOcaaOGaeyOeI0IaemiCaaNaeyOeI0IaemiBaWMaey4kaSIaeGOmaiJaeiykaKcaaaGaay5waiaaw2faaiabcUda7iabcIcaOiabdYeamnaaCaaaleqabaGaey4fIOcaaOGaeyOeI0IafmiBaWMbauaacqGHRaWkcqaIYaGmcqGGPaqkcqGHGjsUcqaIWaamcqGGSaalcqGGOaakcqGHsislcqWGWbaCcqGHsislcuWGSbaBgaqbaiabgkHiTiabdYgaSjabgUcaRiabisda0iabcMcaPiabgcMi5kabicdaWiabcYcaSiabcIcaOiabgkHiTiabdYeamnaaCaaaleqabaGaey4fIOcaaOGaeyOeI0IaemiCaaNaeyOeI0IaemiBaWMaey4kaSIaeGOmaiJaeiykaKIaeyiyIKRaeGimaadabaWaamWaaeaadaWcaaqaaiGbcYgaSjabc6gaUnaabmaabaWaaSaaaeaacqWGsbGuaeaacqWGHbqyaaaacaGLOaGaayzkaaaabaGaeiikaGIaemitaW0aaWbaaSqabeaacqGHxiIkaaGccqGHsislcuWGSbaBgaqbaiabgUcaRiabikdaYiabcMcaPaaacqGHsisldaWcaaqaamaabmaabaGaemOuai1aaWbaaSqabeaacqGGOaakcqGHsislcqWGmbatdaahaaadbeqaaiabgEHiQaaaliabgkHiTiabdchaWjabgkHiTiabdYgaSjabgUcaRiabikdaYiabcMcaPaaakiabgkHiTiabdggaHnaaCaaaleqabaGaeiikaGIaeyOeI0IaemitaW0aaWbaaWqabeaacqGGQaGkaaWccqGHsislcqWGWbaCcqGHsislcqWGSbaBcqGHRaWkcqaIYaGmcqGGPaqkaaaakiaawIcacaGLPaaacqWGHbqydaahaaWcbeqaaiabdYeamnaaCaaameqabaGaey4fIOcaaSGaeyOeI0IafmiBaWMbauaacqGHRaWkcqaIYaGmaaaakeaacqGGOaakcqWGmbatdaahaaWcbeqaaiabgEHiQaaakiabgkHiTiqbdYgaSzaafaGaey4kaSIaeGOmaiJaeiykaKIaeiikaGIaeyOeI0IaemitaW0aaWbaaSqabeaacqGHxiIkaaGccqGHsislcqWGWbaCcqGHsislcqWGSbaBcqGHRaWkcqaIYaGmcqGGPaqkaaaacaGLBbGaayzxaaGaei4oaSJaeiikaGIaemitaW0aaWbaaSqabeaacqGHxiIkaaGccqGHsislcuWGSbaBgaqbaiabgUcaRiabikdaYiabcMcaPiabgcMi5kabicdaWiabcYcaSiabcIcaOiabgkHiTiabdchaWjabgkHiTiqbdYgaSzaafaGaeyOeI0IaemiBaWMaey4kaSIaeGinaqJaeiykaKIaeyypa0JaeGimaaJaeiilaWIaeiikaGIaeyOeI0IaemitaW0aaWbaaSqabeaacqGHxiIkaaGccqGHsislcqWGWbaCcqGHsislcqWGSbaBcqGHRaWkcqaIYaGmcqGGPaqkcqGHGjsUcqaIWaamaeaadaWadaqaamaalaaabaGaemOuai1aaWbaaSqabeaacqGGOaakcqGHsislcqWGWbaCcqGHsislcuWGSbaBgaqbaiabgkHiTiabdYgaSjabgUcaRiabisda0iabcMcaPaaakiabgkHiTiabdggaHnaaCaaaleqabaGaeiikaGIaeyOeI0IaemiCaaNaeyOeI0IafmiBaWMbauaacqGHsislcqWGSbaBcqGHRaWkcqaI0aancqGGPaqkaaaakeaacqGGOaakcqGHsislcqWGWbaCcqGHsislcuWGSbaBgaqbaiabgkHiTiabdYgaSjabgUcaRiabisda0iabcMcaPiabcIcaOiabgkHiTiabdYeamnaaCaaaleqabaGaey4fIOcaaOGaeyOeI0IafmiBaWMbauaacqGHRaWkcqaIYaGmcqGGPaqkaaGaeyOeI0YaaSaaaeaacyGGSbaBcqGGUbGBdaqadaqaamaalaaabaGaemOuaifabaGaemyyaegaaaGaayjkaiaawMcaaiabdggaHnaaCaaaleqabaGaemitaW0aaWbaaWqabeaacqGHxiIkaaWccqGHsislcuWGSbaBgaqbaiabgUcaRiabikdaYaaaaOqaaiabcIcaOiabdYeamnaaCaaaleqabaGaey4fIOcaaOGaeyOeI0IafmiBaWMbauaacqGHRaWkcqaIYaGmcqGGPaqkaaaacaGLBbGaayzxaaGaei4oaSJaeiikaGIaemitaW0aaWbaaSqabeaacqGHxiIkaaGccqGHsislcuWGSbaBgaqbaiabgUcaRiabikdaYiabcMcaPiabgcMi5kabicdaWiabcYcaSiabcIcaOiabgkHiTiabdchaWjabgkHiTiqbdYgaSzaafaGaeyOeI0IaemiBaWMaey4kaSIaeGinaqJaeiykaKIaeyiyIKRaeGimaaJaeiilaWIaeiikaGIaeyOeI0IaemitaW0aaWbaaSqabeaacqGHxiIkaaGccqGHsislcqWGWbaCcqGHsislcqWGSbaBcqGHRaWkcqaIYaGmcqGGPaqkcqGH9aqpcqaIWaamaeaadaWcaaqaaiabigdaXaqaaiabikdaYaaacyGGSbaBcqGGUbGBdaahaaWcbeqaaiabikdaYaaakmaabmaabaWaaSaaaeaacqWGsbGuaeaacqWGHbqyaaaacaGLOaGaayzkaaGaei4oaSJaeiikaGIaemitaW0aaWbaaSqabeaacqGGQaGkaaGccqGHsislcuWGSbaBgaqbaiabgUcaRiabikdaYiabcMcaPiabg2da9iabicdaWiabcYcaSiabcIcaOiabikdaYiabgkHiTiabdYeamnaaCaaaleqabaGaey4fIOcaaOGaeyOeI0IaemiCaaNaeyOeI0IaemiBaWMaeiykaKIaeyypa0JaeGimaadabaWaaSaaaeaacqWGsbGudaahaaWcbeqaaiabcIcaOiabikdaYiabgkHiTiabdYeamnaaCaaameqabaGaey4fIOcaaSGaeyOeI0IaemiCaaNaeyOeI0IaeGymaeJaeiykaKcaaOWaaeWaaeaacqGGOaakcqaIYaGmcqGHsislcqWGmbatdaahaaWcbeqaaiabgEHiQaaakiabgkHiTiabdchaWjabgkHiTiabdYgaSjabcMcaPiabgEHiQiGbcYgaSjabc6gaUnaabmaabaWaaSaaaeaacqWGsbGuaeaacqWGHbqyaaaacaGLOaGaayzkaaGaeyOeI0IaeGymaedacaGLOaGaayzkaaaabaGaeiikaGIaeGOmaiJaeyOeI0IaemitaW0aaWbaaSqabeaacqGHxiIkaaGccqGHsislcqWGWbaCcqGHsislcqWGSbaBcqGGPaqkdaahaaWcbeqaaiabikdaYaaaaaGccqGHRaWkdaWcaaqaaiabdggaHnaaCaaaleqabaGaeiikaGIaeGOmaiJaeyOeI0IaemitaW0aaWbaaWqabeaacqGHxiIkaaWccqGHsislcqWGWbaCcqGHsislcqWGSbaBcqGGPaqkaaaakeaacqGGOaakcqaIYaGmcqGHsislcqWGmbatdaahaaWcbeqaaiabgEHiQaaakiabgkHiTiabdchaWjabgkHiTiabdYgaSjabcMcaPmaaCaaaleqabaGaeGOmaidaaaaakiabcUda7iabcIcaOiabdYeamnaaCaaaleqabaGaey4fIOcaaOGaeyOeI0IafmiBaWMbauaacqGHRaWkcqaIYaGmcqGGPaqkcqGH9aqpcqaIWaamcqGGSaalcqGGOaakcqaIYaGmcqGHsislcqWGmbatdaahaaWcbeqaaiabgEHiQaaakiabgkHiTiabdchaWjabgkHiTiabdYgaSjabcMcaPiabgcMi5kabicdaWaaaaiaawUhacaGL9baaaaGaaCzcaiaaxMaadaqadaqaaiabiodaZiabicdaWaGaayjkaiaawMcaaaaa@9D50@

And analogously

∫aRdr∫ardr′r′−L∗−p−l′+1rL∗−l+1=∫ardr{rL∗−l+1R2−(L∗+p+l′)−r2−(L∗+p+l′)(2−L∗−p−l′);(2−L∗−p−l′)≠0rL∗−l+1ln⁡(Rr);(2−L*−p−l′)=0}={[(R(L∗−l+2)−a(L∗−l+2))R2−(L∗−p+l′)(2−L∗−p−l′)(L∗−l+2)−R(−p−l−l′+4)−a(−p−l−l′+4)(−p−l−l′+4)(2−L∗−p−l′)];(2−L∗−p−l′)≠0,(L∗−l+2)≠0,(−p−l−l′+4)≠0[R(L∗−l+2)(L∗−l+2)2−a(L∗−l+2)((L∗−l+2)ln⁡(Ra)+1)(L∗−l+2)2];(2−L∗−p−l′)=0,,(L∗−l+2)≠0[12ln⁡2(Ra)];(2−L∗−p−l′)=0,(L∗−l+2)=0[(R(L∗−l+2)−a(L∗−l+2))R2−(L∗+p+l′)(2−L∗−p−l′)(L∗−l+2)−ln⁡(Ra)(2−L∗−p−l′)];(2−L∗−p−l′)≠0,(L∗−l+2)≠0,(−p−l−l′+4)≠0[ln⁡(Ra)R2−(L∗+p+l′)(2−L∗−p−l′)−R(−p−l−l′+4)−a(−p−l−l′+4)(−p−l−l′+4)(2−L∗−p−l′)];(2−L∗−p−l′)≠0,(L∗−l+2)=0,(−p−l−l′+4)≠0}     (31)
MathType@MTEF@5@5@+=feaafiart1ev1aaatCvAUfKttLearuWrP9MDH5MBPbIqV92AaeXatLxBI9gBaebbnrfifHhDYfgasaacH8akY=wiFfYdH8Gipec8Eeeu0xXdbba9frFj0=OqFfea0dXdd9vqai=hGuQ8kuc9pgc9s8qqaq=dirpe0xb9q8qiLsFr0=vr0=vr0dc8meaabaqaciaacaGaaeqabaqabeGadaaakeaafaqaaeGabaaabaWaa8qCaeaacqWGKbazcqWGYbGCaSqaaiabdggaHbqaaiabdkfasbqdcqGHRiI8aOWaa8qCaeaacqWGKbazcuWGYbGCgaqbaaWcbaGaemyyaegabaGaemOCaihaniabgUIiYdGccuWGYbGCgaqbamaaCaaaleqabaGaeyOeI0IaemitaW0aaWbaaWqabeaacqGHxiIkaaWccqGHsislcqWGWbaCcqGHsislcuWGSbaBgaqbaiabgUcaRiabigdaXaaakiabdkhaYnaaCaaaleqabaGaemitaW0aaWbaaWqabeaacqGHxiIkaaWccqGHsislcqWGSbaBcqGHRaWkcqaIXaqmaaGccqGH9aqpdaWdXbqaaiabdsgaKjabdkhaYbWcbaGaemyyaegabaGaemOCaihaniabgUIiYdGcdaGadeqaauaabeqaceaaaeaacqWGYbGCdaahaaWcbeqaaiabdYeamnaaCaaameqabaGaey4fIOcaaSGaeyOeI0IaemiBaWMaey4kaSIaeGymaedaaOWaaSaaaeaacqWGsbGudaahaaWcbeqaaiabikdaYiabgkHiTiabcIcaOiabdYeamnaaCaaameqabaGaey4fIOcaaSGaey4kaSIaemiCaaNaey4kaSIafmiBaWMbauaacqGGPaqkaaGccqGHsislcqWGYbGCdaahaaWcbeqaaiabikdaYiabgkHiTiabcIcaOiabdYeamnaaCaaameqabaGaey4fIOcaaSGaey4kaSIaemiCaaNaey4kaSIafmiBaWMbauaacqGGPaqkaaaakeaacqGGOaakcqaIYaGmcqGHsislcqWGmbatdaahaaWcbeqaaiabgEHiQaaakiabgkHiTiabdchaWjabgkHiTiqbdYgaSzaafaGaeiykaKcaaiabcUda7iabcIcaOiabikdaYiabgkHiTiabdYeamnaaCaaaleqabaGaey4fIOcaaOGaeyOeI0IaemiCaaNaeyOeI0IafmiBaWMbauaacqGGPaqkcqGHGjsUcqaIWaamaeaacqWGYbGCdaahaaWcbeqaaiabdYeamnaaCaaameqabaGaey4fIOcaaSGaeyOeI0IaemiBaWMaey4kaSIaeGymaedaaOGagiiBaWMaeiOBa42aaeWaaeaadaWcaaqaaiabdkfasbqaaiabdkhaYbaaaiaawIcacaGLPaaacqGG7aWocqGGOaakcqaIYaGmcqGHsislcqWGmbatdaahaaWcbeqaaiabcQcaQaaakiabgkHiTiabdchaWjabgkHiTiqbdYgaSzaafaGaeiykaKIaeyypa0JaeGimaadaaaGaay5Eaiaaw2haaaqaaiabg2da9maacmqabaqbaeqabuqaaaaabaWaamWaaeaadaWcaaqaamaabmaabaGaemOuai1aaWbaaSqabeaacqGGOaakcqWGmbatdaahaaadbeqaaiabgEHiQaaaliabgkHiTiabdYgaSjabgUcaRiabikdaYiabcMcaPaaakiabgkHiTiabdggaHnaaCaaaleqabaGaeiikaGIaemitaW0aaWbaaWqabeaacqGHxiIkaaWccqGHsislcqWGSbaBcqGHRaWkcqaIYaGmcqGGPaqkaaaakiaawIcacaGLPaaacqWGsbGudaahaaWcbeqaaiabikdaYiabgkHiTiabcIcaOiabdYeamnaaCaaameqabaGaey4fIOcaaSGaeyOeI0IaemiCaaNaey4kaSIafmiBaWMbauaacqGGPaqkaaaakeaacqGGOaakcqaIYaGmcqGHsislcqWGmbatdaahaaWcbeqaaiabgEHiQaaakiabgkHiTiabdchaWjabgkHiTiqbdYgaSzaafaGaeiykaKIaeiikaGIaemitaW0aaWbaaSqabeaacqGHxiIkaaGccqGHsislcqWGSbaBcqGHRaWkcqaIYaGmcqGGPaqkaaGaeyOeI0YaaSaaaeaacqWGsbGudaahaaWcbeqaaiabcIcaOiabgkHiTiabdchaWjabgkHiTiabdYgaSjabgkHiTiqbdYgaSzaafaGaey4kaSIaeGinaqJaeiykaKcaaOGaeyOeI0Iaemyyae2aaWbaaSqabeaacqGGOaakcqGHsislcqWGWbaCcqGHsislcqWGSbaBcqGHsislcuWGSbaBgaqbaiabgUcaRiabisda0iabcMcaPaaaaOqaaiabcIcaOiabgkHiTiabdchaWjabgkHiTiabdYgaSjabgkHiTiqbdYgaSzaafaGaey4kaSIaeGinaqJaeiykaKIaeiikaGIaeGOmaiJaeyOeI0IaemitaW0aaWbaaSqabeaacqGHxiIkaaGccqGHsislcqWGWbaCcqGHsislcuWGSbaBgaqbaiabcMcaPaaaaiaawUfacaGLDbaacqGG7aWocqGGOaakcqaIYaGmcqGHsislcqWGmbatdaahaaWcbeqaaiabgEHiQaaakiabgkHiTiabdchaWjabgkHiTiqbdYgaSzaafaGaeiykaKIaeyiyIKRaeGimaaJaeiilaWIaeiikaGIaemitaW0aaWbaaSqabeaacqGHxiIkaaGccqGHsislcqWGSbaBcqGHRaWkcqaIYaGmcqGGPaqkcqGHGjsUcqaIWaamcqGGSaalcqGGOaakcqGHsislcqWGWbaCcqGHsislcqWGSbaBcqGHsislcuWGSbaBgaqbaiabgUcaRiabisda0iabcMcaPiabgcMi5kabicdaWaqaamaadmaabaWaaSaaaeaacqWGsbGudaahaaWcbeqaaiabcIcaOiabdYeamnaaCaaameqabaGaey4fIOcaaSGaeyOeI0IaemiBaWMaey4kaSIaeGOmaiJaeiykaKcaaaGcbaGaeiikaGIaemitaW0aaWbaaSqabeaacqGHxiIkaaGccqGHsislcqWGSbaBcqGHRaWkcqaIYaGmcqGGPaqkdaahaaWcbeqaaiabikdaYaaaaaGccqGHsisldaWcaaqaaiabdggaHnaaCaaaleqabaGaeiikaGIaemitaW0aaWbaaWqabeaacqGHxiIkaaWccqGHsislcqWGSbaBcqGHRaWkcqaIYaGmcqGGPaqkaaGcdaqadaqaaiabcIcaOiabdYeamnaaCaaaleqabaGaey4fIOcaaOGaeyOeI0IaemiBaWMaey4kaSIaeGOmaiJaeiykaKIagiiBaWMaeiOBa42aaeWaaeaadaWcaaqaaiabdkfasbqaaiabdggaHbaaaiaawIcacaGLPaaacqGHRaWkcqaIXaqmaiaawIcacaGLPaaaaeaacqGGOaakcqWGmbatdaahaaWcbeqaaiabgEHiQaaakiabgkHiTiabdYgaSjabgUcaRiabikdaYiabcMcaPmaaCaaaleqabaGaeGOmaidaaaaaaOGaay5waiaaw2faaiabcUda7iabcIcaOiabikdaYiabgkHiTiabdYeamnaaCaaaleqabaGaey4fIOcaaOGaeyOeI0IaemiCaaNaeyOeI0IafmiBaWMbauaacqGGPaqkcqGH9aqpcqaIWaamcqGGSaalcqGGSaalcqGGOaakcqWGmbatdaahaaWcbeqaaiabgEHiQaaakiabgkHiTiabdYgaSjabgUcaRiabikdaYiabcMcaPiabgcMi5kabicdaWaqaamaadmaabaWaaSaaaeaacqaIXaqmaeaacqaIYaGmaaGagiiBaWMaeiOBa42aaWbaaSqabeaacqaIYaGmaaGcdaqadaqaamaalaaabaGaemOuaifabaGaemyyaegaaaGaayjkaiaawMcaaaGaay5waiaaw2faaiabcUda7iabcIcaOiabikdaYiabgkHiTiabdYeamnaaCaaaleqabaGaey4fIOcaaOGaeyOeI0IaemiCaaNaeyOeI0IafmiBaWMbauaacqGGPaqkcqGH9aqpcqaIWaamcqGGSaalcqGGOaakcqWGmbatdaahaaWcbeqaaiabgEHiQaaakiabgkHiTiabdYgaSjabgUcaRiabikdaYiabcMcaPiabg2da9iabicdaWaqaamaadmaabaWaaSaaaeaadaqadaqaaiabdkfasnaaCaaaleqabaGaeiikaGIaemitaW0aaWbaaWqabeaacqGHxiIkaaWccqGHsislcqWGSbaBcqGHRaWkcqaIYaGmcqGGPaqkaaGccqGHsislcqWGHbqydaahaaWcbeqaaiabcIcaOiabdYeamnaaCaaameqabaGaey4fIOcaaSGaeyOeI0IaemiBaWMaey4kaSIaeGOmaiJaeiykaKcaaaGccaGLOaGaayzkaaGaemOuai1aaWbaaSqabeaacqaIYaGmcqGHsislcqGGOaakcqWGmbatdaahaaadbeqaaiabgEHiQaaaliabgUcaRiabdchaWjabgUcaRiqbdYgaSzaafaGaeiykaKcaaaGcbaGaeiikaGIaeGOmaiJaeyOeI0IaemitaW0aaWbaaSqabeaacqGHxiIkaaGccqGHsislcqWGWbaCcqGHsislcuWGSbaBgaqbaiabcMcaPiabcIcaOiabdYeamnaaCaaaleqabaGaey4fIOcaaOGaeyOeI0IaemiBaWMaey4kaSIaeGOmaiJaeiykaKcaaiabgkHiTmaalaaabaGagiiBaWMaeiOBa42aaeWaaeaadaWcaaqaaiabdkfasbqaaiabdggaHbaaaiaawIcacaGLPaaaaeaacqGGOaakcqaIYaGmcqGHsislcqWGmbatdaahaaWcbeqaaiabgEHiQaaakiabgkHiTiabdchaWjabgkHiTiqbdYgaSzaafaGaeiykaKcaaaGaay5waiaaw2faaiabcUda7iabcIcaOiabikdaYiabgkHiTiabdYeamnaaCaaaleqabaGaey4fIOcaaOGaeyOeI0IaemiCaaNaeyOeI0IafmiBaWMbauaacqGGPaqkcqGHGjsUcqaIWaamcqGGSaalcqGGOaakcqWGmbatdaahaaWcbeqaaiabgEHiQaaakiabgkHiTiabdYgaSjabgUcaRiabikdaYiabcMcaPiabgcMi5kabicdaWiabcYcaSiabcIcaOiabgkHiTiabdchaWjabgkHiTiabdYgaSjabgkHiTiqbdYgaSzaafaGaey4kaSIaeGinaqJaeiykaKIaeyiyIKRaeGimaadabaWaamWaaeaadaWcaaqaaiGbcYgaSjabc6gaUnaabmaabaWaaSaaaeaacqWGsbGuaeaacqWGHbqyaaaacaGLOaGaayzkaaGaemOuai1aaWbaaSqabeaacqaIYaGmcqGHsislcqGGOaakcqWGmbatdaahaaadbeqaaiabgEHiQaaaliabgUcaRiabdchaWjabgUcaRiqbdYgaSzaafaGaeiykaKcaaaGcbaGaeiikaGIaeGOmaiJaeyOeI0IaemitaW0aaWbaaSqabeaacqGHxiIkaaGccqGHsislcqWGWbaCcqGHsislcuWGSbaBgaqbaiabcMcaPaaacqGHsisldaWcaaqaaiabdkfasnaaCaaaleqabaGaeiikaGIaeyOeI0IaemiCaaNaeyOeI0IaemiBaWMaeyOeI0IafmiBaWMbauaacqGHRaWkcqaI0aancqGGPaqkaaGccqGHsislcqWGHbqydaahaaWcbeqaaiabcIcaOiabgkHiTiabdchaWjabgkHiTiabdYgaSjabgkHiTiqbdYgaSzaafaGaey4kaSIaeGinaqJaeiykaKcaaaGcbaGaeiikaGIaeyOeI0IaemiCaaNaeyOeI0IaemiBaWMaeyOeI0IafmiBaWMbauaacqGHRaWkcqaI0aancqGGPaqkcqGGOaakcqaIYaGmcqGHsislcqWGmbatdaahaaWcbeqaaiabgEHiQaaakiabgkHiTiabdchaWjabgkHiTiqbdYgaSzaafaGaeiykaKcaaaGaay5waiaaw2faaiabcUda7iabcIcaOiabikdaYiabgkHiTiabdYeamnaaCaaaleqabaGaey4fIOcaaOGaeyOeI0IaemiCaaNaeyOeI0IafmiBaWMbauaacqGGPaqkcqGHGjsUcqaIWaamcqGGSaalcqGGOaakcqWGmbatdaahaaWcbeqaaiabgEHiQaaakiabgkHiTiabdYgaSjabgUcaRiabikdaYiabcMcaPiabg2da9iabicdaWiabcYcaSiabcIcaOiabgkHiTiabdchaWjabgkHiTiabdYgaSjabgkHiTiqbdYgaSzaafaGaey4kaSIaeGinaqJaeiykaKIaeyiyIKRaeGimaadaaaGaay5Eaiaaw2haaaaacaWLjaGaaCzcamaabmaabaGaeG4mamJaeGymaedacaGLOaGaayzkaaaaaa@A567@

It is reasonable to assume that all sources are either dipolar or quadrupolar in order to simplify the calculations. Because of this, it is possible to set l = l'. The above integrals suggest that the responses for large R are convergent if:

4-*p*-2*l *< 0     (32)

This implies that, for dipolar sources for p > 2 and for quadrupolar sources p > 0 guarantee convergent responses for large R or in other words that the recorded signal is dominated by the contribution of nearby neural structures. Since p must be less than 3 or else the integrals do not converge, this demonstrates that, although there may be specific exceptions, in general with dipolar sources and power law correlations, the autocovariance function is a strong function R. It should be noted that this argument provides only a maximum bound on the autocovariance function and hence does not prove that the autocovariance must be extremely sensitive to the value of R outside the range of parameters discussed above. It is possible, however, in one special case to demonstrate the sensitivity to the value of R outside the above restrictions on p. Consider the specific case p = 1, l, l' = 1 where in addition:

ℚlm(x→1)=ℚlm0hp(x→1,τ)=hp0(τ)G(1,1,1,L1,|x→1|,|x→1′|,τ)=hp0(τ)9∫dΩ1dΩ1′∑m=−ll∑m′=−l′l′∑M1=−L1L1ℚ1m0ℚ1m′0Y1m(θ1,φ1)Y1m′(θ′1,φ′1)[12L1+1YLiMi∗(θ1′,φ1′)YLiMi(θ1,φ1)]     (33)
 MathType@MTEF@5@5@+=feaafiart1ev1aaatCvAUfKttLearuWrP9MDH5MBPbIqV92AaeXatLxBI9gBaebbnrfifHhDYfgasaacH8akY=wiFfYdH8Gipec8Eeeu0xXdbba9frFj0=OqFfea0dXdd9vqai=hGuQ8kuc9pgc9s8qqaq=dirpe0xb9q8qiLsFr0=vr0=vr0dc8meaabaqaciaacaGaaeqabaqabeGadaaakqaabeqaaiablQriKoaaBaaaleaacqWGSbaBcqWGTbqBaeqaaOGaeiikaGIafmiEaGNbaSaadaWgaaWcbaGaeGymaedabeaakiabcMcaPiabg2da9iablQriKoaaDaaaleaacqWGSbaBcqWGTbqBaeaacqaIWaamaaaakeaacqWGObaAdaWgaaWcbaGaemiCaahabeaakiabcIcaOiqbdIha4zaalaWaaSbaaSqaaiabigdaXaqabaGccqGGSaaliiGacqWFepaDcqGGPaqkcqGH9aqpcqWGObaAdaqhaaWcbaGaemiCaahabaGaeGimaadaaOGaeiikaGIae8hXdqNaeiykaKcabaGaem4raCKaeiikaGIaeGymaeJaeiilaWIaeGymaeJaeiilaWIaeGymaeJaeiilaWIaemitaW0aaSbaaSqaaiabigdaXaqabaGccqGGSaalcqGG8baFcuWG4baEgaWcamaaBaaaleaacqaIXaqmaeqaaOGaeiiFaWNaeiilaWIaeiiFaWNafmiEaGNbaSaadaWgaaWcbaGaeGymaedabeaaiiaakiab+jdiIkabcYha8jabcYcaSiab=r8a0jabcMcaPiabg2da9aqaamaalaaabaGaemiAaG2aa0baaSqaaiabdchaWbqaaiabicdaWaaakiabcIcaOiab=r8a0jabcMcaPaqaaiabiMda5aaadaWdbaqaaiabdsgaKjabgM6axnaaBaaaleaacqaIXaqmaeqaaOGaemizaqMaeyyQdC1aaSbaaSqaaiabigdaXaqabaGccqGFYaIOdaaeWbqaamaaqahabaWaaabCaeaacqWIAecPdaqhaaWcbaGaeGymaeJaemyBa0gabaGaeGimaadaaOGaeSOgHq6aa0baaSqaaiabigdaXiqbd2gaTzaafaaabaGaeGimaadaaaqaaiabd2eannaaBaaameaacqaIXaqmaeqaaSGaeyypa0JaeyOeI0IaemitaW0aaSbaaWqaaiabigdaXaqabaaaleaacqWGmbatdaWgaaadbaGaeGymaedabeaaa0GaeyyeIuoaaSqaaiqbd2gaTzaafaGaeyypa0JaeyOeI0IafmiBaWMbauaaaeaacuWGSbaBgaqbaaqdcqGHris5aaWcbaGaemyBa0Maeyypa0JaeyOeI0IaemiBaWgabaGaemiBaWganiabggHiLdaaleqabeqdcqGHRiI8aOGaemywaK1aaSbaaSqaaiabigdaXiabd2gaTbqabaGccqGGOaakcqWF4oqCdaWgaaWcbaGaeGymaedabeaakiabcYcaSiab=z8aMnaaBaaaleaacqaIXaqmaeqaaOGaeiykaKIaemywaK1aaSbaaSqaaiabigdaXiqbd2gaTzaafaaabeaakiabcIcaOiqb=H7aXzaafaWaaSbaaSqaaGqaaiab9fdaXaqabaGccqGGSaalcuWFgpGzgaqbamaaBaaaleaacqqFXaqmaeqaaOGaeiykaKYaamWaaeaadaWcaaqaaiabigdaXaqaaiabikdaYiabdYeamnaaBaaaleaacqaIXaqmaeqaaOGaey4kaSIaeGymaedaaiabdMfaznaaDaaaleaacqWGmbatdaWgaaadbaGaemyAaKgabeaaliabd2eannaaBaaameaacqWGPbqAaeqaaaWcbaGaey4fIOcaaOGaeiikaGIae8hUde3aaSbaaSqaaiab9fdaXaqabaGccqGFYaIOcqGGSaalcqWFgpGzdaWgaaWcbaGae0xmaedabeaakiab+jdiIkabcMcaPiabdMfaznaaBaaaleaacqWGmbatdaWgaaadbaGaemyAaKgabeaaliabd2eannaaBaaameaacqWGPbqAaeqaaaWcbeaakiabcIcaOiab=H7aXnaaBaaaleaacqaIXaqmaeqaaOGaeiilaWIae8NXdy2aaSbaaSqaaiabigdaXaqabaGccqGGPaqkaiaawUfacaGLDbaacaWLjaGaaCzcamaabmaabaGaeG4mamJaeG4mamdacaGLOaGaayzkaaaaaaa@EB86@

Orthogonality of the spherical harmonics implies:

G(1,1,1,L1,|x→1||x→1′|,τ)=h10(τ)27δL11∑M1=−L1L1ℚ1−M10ℚ1M10(−1)m     (34)
 MathType@MTEF@5@5@+=feaafiart1ev1aaatCvAUfKttLearuWrP9MDH5MBPbIqV92AaeXatLxBI9gBaebbnrfifHhDYfgasaacH8akY=wiFfYdH8Gipec8Eeeu0xXdbba9frFj0=OqFfea0dXdd9vqai=hGuQ8kuc9pgc9s8qqaq=dirpe0xb9q8qiLsFr0=vr0=vr0dc8meaabaqaciaacaGaaeqabaqabeGadaaakeaacqWGhbWrdaqadaqaaiabigdaXiabcYcaSiabigdaXiabcYcaSiabigdaXiabcYcaSiabdYeamnaaBaaaleaacqaIXaqmaeqaaOGaeiilaWYaaqWaaeaacuWG4baEgaWcamaaBaaaleaacqaIXaqmaeqaaaGccaGLhWUaayjcSdWaaqWaaeaacuWG4baEgaWcamaaBaaaleaacqaIXaqmaeqaaGGaaOGae8NmGikacaGLhWUaayjcSdGaeiilaWccciGae4hXdqhacaGLOaGaayzkaaGaeyypa0ZaaSaaaeaacqWGObaAdaqhaaWcbaGaeGymaedabaGaeGimaadaaOGaeiikaGIae4hXdqNaeiykaKcabaGaeGOmaiJaeG4naCdaaiab+r7aKnaaBaaaleaacqWGmbatdaWgaaadbaGaeGymaedabeaaliabigdaXaqabaGcdaaeWbqaaiablQriKoaaDaaaleaacqaIXaqmcqGHsislcqWGnbqtdaWgaaadbaGaeGymaedabeaaaSqaaiabicdaWaaakiablQriKoaaDaaaleaacqaIXaqmcqWGnbqtdaWgaaadbaGaeGymaedabeaaaSqaaiabicdaWaaakiabcIcaOiabgkHiTiabigdaXiabcMcaPmaaCaaaleqabaGaemyBa0gaaaqaaiabd2eannaaBaaameaacqaIXaqmaeqaaSGaeyypa0JaeyOeI0IaemitaW0aaSbaaWqaaiabigdaXaqabaaaleaacqWGmbatdaWgaaadbaGaeGymaedabeaaa0GaeyyeIuoakiaaxMaacaWLjaWaaeWaaeaacqaIZaWmcqaI0aanaiaawIcacaGLPaaaaaa@7811@

so that:

νaR(τ)=h10(τ)27∑M1=−L1L1ℚ1−M10ℚ1M10(−1)m(4π)3∫aR∫aRd|x→1|d|x→′1|{|x→′1|1|x→1|2;|x→′1|<|x→1||x→1|1|x→′1|2;|x→′1|≥|x→1|}=h10(τ)27∑M1=−L1L1ℚ1−M10ℚ1M10(−1)m(4π)3(12a−12R)(R−a)2     (35)
MathType@MTEF@5@5@+=feaafiart1ev1aaatCvAUfKttLearuWrP9MDH5MBPbIqV92AaeXatLxBI9gBaebbnrfifHhDYfgasaacH8akY=wiFfYdH8Gipec8Eeeu0xXdbba9frFj0=OqFfea0dXdd9vqai=hGuQ8kuc9pgc9s8qqaq=dirpe0xb9q8qiLsFr0=vr0=vr0dc8meaabaqaciaacaGaaeqabaqabeGadaaakeaafaqaaeGabaaabaacciGae8xVd42aaSbaaSqaaiabdggaHjabdkfasbqabaGccqGGOaakcqWFepaDcqGGPaqkcqGH9aqpdaWcaaqaaiabdIgaOnaaDaaaleaacqaIXaqmaeaacqaIWaamaaGccqGGOaakcqaHepaDcqGGPaqkaeaacqaIYaGmcqaI3aWnaaWaaabCaeaatuuDJXwAK1uy0HMmaeHbfv3ySLgzG0uy0HgiuD3BaGabaiab+PrirnaaDaaaleaacqaIXaqmcqGHsislcqWGnbqtdaWgaaadbaGaeGymaedabeaaaSqaaiabicdaWaaaaeaacqWGnbqtdaWgaaadbaGaeGymaedabeaaliabg2da9iabgkHiTiabdYeamnaaBaaameaacqaIXaqmaeqaaaWcbaGaemitaW0aaSbaaWqaaiabigdaXaqabaaaniabggHiLdGccqGFAesudaqhaaWcbaGaeGymaeJaemyta00aaSbaaWqaaiabigdaXaqabaaaleaacqaIWaamaaGccqGGOaakcqGHsislcqaIXaqmcqGGPaqkdaahaaWcbeqaaiabd2gaTbaakiabcIcaOiabisda0iab=b8aWjabcMcaPmaaCaaaleqabaGaeG4mamdaaOWaa8qCaeaadaWdXbqaaiabdsgaKnaaemaabaGafmiEaGNbaSaadaWgaaWcbaGaeGymaedabeaaaOGaay5bSlaawIa7aiabdsgaKnaaemaabaGafmiEaGNbaSGbauaadaWgaaWcbaGaeGymaedabeaaaOGaay5bSlaawIa7aaWcbaGaemyyaegabaGaemOuaifaniabgUIiYdaaleaacqWGHbqyaeaacqWGsbGua0Gaey4kIipakmaacmqabaqbaeqabiqaaaqaamaalaaabaWaaqWaaeaacuWG4baEgaWcgaqbamaaBaaaleaacqaIXaqmaeqaaaGccaGLhWUaayjcSdWaaWbaaSqabeaacqaIXaqmaaaakeaadaabdaqaaiqbdIha4zaalaWaaSbaaSqaaiabigdaXaqabaaakiaawEa7caGLiWoadaahaaWcbeqaaiabikdaYaaaaaGccqGG7aWodaabdaqaaiqbdIha4zaalyaafaWaaSbaaSqaaiabigdaXaqabaaakiaawEa7caGLiWoacqGH8aapdaabdaqaaiqbdIha4zaalaWaaSbaaSqaaiabigdaXaqabaaakiaawEa7caGLiWoaaeaadaWcaaqaamaaemaabaGafmiEaGNbaSaadaWgaaWcbaGaeGymaedabeaaaOGaay5bSlaawIa7amaaCaaaleqabaGaeGymaedaaaGcbaWaaqWaaeaacuWG4baEgaWcgaqbamaaBaaaleaacqaIXaqmaeqaaaGccaGLhWUaayjcSdWaaWbaaSqabeaacqaIYaGmaaaaaOGaei4oaSZaaqWaaeaacuWG4baEgaWcgaqbamaaBaaaleaacqaIXaqmaeqaaaGccaGLhWUaayjcSdGaeyyzIm7aaqWaaeaacuWG4baEgaWcamaaBaaaleaacqaIXaqmaeqaaaGccaGLhWUaayjcSdaaaaGaay5Eaiaaw2haaaqaaiabg2da9maalaaabaGaemiAaG2aa0baaSqaaiabigdaXaqaaiabicdaWaaakiabcIcaOiab=r8a0jabcMcaPaqaaiabikdaYiabiEda3aaadaaeWbqaaiab+PrirnaaDaaaleaacqaIXaqmcqGHsislcqWGnbqtdaWgaaadbaGaeGymaedabeaaaSqaaiabicdaWaaakiab+PrirnaaDaaaleaacqaIXaqmcqWGnbqtdaWgaaadbaGaeGymaedabeaaaSqaaiabicdaWaaakiabcIcaOiabgkHiTiabigdaXiabcMcaPmaaCaaaleqabaGaemyBa0gaaOGaeiikaGIaeGinaqJae8hWdaNaeiykaKYaaWbaaSqabeaacqaIZaWmaaaabaGaemyta00aaSbaaWqaaiabigdaXaqabaWccqGH9aqpcqGHsislcqWGmbatdaWgaaadbaGaeGymaedabeaaaSqaaiabdYeamnaaBaaameaacqaIXaqmaeqaaaqdcqGHris5aOWaaeWaaeaadaWcaaqaaiabigdaXaqaaiabikdaYiabdggaHbaacqGHsisldaWcaaqaaiabigdaXaqaaiabikdaYiabdkfasbaaaiaawIcacaGLPaaacqGGOaakcqWGsbGucqGHsislcqWGHbqycqGGPaqkdaahaaWcbeqaaiabikdaYaaaaaGccaWLjaGaaCzcamaabmaabaGaeG4mamJaeGynaudacaGLOaGaayzkaaaaaa@FB73@

which diverges for large R. This solution is exact proving that for uniform dipole densities and p = 1 the autocovariance function does strongly depend on R, the size of the neural system.

## Appendix B–Responses recorded from multipolar electrodes

It is easy to extend the results to the case of bipolar or more complex recording electrodes. Consider a multipolar electrode in which the recorded potential *ϕ*_*s *_(x→
 MathType@MTEF@5@5@+=feaafiart1ev1aaatCvAUfKttLearuWrP9MDH5MBPbIqV92AaeXatLxBI9gBaebbnrfifHhDYfgasaacH8akY=wiFfYdH8Gipec8Eeeu0xXdbba9frFj0=OqFfea0dXdd9vqai=hGuQ8kuc9pgc9s8qqaq=dirpe0xb9q8qiLsFr0=vr0=vr0dc8meaabaqaciaacaGaaeqabaqabeGadaaakeaacuWG4baEgaWcaaaa@2E37@, *t*) is a linear combination of the potentials at a number of locations:

ϕs(x→,t)=∑i=1cεiϕ(x→+ξ→i,t)     (36)
 MathType@MTEF@5@5@+=feaafiart1ev1aaatCvAUfKttLearuWrP9MDH5MBPbIqV92AaeXatLxBI9gBaebbnrfifHhDYfgasaacH8akY=wiFfYdH8Gipec8Eeeu0xXdbba9frFj0=OqFfea0dXdd9vqai=hGuQ8kuc9pgc9s8qqaq=dirpe0xb9q8qiLsFr0=vr0=vr0dc8meaabaqaciaacaGaaeqabaqabeGadaaakeaaiiGacqWFvpGAdaWgaaWcbaGaem4CamhabeaakiabcIcaOiqbdIha4zaalaGaeiilaWIaemiDaqNaeiykaKIaeyypa0ZaaabCaeaacqWF1oqzdaWgaaWcbaGaemyAaKgabeaakiab=v9aQbWcbaGaemyAaKMaeyypa0JaeGymaedabaGaem4yamganiabggHiLdGccqGGOaakcuWG4baEgaWcaiabgUcaRiqb=57a4zaalaWaaSbaaSqaaiabdMgaPbqabaGccqGGSaalcqWG0baDcqGGPaqkcaWLjaGaaCzcamaabmaabaGaeG4mamJaeGOnaydacaGLOaGaayzkaaaaaa@512A@

where the ξ→i
 MathType@MTEF@5@5@+=feaafiart1ev1aaatCvAUfKttLearuWrP9MDH5MBPbIqV92AaeXatLxBI9gBaebbnrfifHhDYfgasaacH8akY=wiFfYdH8Gipec8Eeeu0xXdbba9frFj0=OqFfea0dXdd9vqai=hGuQ8kuc9pgc9s8qqaq=dirpe0xb9q8qiLsFr0=vr0=vr0dc8meaabaqaciaacaGaaeqabaqabeGadaaakeaaiiGacuWF+oaEgaWcamaaBaaaleaacqWGPbqAaeqaaaaa@300F@ are the vectors pointing from the geometric center of the electrode to each of the c locations where recordings are made (∑i=1cξ→i=0)
 MathType@MTEF@5@5@+=feaafiart1ev1aaatCvAUfKttLearuWrP9MDH5MBPbIqV92AaeXatLxBI9gBaebbnrfifHhDYfgasaacH8akY=wiFfYdH8Gipec8Eeeu0xXdbba9frFj0=OqFfea0dXdd9vqai=hGuQ8kuc9pgc9s8qqaq=dirpe0xb9q8qiLsFr0=vr0=vr0dc8meaabaqaciaacaGaaeqabaqabeGadaaakeaadaqadaqaamaaqahabaacciGaf8NVdGNbaSaadaWgaaWcbaGaemyAaKgabeaakiabg2da9iabicdaWaWcbaGaemyAaKMaeyypa0JaeGymaedabaGaem4yamganiabggHiLdaakiaawIcacaGLPaaaaaa@3A82@. The *ε*_*i *_are the weights determining how much the potential from each location contributes to the total recorded potential, recorded potential, Substituting the result (6) into (1) gives:

ϕs(x→,t)=∑i=1cεi∫Vd3x→′ρ(x→′,t)|x→+ξ→i−x→′|=∫Vd3x→′ρ′(x→′,t)|x→−x→′|ρ′(x→′,t)=∑i=1cεiρ(x→′−ξ→i,t)     (37)
 MathType@MTEF@5@5@+=feaafiart1ev1aaatCvAUfKttLearuWrP9MDH5MBPbIqV92AaeXatLxBI9gBaebbnrfifHhDYfgasaacH8akY=wiFfYdH8Gipec8Eeeu0xXdbba9frFj0=OqFfea0dXdd9vqai=hGuQ8kuc9pgc9s8qqaq=dirpe0xb9q8qiLsFr0=vr0=vr0dc8meaabaqaciaacaGaaeqabaqabeGadaaakeaafaqaaeGabaaabaacciGae8x1dO2aaSbaaSqaaiabdohaZbqabaGccqGGOaakcuWG4baEgaWcaiabcYcaSiabdsha0jabcMcaPiabg2da9maaqahabaGae8xTdu2aaSbaaSqaaiabdMgaPbqabaaabaGaemyAaKMaeyypa0JaeGymaedabaGaem4yamganiabggHiLdGcdaWdrbqaaiabdsgaKnaaCaaaleqabaGaeG4mamdaaOGafmiEaGNbaSGbauaaaSqaaiabdAfawbqab0Gaey4kIipakmaalaaabaGae8xWdiNaeiikaGIafmiEaGNbaSGbauaacqGGSaalcqWG0baDcqGGPaqkaeaacqGG8baFcuWG4baEgaWcaiabgUcaRiqb=57a4zaalaWaaSbaaSqaaiabdMgaPbqabaGccqGHsislcuWG4baEgaWcgaqbaiabcYha8baacqGH9aqpdaWdrbqaaiabdsgaKnaaCaaaleqabaGaeG4mamdaaOGafmiEaGNbaSGbauaaaSqaaiabdAfawbqab0Gaey4kIipakmaalaaabaGaf8xWdiNbauaacqGGOaakcuWG4baEgaWcgaqbaiabcYcaSiabdsha0jabcMcaPaqaaiabcYha8jqbdIha4zaalaGaeyOeI0IafmiEaGNbaSGbauaacqGG8baFaaaabaGaf8xWdiNbauaacqGGOaakcuWG4baEgaWcgaqbaiabcYcaSiabdsha0jabcMcaPiabg2da9maaqahabaGae8xTdu2aaSbaaSqaaiabdMgaPbqabaaabaGaemyAaKMaeyypa0JaeGymaedabaGaem4yamganiabggHiLdGccqWFbpGCcqGGOaakcuWG4baEgaWcgaqbaiabgkHiTiqb=57a4zaalaWaaSbaaSqaaiabdMgaPbqabaGccqGGSaalcqWG0baDcqGGPaqkaaGaaCzcaiaaxMaadaqadaqaaiabiodaZiabiEda3aGaayjkaiaawMcaaaaa@950C@

so that:

ϕs(x→,t)=∫4π∑l=0∞∑m=−ll12l+11|x→−x→1|l+1Ylm(θ1,φ1)Q′lm(x→1,t)d3x→1Q′lm(x→1,t)=1ΔVx→1∫ΔVx→1|x→″|lYlm∗(θ″,φ″)ρ′(x→1+x→″,t)d3x→″=1ΔVx→1∑i=1cεi∫ΔVx→1|x→″|lYlm∗(θ″,φ″)ρ(x→1+x→″−ξ→i,t)d3x→″     (38)
 MathType@MTEF@5@5@+=feaafiart1ev1aaatCvAUfKttLearuWrP9MDH5MBPbIqV92AaeXatLxBI9gBaebbnrfifHhDYfgasaacH8akY=wiFfYdH8Gipec8Eeeu0xXdbba9frFj0=OqFfea0dXdd9vqai=hGuQ8kuc9pgc9s8qqaq=dirpe0xb9q8qiLsFr0=vr0=vr0dc8meaabaqaciaacaGaaeqabaqabeGadaaakeaafaqaaeWabaaabaacciGae8x1dO2aaSbaaSqaaiabdohaZbqabaGccqGGOaakcuWG4baEgaWcaiabcYcaSiabdsha0jabcMcaPiabg2da9maapeaabaGaeGinaqJae8hWdahaleqabeqdcqGHRiI8aOWaaabCaeaadaaeWbqaamaalaaabaGaeGymaedabaGaeGOmaiJaemiBaWMaey4kaSIaeGymaedaamaalaaabaGaeGymaedabaWaaqWaaeaacuWG4baEgaWcaiabgkHiTiqbdIha4zaalaWaaSbaaSqaaiabigdaXaqabaaakiaawEa7caGLiWoadaahaaWcbeqaaiabdYgaSjabgUcaRiabigdaXaaaaaaabaGaemyBa0Maeyypa0JaeyOeI0IaemiBaWgabaGaemiBaWganiabggHiLdaaleaacqWGSbaBcqGH9aqpcqaIWaamaeaacqGHEisPa0GaeyyeIuoakiabdMfaznaaBaaaleaacqWGSbaBcqWGTbqBaeqaaOGaeiikaGIae8hUde3aaSbaaSqaaiabigdaXaqabaGccqGGSaalcqWFgpGzdaWgaaWcbaGaeGymaedabeaakiabcMcaPiqbdgfarzaafaWaaSbaaSqaaiabdYgaSjabd2gaTbqabaGccqGGOaakcuWG4baEgaWcamaaBaaaleaacqaIXaqmaeqaaOGaeiilaWIaemiDaqNaeiykaKIaemizaq2aaWbaaSqabeaacqaIZaWmaaGccuWG4baEgaWcamaaBaaaleaacqaIXaqmaeqaaaGcbaGafmyuaeLbauaadaWgaaWcbaGaemiBaWMaemyBa0gabeaakiabcIcaOiqbdIha4zaalaWaaSbaaSqaaiabigdaXaqabaGccqGGSaalcqWG0baDcqGGPaqkcqGH9aqpdaWcaaqaaiabigdaXaqaaiabfs5aejabdAfawnaaBaaaleaacuWG4baEgaWcamaaBaaameaacqaIXaqmaeqaaaWcbeaaaaGcdaWdrbqaamaaemaabaGafmiEaGNbaSGbayaaaiaawEa7caGLiWoadaahaaWcbeqaaiabdYgaSbaaaeaacqqHuoarcqWGwbGvdaWgaaadbaGafmiEaGNbaSaadaWgaaqaaiabigdaXaqabaaabeaaaSqab0Gaey4kIipakiabdMfaznaaDaaaleaacqWGSbaBcqWGTbqBaeaacqGHxiIkaaGccqGGOaakcuWF4oqCgaGbaiabcYcaSiqb=z8aMzaagaGaeiykaKIaf8xWdiNbauaacqGGOaakcuWG4baEgaWcamaaBaaaleaacqaIXaqmaeqaaOGaey4kaSIafmiEaGNbaSGbayaacqGGSaalcqWG0baDcqGGPaqkcqWGKbazdaahaaWcbeqaaiabiodaZaaakiqbdIha4zaalyaagaaabaGaeyypa0ZaaSaaaeaacqaIXaqmaeaacqqHuoarcqWGwbGvdaWgaaWcbaGafmiEaGNbaSaadaWgaaadbaGaeGymaedabeaaaSqabaaaaOWaaabCaeaacqWF1oqzdaWgaaWcbaGaemyAaKgabeaaaeaacqWGPbqAcqGH9aqpcqaIXaqmaeaacqWGJbWya0GaeyyeIuoakmaapefabaWaaqWaaeaacuWG4baEgaWcgaGbaaGaay5bSlaawIa7amaaCaaaleqabaGaemiBaWgaaaqaaiabfs5aejabdAfawnaaBaaameaacuWG4baEgaWcamaaBaaabaGaeGymaedabeaaaeqaaaWcbeqdcqGHRiI8aOGaemywaK1aa0baaSqaaiabdYgaSjabd2gaTbqaaiabgEHiQaaakiabcIcaOiqb=H7aXzaagaGaeiilaWIaf8NXdyMbayaacqGGPaqkcqWFbpGCcqGGOaakcuWG4baEgaWcamaaBaaaleaacqaIXaqmaeqaaOGaey4kaSIafmiEaGNbaSGbayaacqGHsislcuWF+oaEgaWcamaaBaaaleaacqWGPbqAaeqaaOGaeiilaWIaemiDaqNaeiykaKIaemizaq2aaWbaaSqabeaacqaIZaWmaaGccuWG4baEgaWcgaGbaaaacaWLjaGaaCzcamaabmaabaGaeG4mamJaeGioaGdacaGLOaGaayzkaaaaaa@F6B0@

This demonstrates that the arguments used above apply equally to recording from complex electrodes as well as to simple single electrodes by use of the modified multipole moments *Q*_*lm*_'(x→
 MathType@MTEF@5@5@+=feaafiart1ev1aaatCvAUfKttLearuWrP9MDH5MBPbIqV92AaeXatLxBI9gBaebbnrfifHhDYfgasaacH8akY=wiFfYdH8Gipec8Eeeu0xXdbba9frFj0=OqFfea0dXdd9vqai=hGuQ8kuc9pgc9s8qqaq=dirpe0xb9q8qiLsFr0=vr0=vr0dc8meaabaqaciaacaGaaeqabaqabeGadaaakeaacuWG4baEgaWcaaaa@2E37@_1_, *t*).

The second important question is how does the use of a complex electrode change the recorded potentials from electric multipoles. First, it is well known^3 ^that the lowest order non-vanishing multipole moment of any charge distribution is independent of the origin of coordinates. Now, in (39) it can be seen that the effect of recording from different electrodes ξ→i
 MathType@MTEF@5@5@+=feaafiart1ev1aaatCvAUfKttLearuWrP9MDH5MBPbIqV92AaeXatLxBI9gBaebbnrfifHhDYfgasaacH8akY=wiFfYdH8Gipec8Eeeu0xXdbba9frFj0=OqFfea0dXdd9vqai=hGuQ8kuc9pgc9s8qqaq=dirpe0xb9q8qiLsFr0=vr0=vr0dc8meaabaqaciaacaGaaeqabaqabeGadaaakeaaiiGacuWF+oaEgaWcamaaBaaaleaacqWGPbqAaeqaaaaa@300F@ is essentially equivalent to a change in the origin of coordinates in the computation of the multipole moment and so does not affect the lowest order moment. This means that as long as ∑i=1cεi=0
 MathType@MTEF@5@5@+=feaafiart1ev1aaatCvAUfKttLearuWrP9MDH5MBPbIqV92AaeXatLxBI9gBaebbnrfifHhDYfgasaacH8akY=wiFfYdH8Gipec8Eeeu0xXdbba9frFj0=OqFfea0dXdd9vqai=hGuQ8kuc9pgc9s8qqaq=dirpe0xb9q8qiLsFr0=vr0=vr0dc8meaabaqaciaacaGaaeqabaqabeGadaaakeaadaaeWbqaaGGaciab=v7aLnaaBaaaleaacqWGPbqAaeqaaOGaeyypa0JaeGimaadaleaacqWGPbqAcqGH9aqpcqaIXaqmaeaacqWGJbWya0GaeyyeIuoaaaa@38C1@ the lowest order multipole moment disappears. Thus, for example, a bipolar recording from dipolar sources are dominated by the l = 2 terms in the multipole expansion and bipolar recordings of the quadrupolar sources are dominanted by the l = 3 terms.

## Appendix C–Evaluation of the cross covariance function

In order to determine the cross covariance, it is necessary to evaluate:

νaR(x→,s→,τ)(4π)2=∫Vd3x→1∑l=0∞∑l′=0∞∑m=ll∑m′=−l′l′12l+112l′+11|x→−x→1|l+11|x→+s→−x→1|l′+1Ylm(θ1,φ1)Yl′m′(θ1,φ1)ℚlm(x→1)ℚl′m′(x→1)h(x→1,τ)     (39)
 MathType@MTEF@5@5@+=feaafiart1ev1aaatCvAUfKttLearuWrP9MDH5MBPbIqV92AaeXatLxBI9gBaebbnrfifHhDYfgasaacH8akY=wiFfYdH8Gipec8Eeeu0xXdbba9frFj0=OqFfea0dXdd9vqai=hGuQ8kuc9pgc9s8qqaq=dirpe0xb9q8qiLsFr0=vr0=vr0dc8meaabaqaciaacaGaaeqabaqabeGadaaakqaabeqaamaalaaabaacciGae8xVd42aaSbaaSqaaiabdggaHjabdkfasbqabaGccqGGOaakcuWG4baEgaWcaiabcYcaSiqbdohaZzaalaGaeiilaWIae8hXdqNaeiykaKcabaGaeiikaGIaeGinaqJae8hWdaNaeiykaKYaaWbaaSqabeaacqaIYaGmaaaaaOGaeyypa0dabaWaa8quaeaacqWGKbazdaahaaWcbeqaaiabiodaZaaakiqbdIha4zaalaWaaSbaaSqaaiabigdaXaqabaaabaGaemOvayfabeqdcqGHRiI8aOWaaabCaeaadaaeWbqaamaaqahabaWaaabCaeaadaWcaaqaaiabigdaXaqaaiabikdaYiabdYgaSjabgUcaRiabigdaXaaadaWcaaqaaiabigdaXaqaaiabikdaYiqbdYgaSzaafaGaey4kaSIaeGymaedaamaalaaabaGaeGymaedabaWaaqWaaeaacuWG4baEgaWcaiabgkHiTiqbdIha4zaalaWaaSbaaSqaaiabigdaXaqabaaakiaawEa7caGLiWoadaahaaWcbeqaaiabdYgaSjabgUcaRiabigdaXaaaaaaabaGafmyBa0MbauaacqGH9aqpcqGHsislcuWGSbaBgaqbaaqaaiqbdYgaSzaafaaaniabggHiLdaaleaacqWGTbqBcqGH9aqpcqWGSbaBaeaacqWGSbaBa0GaeyyeIuoaaSqaaiqbdYgaSzaafaGaeyypa0JaeGimaadabaGaeyOhIukaniabggHiLdaaleaacqWGSbaBcqGH9aqpcqaIWaamaeaacqGHEisPa0GaeyyeIuoakmaalaaabaGaeGymaedabaWaaqWaaeaacuWG4baEgaWcaiabgUcaRiqbdohaZzaalaGaeyOeI0IafmiEaGNbaSaadaWgaaWcbaGaeGymaedabeaaaOGaay5bSlaawIa7amaaCaaaleqabaGafmiBaWMbauaacqGHRaWkcqaIXaqmaaaaaOGaemywaK1aaSbaaSqaaiabdYgaSjabd2gaTbqabaGccqGGOaakcqWF4oqCdaWgaaWcbaGaeGymaedabeaakiabcYcaSiab=z8aMnaaBaaaleaacqaIXaqmaeqaaOGaeiykaKIaemywaK1aaSbaaSqaaiqbdYgaSzaafaGafmyBa0MbauaaaeqaaOGaeiikaGIae8hUde3aaSbaaSqaaiabigdaXaqabaGccqGGSaalcqWFgpGzdaWgaaWcbaGaeGymaedabeaakiabcMcaPmrr1ngBPrwtHrhAYaqeguuDJXwAKbstHrhAGq1DVbaceaGae4NgHe1aaSbaaSqaaiabdYgaSjabd2gaTbqabaGccqGGOaakcuWG4baEgaWcamaaBaaaleaacqaIXaqmaeqaaOGaeiykaKIae4NgHe1aaSbaaSqaaiqbdYgaSzaafaGafmyBa0MbauaaaeqaaOGaeiikaGIafmiEaGNbaSaadaWgaaWcbaGaeGymaedabeaakiabcMcaPiabdIgaOjabcIcaOiqbdIha4zaalaWaaSbaaSqaaiabigdaXaqabaGccqGGSaalcqWFepaDcqGGPaqkcaWLjaGaaCzcamaabmaabaGaeG4mamJaeGyoaKdacaGLOaGaayzkaaaaaaa@CD82@

Consider the case x→
 MathType@MTEF@5@5@+=feaafiart1ev1aaatCvAUfKttLearuWrP9MDH5MBPbIqV92AaeXatLxBI9gBaebbnrfifHhDYfgasaacH8akY=wiFfYdH8Gipec8Eeeu0xXdbba9frFj0=OqFfea0dXdd9vqai=hGuQ8kuc9pgc9s8qqaq=dirpe0xb9q8qiLsFr0=vr0=vr0dc8meaabaqaciaacaGaaeqabaqabeGadaaakeaacuWG4baEgaWcaaaa@2E37@ = 0:

νaR(0,s→,τ)(4π)2=∫Vd3x→1∑l=0∞∑l′=0∞∑m=−ll∑m′=−l′l′12l+112l′+11|x→1|l+11|s→−x→1|l′+1Ylm(θ1,φ1)Yl′m′(θ1,φ1)ℚlm(x→1)ℚl′m′(x→1)h(x→1,τ)     (40)
 MathType@MTEF@5@5@+=feaafiart1ev1aaatCvAUfKttLearuWrP9MDH5MBPbIqV92AaeXatLxBI9gBaebbnrfifHhDYfgasaacH8akY=wiFfYdH8Gipec8Eeeu0xXdbba9frFj0=OqFfea0dXdd9vqai=hGuQ8kuc9pgc9s8qqaq=dirpe0xb9q8qiLsFr0=vr0=vr0dc8meaabaqaciaacaGaaeqabaqabeGadaaakqaabeqaamaalaaabaacciGae8xVd42aaSbaaSqaaiabdggaHjabdkfasbqabaGccqGGOaakcqaIWaamcqGGSaalcuWGZbWCgaWcaiabcYcaSiab=r8a0jabcMcaPaqaaiabcIcaOiabisda0iab=b8aWjabcMcaPmaaCaaaleqabaGaeGOmaidaaaaakiabg2da9aqaamaapefabaGaemizaq2aaWbaaSqabeaacqaIZaWmaaGccuWG4baEgaWcamaaBaaaleaacqaIXaqmaeqaaaqaaiabdAfawbqab0Gaey4kIipakmaaqahabaWaaabCaeaadaaeWbqaamaaqahabaWaaSaaaeaacqaIXaqmaeaacqaIYaGmcqWGSbaBcqGHRaWkcqaIXaqmaaWaaSaaaeaacqaIXaqmaeaacqaIYaGmcuWGSbaBgaqbaiabgUcaRiabigdaXaaadaWcaaqaaiabigdaXaqaamaaemaabaGafmiEaGNbaSaadaWgaaWcbaGaeGymaedabeaaaOGaay5bSlaawIa7amaaCaaaleqabaGaemiBaWMaey4kaSIaeGymaedaaaaaaeaacuWGTbqBgaqbaiabg2da9iabgkHiTiqbdYgaSzaafaaabaGafmiBaWMbauaaa0GaeyyeIuoaaSqaaiabd2gaTjabg2da9iabgkHiTiabdYgaSbqaaiabdYgaSbqdcqGHris5aaWcbaGafmiBaWMbauaacqGH9aqpcqaIWaamaeaacqGHEisPa0GaeyyeIuoaaSqaaiabdYgaSjabg2da9iabicdaWaqaaiabg6HiLcqdcqGHris5aOWaaSaaaeaacqaIXaqmaeaadaabdaqaaiqbdohaZzaalaGaeyOeI0IafmiEaGNbaSaadaWgaaWcbaGaeGymaedabeaaaOGaay5bSlaawIa7amaaCaaaleqabaGafmiBaWMbauaacqGHRaWkcqaIXaqmaaaaaOGaemywaK1aaSbaaSqaaiabdYgaSjabd2gaTbqabaGccqGGOaakcqWF4oqCdaWgaaWcbaGaeGymaedabeaakiabcYcaSiab=z8aMnaaBaaaleaacqaIXaqmaeqaaOGaeiykaKIaemywaK1aaSbaaSqaaiqbdYgaSzaafaGafmyBa0MbauaaaeqaaOGaeiikaGIae8hUde3aaSbaaSqaaiabigdaXaqabaGccqGGSaalcqWFgpGzdaWgaaWcbaGaeGymaedabeaakiabcMcaPmrr1ngBPrwtHrhAYaqeguuDJXwAKbstHrhAGq1DVbaceaGae4NgHe1aaSbaaSqaaiabdYgaSjabd2gaTbqabaGccqGGOaakcuWG4baEgaWcamaaBaaaleaacqaIXaqmaeqaaOGaeiykaKIae4NgHe1aaSbaaSqaaiqbdYgaSzaafaGafmyBa0MbauaaaeqaaOGaeiikaGIafmiEaGNbaSaadaWgaaWcbaGaeGymaedabeaakiabcMcaPiabdIgaOjabcIcaOiqbdIha4zaalaWaaSbaaSqaaiabigdaXaqabaGccqGGSaalcqWFepaDcqGGPaqkcaWLjaGaaCzcamaabmaabaGaeGinaqJaeGimaadacaGLOaGaayzkaaaaaaa@C8DD@

Note that:

1|s→−x→1|l′+1=(4π)l′+1∑Ll′+1=0∞∑Ml′+1=−Ll′+1Ll′+1⋯∑L1=0∞∑M1=−L1L1{|s→|∑i=1l′+1Li|x→1|∑i=1l′+1Li+l′+1;|s→|<|x→1||x→1|∑i=1l′+1Li|s→|∑i=1l′+1Li+l′+1;|s→|≥|x→1|}∏i=1l′+1[12Li+1YLiMi∗(θ1s,φ1s)YLiMi(θ1,φ1)]     (41)
 MathType@MTEF@5@5@+=feaafiart1ev1aaatCvAUfKttLearuWrP9MDH5MBPbIqV92AaeXatLxBI9gBaebbnrfifHhDYfgasaacH8akY=wiFfYdH8Gipec8Eeeu0xXdbba9frFj0=OqFfea0dXdd9vqai=hGuQ8kuc9pgc9s8qqaq=dirpe0xb9q8qiLsFr0=vr0=vr0dc8meaabaqaciaacaGaaeqabaqabeGadaaakeaadaWcaaqaaiabigdaXaqaamaaemaabaGafm4CamNbaSaacqGHsislcuWG4baEgaWcamaaBaaaleaacqaIXaqmaeqaaaGccaGLhWUaayjcSdWaaWbaaSqabeaacuWGSbaBgaqbaiabgUcaRiabigdaXaaaaaGccqGH9aqpcqGGOaakcqaI0aaniiGacqWFapaCcqGGPaqkdaahaaWcbeqaaiqbdYgaSzaafaGaey4kaSIaeGymaedaaOWaaabCaeaadaaeWbqaaiabl+UimnaaqahabaWaaabCaeaadaGadeqaauaabeqaceaaaeaadaWcaaqaamaaemaabaGafm4CamNbaSaaaiaawEa7caGLiWoadaahaaWcbeqaamaaqahabaGaemitaW0aaSbaaWqaaiabdMgaPbqabaaabaGaemyAaKMaeyypa0JaeGymaedabaGafmiBaWMbauaacqGHRaWkcqaIXaqma4GaeyyeIuoaaaaakeaadaabdaqaaiqbdIha4zaalaWaaSbaaSqaaiabigdaXaqabaaakiaawEa7caGLiWoadaahaaWcbeqaamaaqahabaGaemitaW0aaSbaaWqaaiabdMgaPbqabaWccqGHRaWkcuWGSbaBgaqbaiabgUcaRiabigdaXaadbaGaemyAaKMaeyypa0JaeGymaedabaGafmiBaWMbauaacqGHRaWkcqaIXaqma4GaeyyeIuoaaaaaaOGaei4oaSZaaqWaaeaacuWGZbWCgaWcaaGaay5bSlaawIa7aiabgYda8maaemaabaGafmiEaGNbaSaadaWgaaWcbaGaeGymaedabeaaaOGaay5bSlaawIa7aaqaamaalaaabaWaaqWaaeaacuWG4baEgaWcamaaBaaaleaacqaIXaqmaeqaaaGccaGLhWUaayjcSdWaaWbaaSqabeaadaaeWbqaaiabdYeamnaaBaaameaacqWGPbqAaeqaaaqaaiabdMgaPjabg2da9iabigdaXaqaaiqbdYgaSzaafaGaey4kaSIaeGymaedaoiabggHiLdaaaaGcbaWaaqWaaeaacuWGZbWCgaWcaaGaay5bSlaawIa7amaaCaaaleqabaWaaabCaeaacqWGmbatdaWgaaadbaGaemyAaKgabeaaliabgUcaRiqbdYgaSzaafaGaey4kaSIaeGymaedameaacqWGPbqAcqGH9aqpcqaIXaqmaeaacuWGSbaBgaqbaiabgUcaRiabigdaXaGdcqGHris5aaaaaaGccqGG7aWodaabdaqaaiqbdohaZzaalaaacaGLhWUaayjcSdGaeyyzIm7aaqWaaeaacuWG4baEgaWcamaaBaaaleaacqaIXaqmaeqaaaGccaGLhWUaayjcSdaaaaGaay5Eaiaaw2haamaarahabaWaamWaaeaadaWcaaqaaiabigdaXaqaaiabikdaYiabdYeamnaaBaaaleaacqWGPbqAaeqaaOGaey4kaSIaeGymaedaaiabdMfaznaaDaaaleaacqWGmbatdaWgaaadbaGaemyAaKgabeaaliabd2eannaaBaaameaacqWGPbqAaeqaaaWcbaGaey4fIOcaaOGaeiikaGIae8hUde3aa0baaSqaaiabigdaXaqaaiabdohaZbaakiabcYcaSiab=z8aMnaaDaaaleaacqaIXaqmaeaacqWGZbWCaaGccqGGPaqkcqWGzbqwdaWgaaWcbaGaemitaW0aaSbaaWqaaiabdMgaPbqabaWccqWGnbqtdaWgaaadbaGaemyAaKgabeaaaSqabaGccqGGOaakcqWF4oqCdaWgaaWcbaGaeGymaedabeaakiabcYcaSiab=z8aMnaaBaaaleaacqaIXaqmaeqaaOGaeiykaKcacaGLBbGaayzxaaaaleaacqWGPbqAcqGH9aqpcqaIXaqmaeaacuWGSbaBgaqbaiabgUcaRiabigdaXaqdcqGHpis1aaWcbaGaemyta00aaSbaaWqaaiabigdaXaqabaWccqGH9aqpcqGHsislcqWGmbatdaWgaaadbaGaeGymaedabeaaaSqaaiabdYeamnaaBaaameaacqaIXaqmaeqaaaqdcqGHris5aaWcbaGaemitaW0aaSbaaWqaaiabigdaXaqabaWccqGH9aqpcqaIWaamaeaacqGHEisPa0GaeyyeIuoaaSqaaiabd2eannaaBaaameaacuWGSbaBgaqbaiabgUcaRiabigdaXaqabaWccqGH9aqpcqGHsislcqWGmbatdaWgaaadbaGafmiBaWMbauaacqGHRaWkcqaIXaqmaeqaaaWcbaGaemitaW0aaSbaaWqaaiqbdYgaSzaafaGaey4kaSIaeGymaedabeaaa0GaeyyeIuoaaSqaaiabdYeamnaaBaaameaacuWGSbaBgaqbaiabgUcaRiabigdaXaqabaWccqGH9aqpcqaIWaamaeaacqGHEisPa0GaeyyeIuoakiaaxMaacaWLjaWaaeWaaeaacqaI0aancqaIXaqmaiaawIcacaGLPaaaaaa@1362@

and so:

νaR(0,s→,τ)(4π)2=∫Vd|x→1|dΩ1∑l=0∞∑l′=0∞∑m=−ll∑m′=−l′l′(4π)l′+12l+112l′+11|x→1|l−1∑Ll′+1=0∞∑Ml′+1=−Ll′+1Ll′+1⋯∑L1=0∞∑M1=−L1L1{|s→|∑i=1l′+1Li|x→1|∑i=1l′+1Li+l′+1;|s→|<|x→1||x→1|∑i=1l′+1Li|s→|∑i=1l′+1Li+l′+1;|s→|≥|x→1|}•∏i=1l′+1[12Li+1YLiMi∗(θ1s,φ1s)YLiMi(θ1,φ1)]Ylm(θ1,φ1)Yl′m′(θ1,φ1)ℚlm(x→1)ℚl′m′(x→1)h(x→1,τ)∫ΩdΩ1∑l=0∞∑l′=0∞∑m=−ll∑m′=−l′l′12l+1(4π)l′+12l′+1∑Ll′+1=0∞∑Ml′+1=−Ll′+1Ll′+1⋯∑L1=0∞∑M1=−L1L1{|s→|∑i=1l′+1Li∑i=1l′+1Li+l′+l−1[1|s→|∑i=1l′+1Li+l+l′−1−1r∑i=1l′+1Li+l′+l−1]+[|s→|∑i=1l′+1Li−l+2−a∑i=1l′+1Li−l+2](∑i=1l′+1Li−l+2)|s→|∑i=1l′+1Li+l′+1}•∏i=1l′+1[12Li+1YLiMi∗(θ1s,φ1s)YLiMi(θ1,φ1)]Ylm(θ1,φ1)Yl′m′(θ1,φ1)ℚlm(x→1)ℚl′m′(x→1)h(x→1,τ)     (42)
 MathType@MTEF@5@5@+=feaafiart1ev1aaatCvAUfKttLearuWrP9MDH5MBPbIqV92AaeXatLxBI9gBaebbnrfifHhDYfgasaacH8akY=wiFfYdH8Gipec8Eeeu0xXdbba9frFj0=OqFfea0dXdd9vqai=hGuQ8kuc9pgc9s8qqaq=dirpe0xb9q8qiLsFr0=vr0=vr0dc8meaabaqaciaacaGaaeqabaqabeGadaaakqaabeqaamaalaaabaacciGae8xVd42aaSbaaSqaaiabdggaHjabdkfasbqabaGccqGGOaakcqaIWaamcqGGSaalcuWGZbWCgaWcaiabcYcaSiab=r8a0jabcMcaPaqaaiabcIcaOiabisda0iab=b8aWjabcMcaPmaaCaaaleqabaGaeGOmaidaaaaakiabg2da9aqaamaapefabaGaemizaq2aaqWaaeaacuWG4baEgaWcamaaBaaaleaacqaIXaqmaeqaaaGccaGLhWUaayjcSdGaemizaqMaeuyQdC1aaSbaaSqaaiabigdaXaqabaaabaGaemOvayfabeqdcqGHRiI8aOWaaabCaeaadaaeWbqaamaaqahabaWaaabCaeaadaWcaaqaaiabcIcaOiabisda0iab=b8aWjabcMcaPmaaCaaaleqabaGafmiBaWMbauaacqGHRaWkcqaIXaqmaaaakeaacqaIYaGmcqWGSbaBcqGHRaWkcqaIXaqmaaWaaSaaaeaacqaIXaqmaeaacqaIYaGmcuWGSbaBgaqbaiabgUcaRiabigdaXaaadaWcaaqaaiabigdaXaqaamaaemaabaGafmiEaGNbaSaadaWgaaWcbaGaeGymaedabeaaaOGaay5bSlaawIa7amaaCaaaleqabaGaemiBaWMaeyOeI0IaeGymaedaaaaaaeaacuWGTbqBgaqbaiabg2da9iabgkHiTiqbdYgaSzaafaaabaGafmiBaWMbauaaa0GaeyyeIuoaaSqaaiabd2gaTjabg2da9iabgkHiTiabdYgaSbqaaiabdYgaSbqdcqGHris5aaWcbaGafmiBaWMbauaacqGH9aqpcqaIWaamaeaacqGHEisPa0GaeyyeIuoaaSqaaiabdYgaSjabg2da9iabicdaWaqaaiabg6HiLcqdcqGHris5aOWaaabCaeaadaaeWbqaaiabl+UimnaaqahabaWaaabCaeaadaGadeqaauaabeqaceaaaeaadaWcaaqaamaaemaabaGafm4CamNbaSaaaiaawEa7caGLiWoadaahaaWcbeqaamaaqahabaGaemitaW0aaSbaaWqaaiabdMgaPbqabaaabaGaemyAaKMaeyypa0JaeGymaedabaGafmiBaWMbauaacqGHRaWkcqaIXaqma4GaeyyeIuoaaaaakeaadaabdaqaaiqbdIha4zaalaWaaSbaaSqaaiabigdaXaqabaaakiaawEa7caGLiWoadaahaaWcbeqaamaaqahabaGaemitaW0aaSbaaWqaaiabdMgaPbqabaWccqGHRaWkcuWGSbaBgaqbaiabgUcaRiabigdaXaadbaGaemyAaKMaeyypa0JaeGymaedabaGafmiBaWMbauaacqGHRaWkcqaIXaqma4GaeyyeIuoaaaaaaOGaei4oaSZaaqWaaeaacuWGZbWCgaWcaaGaay5bSlaawIa7aiabgYda8maaemaabaGafmiEaGNbaSaadaWgaaWcbaGaeGymaedabeaaaOGaay5bSlaawIa7aaqaamaalaaabaWaaqWaaeaacuWG4baEgaWcamaaBaaaleaacqaIXaqmaeqaaaGccaGLhWUaayjcSdWaaWbaaSqabeaadaaeWbqaaiabdYeamnaaBaaameaacqWGPbqAaeqaaaqaaiabdMgaPjabg2da9iabigdaXaqaaiqbdYgaSzaafaGaey4kaSIaeGymaedaoiabggHiLdaaaaGcbaWaaqWaaeaacuWGZbWCgaWcaaGaay5bSlaawIa7amaaCaaaleqabaWaaabCaeaacqWGmbatdaWgaaadbaGaemyAaKgabeaaliabgUcaRiqbdYgaSzaafaGaey4kaSIaeGymaedameaacqWGPbqAcqGH9aqpcqaIXaqmaeaacuWGSbaBgaqbaiabgUcaRiabigdaXaGdcqGHris5aaaaaaGccqGG7aWodaabdaqaaiqbdohaZzaalaaacaGLhWUaayjcSdGaeyyzIm7aaqWaaeaacuWG4baEgaWcamaaBaaaleaacqaIXaqmaeqaaaGccaGLhWUaayjcSdaaaaGaay5Eaiaaw2haaaWcbaGaemyta00aaSbaaWqaaiabigdaXaqabaWccqGH9aqpcqGHsislcqWGmbatdaWgaaadbaGaeGymaedabeaaaSqaaiabdYeamnaaBaaameaacqaIXaqmaeqaaaqdcqGHris5aaWcbaGaemitaW0aaSbaaWqaaiabigdaXaqabaWccqGH9aqpcqaIWaamaeaacqGHEisPa0GaeyyeIuoaaSqaaiabd2eannaaBaaameaacuWGSbaBgaqbaiabgUcaRiabigdaXaqabaWccqGH9aqpcqGHsislcqWGmbatdaWgaaadbaGafmiBaWMbauaacqGHRaWkcqaIXaqmaeqaaaWcbaGaemitaW0aaSbaaWqaaiqbdYgaSzaafaGaey4kaSIaeGymaedabeaaa0GaeyyeIuoaaSqaaiabdYeamnaaBaaameaacuWGSbaBgaqbaiabgUcaRiabigdaXaqabaWccqGH9aqpcqaIWaamaeaacqGHEisPa0GaeyyeIuoaaOqaaiabgkci3oaarahabaWaamWaaeaadaWcaaqaaiabigdaXaqaaiabikdaYiabdYeamnaaBaaaleaacqWGPbqAaeqaaOGaey4kaSIaeGymaedaaiabdMfaznaaDaaaleaacqWGmbatdaWgaaadbaGaemyAaKgabeaaliabd2eannaaBaaameaacqWGPbqAaeqaaaWcbaGaey4fIOcaaOGaeiikaGIae8hUde3aa0baaSqaaiabigdaXaqaaiabdohaZbaakiabcYcaSiab=z8aMnaaDaaaleaacqaIXaqmaeaacqWGZbWCaaGccqGGPaqkcqWGzbqwdaWgaaWcbaGaemitaW0aaSbaaWqaaiabdMgaPbqabaWccqWGnbqtdaWgaaadbaGaemyAaKgabeaaaSqabaGccqGGOaakcqWF4oqCdaWgaaWcbaGaeGymaedabeaakiabcYcaSiab=z8aMnaaBaaaleaacqaIXaqmaeqaaOGaeiykaKcacaGLBbGaayzxaaaaleaacqWGPbqAcqGH9aqpcqaIXaqmaeaacuWGSbaBgaqbaiabgUcaRiabigdaXaqdcqGHpis1aOGaemywaK1aaSbaaSqaaiabdYgaSjabd2gaTbqabaGccqGGOaakcqWF4oqCdaWgaaWcbaGaeGymaedabeaakiabcYcaSiab=z8aMnaaBaaaleaacqaIXaqmaeqaaOGaeiykaKIaemywaK1aaSbaaSqaaiqbdYgaSzaafaGafmyBa0MbauaaaeqaaOGaeiikaGIae8hUde3aaSbaaSqaaiabigdaXaqabaGccqGGSaalcqWFgpGzdaWgaaWcbaGaeGymaedabeaakiabcMcaPmrr1ngBPrwtHrhAYaqeguuDJXwAKbstHrhAGq1DVbaceaGae4NgHe1aaSbaaSqaaiabdYgaSjabd2gaTbqabaGccqGGOaakcuWG4baEgaWcamaaBaaaleaacqaIXaqmaeqaaOGaeiykaKIae4NgHe1aaSbaaSqaaiqbdYgaSzaafaGafmyBa0MbauaaaeqaaOGaeiikaGIafmiEaGNbaSaadaWgaaWcbaGaeGymaedabeaakiabcMcaPiabdIgaOjabcIcaOiqbdIha4zaalaWaaSbaaSqaaiabigdaXaqabaGccqGGSaalcqWFepaDcqGGPaqkaeaafaqabeGabaaabaWaa8quaeaacqWGKbazcqqHPoWvdaWgaaWcbaGaeGymaedabeaaaeaacqqHPoWvaeqaniabgUIiYdGcdaaeWbqaamaaqahabaWaaabCaeaadaaeWbqaamaalaaabaGaeGymaedabaGaeGOmaiJaemiBaWMaey4kaSIaeGymaedaamaalaaabaGaeiikaGIaeGinaqJae8hWdaNaeiykaKYaaWbaaSqabeaacuWGSbaBgaqbaiabgUcaRiabigdaXaaaaOqaaiabikdaYiqbdYgaSzaafaGaey4kaSIaeGymaedaaaWcbaGafmyBa0MbauaacqGH9aqpcqGHsislcuWGSbaBgaqbaaqaaiqbdYgaSzaafaaaniabggHiLdaaleaacqWGTbqBcqGH9aqpcqGHsislcqWGSbaBaeaacqWGSbaBa0GaeyyeIuoaaSqaaiqbdYgaSzaafaGaeyypa0JaeGimaadabaGaeyOhIukaniabggHiLdaaleaacqWGSbaBcqGH9aqpcqaIWaamaeaacqGHEisPa0GaeyyeIuoakmaaqahabaWaaabCaeaacqWIVlctdaaeWbqaamaaqahabaWaaiWabeaafaqabeGabaaabaWaaSaaaeaadaabdaqaaiqbdohaZzaalaaacaGLhWUaayjcSdWaaWbaaSqabeaadaaeWbqaaiabdYeamnaaBaaameaacqWGPbqAaeqaaaqaaiabdMgaPjabg2da9iabigdaXaqaaiqbdYgaSzaafaGaey4kaSIaeGymaedaoiabggHiLdaaaaGcbaWaaabCaeaacqWGmbatdaWgaaWcbaGaemyAaKgabeaakiabgUcaRiqbdYgaSzaafaGaey4kaSIaemiBaWMaeyOeI0IaeGymaedaleaacqWGPbqAcqGH9aqpcqaIXaqmaeaacuWGSbaBgaqbaiabgUcaRiabigdaXaqdcqGHris5aaaakmaadmaabaWaaSaaaeaacqaIXaqmaeaadaabdaqaaiqbdohaZzaalaaacaGLhWUaayjcSdWaaWbaaSqabeaadaaeWbqaaiabdYeamnaaBaaameaacqWGPbqAaeqaaSGaey4kaSIaemiBaWMaey4kaSIafmiBaWMbauaacqGHsislcqaIXaqmaWqaaiabdMgaPjabg2da9iabigdaXaqaaiqbdYgaSzaafaGaey4kaSIaeGymaedaoiabggHiLdaaaaaakiabgkHiTmaalaaabaGaeGymaedabaGaemOCai3aaWbaaSqabeaadaaeWbqaaiabdYeamnaaBaaameaacqWGPbqAaeqaaSGaey4kaSIafmiBaWMbauaacqGHRaWkcqWGSbaBcqGHsislcqaIXaqmaWqaaiabdMgaPjabg2da9iabigdaXaqaaiqbdYgaSzaafaGaey4kaSIaeGymaedaoiabggHiLdaaaaaaaOGaay5waiaaw2faaiabgUcaRaqaamaalaaabaWaamWaaeaadaabdaqaaiqbdohaZzaalaaacaGLhWUaayjcSdWaaWbaaSqabeaadaaeWbqaaiabdYeamnaaBaaameaacqWGPbqAaeqaaSGaeyOeI0IaemiBaWMaey4kaSIaeGOmaidameaacqWGPbqAcqGH9aqpcqaIXaqmaeaacuWGSbaBgaqbaiabgUcaRiabigdaXaGdcqGHris5aaaakiabgkHiTiabdggaHnaaCaaaleqabaWaaabCaeaacqWGmbatdaWgaaadbaGaemyAaKgabeaaliabgkHiTiabdYgaSjabgUcaRiabikdaYaadbaGaemyAaKMaeyypa0JaeGymaedabaGafmiBaWMbauaacqGHRaWkcqaIXaqma4GaeyyeIuoaaaaakiaawUfacaGLDbaaaeaadaqadaqaauaabeqaceaaaeaadaaeWbqaaiabdYeamnaaBaaaleaacqWGPbqAaeqaaOGaeyOeI0IaemiBaWMaey4kaSIaeGOmaidaleaacqWGPbqAcqGH9aqpcqaIXaqmaeaacuWGSbaBgaqbaiabgUcaRiabigdaXaqdcqGHris5aaGcbaaaaaGaayjkaiaawMcaamaaemaabaGafm4CamNbaSaaaiaawEa7caGLiWoadaahaaWcbeqaamaaqahabaGaemitaW0aaSbaaWqaaiabdMgaPbqabaWccqGHRaWkcuWGSbaBgaqbaiabgUcaRiabigdaXaadbaGaemyAaKMaeyypa0JaeGymaedabaGafmiBaWMbauaacqGHRaWkcqaIXaqma4GaeyyeIuoaaaaaaaaaaOGaay5Eaiaaw2haaaWcbaGaemyta00aaSbaaWqaaiabigdaXaqabaWccqGH9aqpcqGHsislcqWGmbatdaWgaaadbaGaeGymaedabeaaaSqaaiabdYeamnaaBaaameaacqaIXaqmaeqaaaqdcqGHris5aaWcbaGaemitaW0aaSbaaWqaaiabigdaXaqabaWccqGH9aqpcqaIWaamaeaacqGHEisPa0GaeyyeIuoaaSqaaiabd2eannaaBaaameaacuWGSbaBgaqbaiabgUcaRiabigdaXaqabaWccqGH9aqpcqGHsislcqWGmbatdaWgaaadbaGafmiBaWMbauaacqGHRaWkcqaIXaqmaeqaaaWcbaGaemitaW0aaSbaaWqaaiqbdYgaSzaafaGaey4kaSIaeGymaedabeaaa0GaeyyeIuoaaSqaaiabdYeamnaaBaaameaacuWGSbaBgaqbaiabgUcaRiabigdaXaqabaWccqGH9aqpcqaIWaamaeaacqGHEisPa0GaeyyeIuoaaOqaaiabgkci3oaarahabaWaamWaaeaadaWcaaqaaiabigdaXaqaaiabikdaYiabdYeamnaaBaaaleaacqWGPbqAaeqaaOGaey4kaSIaeGymaedaaiabdMfaznaaDaaaleaacqWGmbatdaWgaaadbaGaemyAaKgabeaaliabd2eannaaBaaameaacqWGPbqAaeqaaaWcbaGaey4fIOcaaOGaeiikaGIae8hUde3aa0baaSqaaiabigdaXaqaaiabdohaZbaakiabcYcaSiab=z8aMnaaDaaaleaacqaIXaqmaeaacqWGZbWCaaGccqGGPaqkcqWGzbqwdaWgaaWcbaGaemitaW0aaSbaaWqaaiabdMgaPbqabaWccqWGnbqtdaWgaaadbaGaemyAaKgabeaaaSqabaGccqGGOaakcqWF4oqCdaWgaaWcbaGaeGymaedabeaakiabcYcaSiab=z8aMnaaBaaaleaacqaIXaqmaeqaaOGaeiykaKcacaGLBbGaayzxaaaaleaacqWGPbqAcqGH9aqpcqaIXaqmaeaacuWGSbaBgaqbaiabgUcaRiabigdaXaqdcqGHpis1aOGaemywaK1aaSbaaSqaaiabdYgaSjabd2gaTbqabaGccqGGOaakcqWF4oqCdaWgaaWcbaGaeGymaedabeaakiabcYcaSiab=z8aMnaaBaaaleaacqaIXaqmaeqaaOGaeiykaKIaemywaK1aaSbaaSqaaiqbdYgaSzaafaGafmyBa0MbauaaaeqaaOGaeiikaGIae8hUde3aaSbaaSqaaiabigdaXaqabaGccqGGSaalcqWFgpGzdaWgaaWcbaGaeGymaedabeaakiabcMcaPiab+PrirnaaBaaaleaacqWGSbaBcqWGTbqBaeqaaOGaeiikaGIafmiEaGNbaSaadaWgaaWcbaGaeGymaedabeaakiabcMcaPiab+PrirnaaBaaaleaacuWGSbaBgaqbaiqbd2gaTzaafaaabeaakiabcIcaOiqbdIha4zaalaWaaSbaaSqaaiabigdaXaqabaGccqGGPaqkcqWGObaAcqGGOaakcuWG4baEgaWcamaaBaaaleaacqaIXaqmaeqaaOGaeiilaWIae8hXdqNaeiykaKcaaiaaxMaacaWLjaWaaeWaaeaacqaI0aancqaIYaGmaiaawIcacaGLPaaaaaaa@0A8D@

when |s→
 MathType@MTEF@5@5@+=feaafiart1ev1aaatCvAUfKttLearuWrP9MDH5MBPbIqV92AaeXatLxBI9gBaebbnrfifHhDYfgasaacH8akY=wiFfYdH8Gipec8Eeeu0xXdbba9frFj0=OqFfea0dXdd9vqai=hGuQ8kuc9pgc9s8qqaq=dirpe0xb9q8qiLsFr0=vr0=vr0dc8meaabaqaciaacaGaaeqabaqabeGadaaakeaacuWGZbWCgaWcaaaa@2E2D@| > *a*. If we consider r->infinity and since l, l' > = 1 we get and a- > 0:

∫ΩdΩ1∑l=0∞∑l′=0∞∑m=−ll∑m′=−l′l′12l+112l′+1(4π)l′+1[1|s→|l+l′−1]∑Ll′+1=0∞∑Ml′+1=−Ll′+1Ll′+1⋯∑L1=0∞∑M1=−L1L1{1∑i=1l′+1Li+l′+l−1+1(∑i=1l′+1Li−l+2)}     (43)•∏i=1l′+1[12Li+1YLiMi∗(θ1s,φ1s)YLiMi(θ1,φ1)]Ylm(θ1,φ1)Yl′m′(θ1,φ1)ℚlm(x→1)ℚl′m′(x→1)h(x→1,τ)
 MathType@MTEF@5@5@+=feaafiart1ev1aaatCvAUfKttLearuWrP9MDH5MBPbIqV92AaeXatLxBI9gBaebbnrfifHhDYfgasaacH8akY=wiFfYdH8Gipec8Eeeu0xXdbba9frFj0=OqFfea0dXdd9vqai=hGuQ8kuc9pgc9s8qqaq=dirpe0xb9q8qiLsFr0=vr0=vr0dc8meaabaqaciaacaGaaeqabaqabeGadaaakqaabeqaamaapefabaGaemizaqMaeuyQdC1aaSbaaSqaaiabigdaXaqabaaabaGaeuyQdCfabeqdcqGHRiI8aOWaaabCaeaadaaeWbqaamaaqahabaWaaabCaeaadaWcaaqaaiabigdaXaqaaiabikdaYiabdYgaSjabgUcaRiabigdaXaaadaWcaaqaaiabigdaXaqaaiabikdaYiqbdYgaSzaafaGaey4kaSIaeGymaedaaiabcIcaOiabisda0GGaciab=b8aWjabcMcaPmaaCaaaleqabaGafmiBaWMbauaacqGHRaWkcqaIXaqmaaaabaGafmyBa0MbauaacqGH9aqpcqGHsislcuWGSbaBgaqbaaqaaiqbdYgaSzaafaaaniabggHiLdaaleaacqWGTbqBcqGH9aqpcqGHsislcqWGSbaBaeaacqWGSbaBa0GaeyyeIuoaaSqaaiqbdYgaSzaafaGaeyypa0JaeGimaadabaGaeyOhIukaniabggHiLdaaleaacqWGSbaBcqGH9aqpcqaIWaamaeaacqGHEisPa0GaeyyeIuoakmaadmaabaWaaSaaaeaacqaIXaqmaeaadaabdaqaaiqbdohaZzaalaaacaGLhWUaayjcSdWaaWbaaSqabeaacqWGSbaBcqGHRaWkcuWGSbaBgaqbaiabgkHiTiabigdaXaaaaaaakiaawUfacaGLDbaadaaeWbqaamaaqahabaGaeS47IW0aaabCaeaadaaeWbqaamaacmqabaqbaeqabiqaaaqaamaalaaabaGaeGymaedabaWaaabCaeaacqWGmbatdaWgaaWcbaGaemyAaKgabeaakiabgUcaRiqbdYgaSzaafaGaey4kaSIaemiBaWMaeyOeI0IaeGymaedaleaacqWGPbqAcqGH9aqpcqaIXaqmaeaacuWGSbaBgaqbaiabgUcaRiabigdaXaqdcqGHris5aaaakiabgUcaRaqaamaalaaabaGaeGymaedabaWaaeWaaeaafaqabeGabaaabaWaaabCaeaacqWGmbatdaWgaaWcbaGaemyAaKgabeaakiabgkHiTiabdYgaSjabgUcaRiabikdaYaWcbaGaemyAaKMaeyypa0JaeGymaedabaGafmiBaWMbauaacqGHRaWkcqaIXaqma0GaeyyeIuoaaOqaaaaaaiaawIcacaGLPaaaaaaaaaGaay5Eaiaaw2haaaWcbaGaemyta00aaSbaaWqaaiabigdaXaqabaWccqGH9aqpcqGHsislcqWGmbatdaWgaaadbaGaeGymaedabeaaaSqaaiabdYeamnaaBaaameaacqaIXaqmaeqaaaqdcqGHris5aaWcbaGaemitaW0aaSbaaWqaaiabigdaXaqabaWccqGH9aqpcqaIWaamaeaacqGHEisPa0GaeyyeIuoaaSqaaiabd2eannaaBaaameaacuWGSbaBgaqbaiabgUcaRiabigdaXaqabaWccqGH9aqpcqGHsislcqWGmbatdaWgaaadbaGafmiBaWMbauaacqGHRaWkcqaIXaqmaeqaaaWcbaGaemitaW0aaSbaaWqaaiqbdYgaSzaafaGaey4kaSIaeGymaedabeaaa0GaeyyeIuoaaSqaaiabdYeamnaaBaaameaacuWGSbaBgaqbaiabgUcaRiabigdaXaqabaWccqGH9aqpcqaIWaamaeaacqGHEisPa0GaeyyeIuoakiaaxMaacaWLjaWaaeWaaeaacqaI0aancqaIZaWmaiaawIcacaGLPaaaaeaacqGHIaYTdaqeWbqaamaadmaabaWaaSaaaeaacqaIXaqmaeaacqaIYaGmcqWGmbatdaWgaaWcbaGaemyAaKgabeaakiabgUcaRiabigdaXaaacqWGzbqwdaqhaaWcbaGaemitaW0aaSbaaWqaaiabdMgaPbqabaWccqWGnbqtdaWgaaadbaGaemyAaKgabeaaaSqaaiabgEHiQaaakiabcIcaOiab=H7aXnaaDaaaleaacqaIXaqmaeaacqWGZbWCaaGccqGGSaalcqWFgpGzdaqhaaWcbaGaeGymaedabaGaem4CamhaaOGaeiykaKIaemywaK1aaSbaaSqaaiabdYeamnaaBaaameaacqWGPbqAaeqaaSGaemyta00aaSbaaWqaaiabdMgaPbqabaaaleqaaOGaeiikaGIae8hUde3aaSbaaSqaaiabigdaXaqabaGccqGGSaalcqWFgpGzdaWgaaWcbaGaeGymaedabeaakiabcMcaPaGaay5waiaaw2faaaWcbaGaemyAaKMaeyypa0JaeGymaedabaGafmiBaWMbauaacqGHRaWkcqaIXaqma0Gaey4dIunakiabdMfaznaaBaaaleaacqWGSbaBcqWGTbqBaeqaaOGaeiikaGIae8hUde3aaSbaaSqaaiabigdaXaqabaGccqGGSaalcqWFgpGzdaWgaaWcbaGaeGymaedabeaakiabcMcaPiabdMfaznaaBaaaleaacuWGSbaBgaqbaiqbd2gaTzaafaaabeaakiabcIcaOiab=H7aXnaaBaaaleaacqaIXaqmaeqaaOGaeiilaWIae8NXdy2aaSbaaSqaaiabigdaXaqabaGccqGGPaqktuuDJXwAK1uy0HMmaeHbfv3ySLgzG0uy0HgiuD3BaGabaiab+PrirnaaBaaaleaacqWGSbaBcqWGTbqBaeqaaOGaeiikaGIafmiEaGNbaSaadaWgaaWcbaGaeGymaedabeaakiabcMcaPiab+PrirnaaBaaaleaacuWGSbaBgaqbaiqbd2gaTzaafaaabeaakiabcIcaOiqbdIha4zaalaWaaSbaaSqaaiabigdaXaqabaGccqGGPaqkcqWGObaAcqGGOaakcuWG4baEgaWcamaaBaaaleaacqaIXaqmaeqaaOGaeiilaWIae8hXdqNaeiykaKcaaaa@41F8@

Now let:

f(l,l′,θ1s,φ1s)=∫ΩdΩ112l+112l′+1∑m=−ll∑m′=−l′l′∑Ll′+1=0∞∑Ml′+1=−Ll′+1Ll′+1⋯∑L1=0∞∑M1=−L1L1{1∑i=1l′+1Li+l′+l−1+1(∑i=1l′+1Li−l+2)}•∏i=1l′+1[12Li+1YLiMi∗(θ1s,φ1s)YLiMi(θ1,φ1)]Ylm(θ1,φ1)Yl′m′(θ1,φ1)     (44)
 MathType@MTEF@5@5@+=feaafiart1ev1aaatCvAUfKttLearuWrP9MDH5MBPbIqV92AaeXatLxBI9gBaebbnrfifHhDYfgasaacH8akY=wiFfYdH8Gipec8Eeeu0xXdbba9frFj0=OqFfea0dXdd9vqai=hGuQ8kuc9pgc9s8qqaq=dirpe0xb9q8qiLsFr0=vr0=vr0dc8meaabaqaciaacaGaaeqabaqabeGadaaakeaafaqaceGabaaabaGaemOzayMaeiikaGIaemiBaWMaeiilaWIafmiBaWMbauaacqGGSaaliiGacqWF4oqCdaqhaaWcbaGaeGymaedabaGaem4CamhaaOGaeiilaWIae8NXdy2aa0baaSqaaiabigdaXaqaaiabdohaZbaakiabcMcaPiabg2da9maapefabaGaemizaqMaeuyQdC1aaSbaaSqaaiabigdaXaqabaaabaGaeuyQdCfabeqdcqGHRiI8aOWaaSaaaeaacqaIXaqmaeaacqaIYaGmcqWGSbaBcqGHRaWkcqaIXaqmaaWaaSaaaeaacqaIXaqmaeaacqaIYaGmcuWGSbaBgaqbaiabgUcaRiabigdaXaaadaaeWbqaamaaqahabaWaaabCaeaadaaeWbqaaiabl+UimbWcbaGaemyta00aaSbaaWqaaiqbdYgaSzaafaGaey4kaSIaeGymaedabeaaliabg2da9iabgkHiTiabdYeamnaaBaaameaacuWGSbaBgaqbaiabgUcaRiabigdaXaqabaaaleaacqWGmbatdaWgaaadbaGafmiBaWMbauaacqGHRaWkcqaIXaqmaeqaaaqdcqGHris5aaWcbaGaemitaW0aaSbaaWqaaiqbdYgaSzaafaGaey4kaSIaeGymaedabeaaliabg2da9iabicdaWaqaaiabg6HiLcqdcqGHris5aaWcbaGafmyBa0MbauaacqGH9aqpcqGHsislcuWGSbaBgaqbaaqaaiqbdYgaSzaafaaaniabggHiLdaaleaacqWGTbqBcqGH9aqpcqGHsislcqWGSbaBaeaacqWGSbaBa0GaeyyeIuoakmaaqahabaWaaabCaeaadaGadeqaauaabeqaceaaaeaadaWcaaqaaiabigdaXaqaamaaqahabaGaemitaW0aaSbaaSqaaiabdMgaPbqabaGccqGHRaWkcuWGSbaBgaqbaiabgUcaRiabdYgaSjabgkHiTiabigdaXaWcbaGaemyAaKMaeyypa0JaeGymaedabaGafmiBaWMbauaacqGHRaWkcqaIXaqma0GaeyyeIuoaaaGccqGHRaWkaeaadaWcaaqaaiabigdaXaqaamaabmaabaqbaeqabiqaaaqaamaaqahabaGaemitaW0aaSbaaSqaaiabdMgaPbqabaGccqGHsislcqWGSbaBcqGHRaWkcqaIYaGmaSqaaiabdMgaPjabg2da9iabigdaXaqaaiqbdYgaSzaafaGaey4kaSIaeGymaedaniabggHiLdaakeaaaaaacaGLOaGaayzkaaaaaaaaaiaawUhacaGL9baaaSqaaiabd2eannaaBaaameaacqaIXaqmaeqaaSGaeyypa0JaeyOeI0IaemitaW0aaSbaaWqaaiabigdaXaqabaaaleaacqWGmbatdaWgaaadbaGaeGymaedabeaaa0GaeyyeIuoaaSqaaiabdYeamnaaBaaameaacqaIXaqmaeqaaSGaeyypa0JaeGimaadabaGaeyOhIukaniabggHiLdaakeaacqGHIaYTdaqeWbqaamaadmaabaWaaSaaaeaacqaIXaqmaeaacqaIYaGmcqWGmbatdaWgaaWcbaGaemyAaKgabeaakiabgUcaRiabigdaXaaacqWGzbqwdaqhaaWcbaGaemitaW0aaSbaaWqaaiabdMgaPbqabaWccqWGnbqtdaWgaaadbaGaemyAaKgabeaaaSqaaiabgEHiQaaakiabcIcaOiab=H7aXnaaDaaaleaacqaIXaqmaeaacqWGZbWCaaGccqGGSaalcqWFgpGzdaqhaaWcbaGaeGymaedabaGaem4CamhaaOGaeiykaKIaemywaK1aaSbaaSqaaiabdYeamnaaBaaameaacqWGPbqAaeqaaSGaemyta00aaSbaaWqaaiabdMgaPbqabaaaleqaaOGaeiikaGIae8hUde3aaSbaaSqaaiabigdaXaqabaGccqGGSaalcqWFgpGzdaWgaaWcbaGaeGymaedabeaakiabcMcaPaGaay5waiaaw2faaaWcbaGaemyAaKMaeyypa0JaeGymaedabaGafmiBaWMbauaacqGHRaWkcqaIXaqma0Gaey4dIunakiabdMfaznaaBaaaleaacqWGSbaBcqWGTbqBaeqaaOGaeiikaGIae8hUde3aaSbaaSqaaiabigdaXaqabaGccqGGSaalcqWFgpGzdaWgaaWcbaGaeGymaedabeaakiabcMcaPiabdMfaznaaBaaaleaacuWGSbaBgaqbaiqbd2gaTzaafaaabeaakiabcIcaOiab=H7aXnaaBaaaleaacqaIXaqmaeqaaOGaeiilaWIae8NXdy2aaSbaaSqaaiabigdaXaqabaGccqGGPaqkaaGaaCzcaiaaxMaadaqadaqaaiabisda0iabisda0aGaayjkaiaawMcaaaaa@0DE7@

then we have:

νaR(0,s→,τ)(4π)2=∑l=0∞∑l′=0∞f(l,l′,θ1s,φ1s)[1|s→|l+l′−1]ℚlmℚl′m′h(τ)     (45)
 MathType@MTEF@5@5@+=feaafiart1ev1aaatCvAUfKttLearuWrP9MDH5MBPbIqV92AaeXatLxBI9gBaebbnrfifHhDYfgasaacH8akY=wiFfYdH8Gipec8Eeeu0xXdbba9frFj0=OqFfea0dXdd9vqai=hGuQ8kuc9pgc9s8qqaq=dirpe0xb9q8qiLsFr0=vr0=vr0dc8meaabaqaciaacaGaaeqabaqabeGadaaakeaadaWcaaqaaGGaciab=17aUnaaBaaaleaacqWGHbqycqWGsbGuaeqaaOGaeiikaGIaeGimaaJaeiilaWIafm4CamNbaSaacqGGSaalcqWFepaDcqGGPaqkaeaacqGGOaakcqaI0aancqWFapaCcqGGPaqkdaahaaWcbeqaaiabikdaYaaaaaGccqGH9aqpdaaeWbqaamaaqahabaGaemOzay2aaeWaaeaacqWGSbaBcqGGSaalcuWGSbaBgaqbaiabcYcaSiab=H7aXnaaDaaaleaacqaIXaqmaeaacqWGZbWCaaGccqGGSaalcqWFgpGzdaqhaaWcbaGaeGymaedabaGaem4CamhaaaGccaGLOaGaayzkaaWaamWaaeaadaWcaaqaaiabigdaXaqaaiabcYha8jqbdohaZzaalaGaeiiFaW3aaWbaaSqabeaacqWGSbaBcqGHRaWkcuWGSbaBgaqbaiabgkHiTiabigdaXaaaaaaakiaawUfacaGLDbaaaSqaaiqbdYgaSzaafaGaeyypa0JaeGimaadabaGaeyOhIukaniabggHiLdaaleaacqWGSbaBcqGH9aqpcqaIWaamaeaacqGHEisPa0GaeyyeIuoakiablQriKoaaBaaaleaacqWGSbaBcqWGTbqBaeqaaOGaeSOgHq6aaSbaaSqaaiqbdYgaSzaafaGafmyBa0MbauaaaeqaaOGaemiAaGMaeiikaGIae8hXdqNaeiykaKIaaCzcaiaaxMaadaqadaqaaiabisda0iabiwda1aGaayjkaiaawMcaaaaa@7E05@

So that for instance if the sources are dipolar (l, l' = 1) then νaR(0,s→,τ)(4π)2=1|s→|
 MathType@MTEF@5@5@+=feaafiart1ev1aaatCvAUfKttLearuWrP9MDH5MBPbIqV92AaeXatLxBI9gBaebbnrfifHhDYfgasaacH8akY=wiFfYdH8Gipec8Eeeu0xXdbba9frFj0=OqFfea0dXdd9vqai=hGuQ8kuc9pgc9s8qqaq=dirpe0xb9q8qiLsFr0=vr0=vr0dc8meaabaqaciaacaGaaeqabaqabeGadaaakeaadaWcaaqaaGGaciab=17aUnaaBaaaleaacqWGHbqycqWGsbGuaeqaaOGaeiikaGIaeGimaaJaeiilaWIafm4CamNbaSaacqGGSaalcqWFepaDcqGGPaqkaeaacqGGOaakcqaI0aancqWFapaCcqGGPaqkdaahaaWcbeqaaiabikdaYaaaaaGccqGH9aqpdaWcaaqaaiabigdaXaqaaiabcYha8jqbdohaZzaalaGaeiiFaWhaaaaa@44DA@. If the sources are quadrupolar, then l, l' = 2 and so νaR(0,s→,τ)(4π)2=1|s→|3
 MathType@MTEF@5@5@+=feaafiart1ev1aaatCvAUfKttLearuWrP9MDH5MBPbIqV92AaeXatLxBI9gBaebbnrfifHhDYfgasaacH8akY=wiFfYdH8Gipec8Eeeu0xXdbba9frFj0=OqFfea0dXdd9vqai=hGuQ8kuc9pgc9s8qqaq=dirpe0xb9q8qiLsFr0=vr0=vr0dc8meaabaqaciaacaGaaeqabaqabeGadaaakeaadaWcaaqaaGGaciab=17aUnaaBaaaleaacqWGHbqycqWGsbGuaeqaaOGaeiikaGIaeGimaaJaeiilaWIafm4CamNbaSaacqGGSaalcqWFepaDcqGGPaqkaeaacqGGOaakcqaI0aancqWFapaCcqGGPaqkdaahaaWcbeqaaiabikdaYaaaaaGccqGH9aqpdaWcaaqaaiabigdaXaqaaiabcYha8jqbdohaZzaalaGaeiiFaW3aaWbaaSqabeaacqaIZaWmaaaaaaaa@45FB@. This indicates that even when the neuronal cross covariance is a delta function, the measured cross covariance depends inversely on the distance between the electrodes.

## Appendix D-Qualitative estimate of the distance dependence of the cross covariance function

The purpose of this appendix is to present a simple qualitative argument illustrating the relationship between the cross covariance function of a simple scalar source distribution and the cross covariance function of the recorded electrical potentials. Consider the situation in which the relationship between the potential and the sources is given by:

ϕ(x→)=∫d3x→′ f(x→−x→′)ρ(x→′)     (46)
 MathType@MTEF@5@5@+=feaafiart1ev1aaatCvAUfKttLearuWrP9MDH5MBPbIqV92AaeXatLxBI9gBaebbnrfifHhDYfgasaacH8akY=wiFfYdH8Gipec8Eeeu0xXdbba9frFj0=OqFfea0dXdd9vqai=hGuQ8kuc9pgc9s8qqaq=dirpe0xb9q8qiLsFr0=vr0=vr0dc8meaabaqaciaacaGaaeqabaqabeGadaaakeaaiiGacqWFvpGAcqGGOaakcuWG4baEgaWcaiabcMcaPiabg2da9maapeaabaGaemizaq2aaWbaaSqabeaacqaIZaWmaaGccuWG4baEgaWcgaqbaaWcbeqab0Gaey4kIipakiabbccaGiabdAgaMjabcIcaOiqbdIha4zaalaGaeyOeI0IafmiEaGNbaSGbauaacqGGPaqkcqWFbpGCcqGGOaakcuWG4baEgaWcgaqbaiabcMcaPiaaxMaacaWLjaWaaeWaaeaacqaI0aancqaI2aGnaiaawIcacaGLPaaaaaa@4A75@

where *f*(x→
 MathType@MTEF@5@5@+=feaafiart1ev1aaatCvAUfKttLearuWrP9MDH5MBPbIqV92AaeXatLxBI9gBaebbnrfifHhDYfgasaacH8akY=wiFfYdH8Gipec8Eeeu0xXdbba9frFj0=OqFfea0dXdd9vqai=hGuQ8kuc9pgc9s8qqaq=dirpe0xb9q8qiLsFr0=vr0=vr0dc8meaabaqaciaacaGaaeqabaqabeGadaaakeaacuWG4baEgaWcaaaa@2E37@ - x→
 MathType@MTEF@5@5@+=feaafiart1ev1aaatCvAUfKttLearuWrP9MDH5MBPbIqV92AaeXatLxBI9gBaebbnrfifHhDYfgasaacH8akY=wiFfYdH8Gipec8Eeeu0xXdbba9frFj0=OqFfea0dXdd9vqai=hGuQ8kuc9pgc9s8qqaq=dirpe0xb9q8qiLsFr0=vr0=vr0dc8meaabaqaciaacaGaaeqabaqabeGadaaakeaacuWG4baEgaWcaaaa@2E37@') is the function that determines how the presence of an active generator at position x→
 MathType@MTEF@5@5@+=feaafiart1ev1aaatCvAUfKttLearuWrP9MDH5MBPbIqV92AaeXatLxBI9gBaebbnrfifHhDYfgasaacH8akY=wiFfYdH8Gipec8Eeeu0xXdbba9frFj0=OqFfea0dXdd9vqai=hGuQ8kuc9pgc9s8qqaq=dirpe0xb9q8qiLsFr0=vr0=vr0dc8meaabaqaciaacaGaaeqabaqabeGadaaakeaacuWG4baEgaWcaaaa@2E37@' influences the potential at x→
 MathType@MTEF@5@5@+=feaafiart1ev1aaatCvAUfKttLearuWrP9MDH5MBPbIqV92AaeXatLxBI9gBaebbnrfifHhDYfgasaacH8akY=wiFfYdH8Gipec8Eeeu0xXdbba9frFj0=OqFfea0dXdd9vqai=hGuQ8kuc9pgc9s8qqaq=dirpe0xb9q8qiLsFr0=vr0=vr0dc8meaabaqaciaacaGaaeqabaqabeGadaaakeaacuWG4baEgaWcaaaa@2E37@. In the ensuing calculations, it will be much simpler to deal with sources that are uniformly distributed than to be concerned about the details of how sources are distorted by the presence of an electrode of finite size. In what follows, the view will be taken that near the electrode *ρ*(x→
 MathType@MTEF@5@5@+=feaafiart1ev1aaatCvAUfKttLearuWrP9MDH5MBPbIqV92AaeXatLxBI9gBaebbnrfifHhDYfgasaacH8akY=wiFfYdH8Gipec8Eeeu0xXdbba9frFj0=OqFfea0dXdd9vqai=hGuQ8kuc9pgc9s8qqaq=dirpe0xb9q8qiLsFr0=vr0=vr0dc8meaabaqaciaacaGaaeqabaqabeGadaaakeaacuWG4baEgaWcaaaa@2E37@) is constant but:

f(x→−x→′=0;|x→−x→′|<a     (47)
 MathType@MTEF@5@5@+=feaafiart1ev1aaatCvAUfKttLearuWrP9MDH5MBPbIqV92AaeXatLxBI9gBaebbnrfifHhDYfgasaacH8akY=wiFfYdH8Gipec8Eeeu0xXdbba9frFj0=OqFfea0dXdd9vqai=hGuQ8kuc9pgc9s8qqaq=dirpe0xb9q8qiLsFr0=vr0=vr0dc8meaabaqaciaacaGaaeqabaqabeGadaaakeaacqWGMbGzcqGGOaakcuWG4baEgaWcaiabgkHiTiqbdIha4zaalyaafaGaeyypa0JaeGimaaJaei4oaSJaeiiFaWNafmiEaGNbaSaacqGHsislcuWG4baEgaWcgaqbaiabcYha8jabgYda8iabdggaHjaaxMaacaWLjaWaaeWaaeaacqaI0aancqaI3aWnaiaawIcacaGLPaaaaaa@43F5@

If the covariance function describing activity in different neural structures is given by:

〈ρ(x→) ρ(x→′)〉=α(x→−x→′)     (48)
 MathType@MTEF@5@5@+=feaafiart1ev1aaatCvAUfKttLearuWrP9MDH5MBPbIqV92AaeXatLxBI9gBaebbnrfifHhDYfgasaacH8akY=wiFfYdH8Gipec8Eeeu0xXdbba9frFj0=OqFfea0dXdd9vqai=hGuQ8kuc9pgc9s8qqaq=dirpe0xb9q8qiLsFr0=vr0=vr0dc8meaabaqaciaacaGaaeqabaqabeGadaaakeaadaaadeqaaGGaciab=f8aYjabcIcaOiqbdIha4zaalaGaeiykaKIaeeiiaaIae8xWdiNaeiikaGIafmiEaGNbaSGbauaacqGGPaqkaiaawMYicaGLQmcacqGH9aqpcqWFXoqycqGGOaakcuWG4baEgaWcaiabgkHiTiqbdIha4zaalyaafaGaeiykaKIaaCzcaiaaxMaadaqadaqaaiabisda0iabiIda4aGaayjkaiaawMcaaaaa@466C@

it is possible to use (47) and (48), to write the cross covariance of the recorded signal is:

〈ϕ(x→)ϕ(x→′)〉=∫d3x→1d3x→2f(x→−x→1)f(x→′−x→3)α(x→1−x→2)g(x→,s→)=〈ϕ(x→−s→2)ϕ(x→+s→2)〉=∫d3x→1d3x→2f(x→−s→2−x→1)f(x→+s→2−x→2)α(x→1−x→2)x→1′=x→1+s→2−x→x→2′=x→2−s→2−x→g(x→,s→)=∫d3x→1d3x→2f(−x→1′)f(−x→2′)α(x→1′−x→2′−s→)=∫d3x→1′d3x→2′f(x→1′)f(x→2′)α(x→1′−x→2′−s→)     (49)
 MathType@MTEF@5@5@+=feaafiart1ev1aaatCvAUfKttLearuWrP9MDH5MBPbIqV92AaeXatLxBI9gBaebbnrfifHhDYfgasaacH8akY=wiFfYdH8Gipec8Eeeu0xXdbba9frFj0=OqFfea0dXdd9vqai=hGuQ8kuc9pgc9s8qqaq=dirpe0xb9q8qiLsFr0=vr0=vr0dc8meaabaqaciaacaGaaeqabaqabeGadaaakeaafaqaaeqbbaaaaeaadaaadeqaaGGaciab=v9aQnaabmaabaGafmiEaGNbaSaaaiaawIcacaGLPaaacqWFvpGAdaqadaqaaiqbdIha4zaalyaafaaacaGLOaGaayzkaaaacaGLPmIaayPkJaGaeyypa0Zaa8qaaeaacqWGKbazdaahaaWcbeqaaiabiodaZaaakiqbdIha4zaalaWaaSbaaSqaaiabigdaXaqabaGccqWGKbazdaahaaWcbeqaaiabiodaZaaakiqbdIha4zaalaWaaSbaaSqaaiabikdaYaqabaGccqWGMbGzdaqadaqaaiqbdIha4zaalaGaeyOeI0IafmiEaGNbaSaadaWgaaWcbaGaeGymaedabeaaaOGaayjkaiaawMcaaaWcbeqab0Gaey4kIipakiabdAgaMnaabmaabaGafmiEaGNbaSGbauaacqGHsislcuWG4baEgaWcamaaBaaaleaacqaIZaWmaeqaaaGccaGLOaGaayzkaaGae8xSde2aaeWaaeaacuWG4baEgaWcamaaBaaaleaacqaIXaqmaeqaaOGaeyOeI0IafmiEaGNbaSaadaWgaaWcbaGaeGOmaidabeaaaOGaayjkaiaawMcaaaqaaiabdEgaNnaabmaabaGafmiEaGNbaSaacqGGSaalcuWGZbWCgaWcaaGaayjkaiaawMcaaiabg2da9maaamqabaGae8x1dO2aaeWaaeaacuWG4baEgaWcaiabgkHiTmaalaaabaGafm4CamNbaSaaaeaacqaIYaGmaaaacaGLOaGaayzkaaGae8x1dO2aaeWaaeaacuWG4baEgaWcaiabgUcaRmaalaaabaGafm4CamNbaSaaaeaacqaIYaGmaaaacaGLOaGaayzkaaaacaGLPmIaayPkJaGaeyypa0Zaa8qaaeaacqWGKbazdaahaaWcbeqaaiabiodaZaaakiqbdIha4zaalaWaaSbaaSqaaiabigdaXaqabaGccqWGKbazdaahaaWcbeqaaiabiodaZaaakiqbdIha4zaalaWaaSbaaSqaaiabikdaYaqabaGccqWGMbGzdaqadaqaaiqbdIha4zaalaGaeyOeI0YaaSaaaeaacuWGZbWCgaWcaaqaaiabikdaYaaacqGHsislcuWG4baEgaWcamaaBaaaleaacqaIXaqmaeqaaaGccaGLOaGaayzkaaGaemOzaygaleqabeqdcqGHRiI8aOWaaeWaaeaacuWG4baEgaWcaiabgUcaRmaalaaabaGafm4CamNbaSaaaeaacqaIYaGmaaGaeyOeI0IafmiEaGNbaSaadaWgaaWcbaGaeGOmaidabeaaaOGaayjkaiaawMcaaiab=f7aHnaabmaabaGafmiEaGNbaSaadaWgaaWcbaGaeGymaedabeaakiabgkHiTiqbdIha4zaalaWaaSbaaSqaaiabikdaYaqabaaakiaawIcacaGLPaaaaeaacuWG4baEgaWcamaaBaaaleaacqaIXaqmaeqaaGGaaOGae4NmGiQaeyypa0JafmiEaGNbaSaadaWgaaWcbaGaeGymaedabeaakiabgUcaRmaalaaabaGafm4CamNbaSaaaeaacqaIYaGmaaGaeyOeI0IafmiEaGNbaSaaaeaacuWG4baEgaWcamaaBaaaleaacqaIYaGmaeqaaOGae4NmGiQaeyypa0JafmiEaGNbaSaadaWgaaWcbaGaeGOmaidabeaakiabgkHiTmaalaaabaGafm4CamNbaSaaaeaacqaIYaGmaaGaeyOeI0IafmiEaGNbaSaaaeaacqWGNbWzdaqadaqaaiqbdIha4zaalaGaeiilaWIafm4CamNbaSaaaiaawIcacaGLPaaacqGH9aqpdaWdbaqaaiabdsgaKnaaCaaaleqabaGaeG4mamdaaOGafmiEaGNbaSaadaWgaaWcbaGaeGymaedabeaakiabdsgaKnaaCaaaleqabaGaeG4mamdaaOGafmiEaGNbaSaadaWgaaWcbaGaeGOmaidabeaakiabdAgaMnaabmaabaGaeyOeI0IafmiEaGNbaSaadaWgaaWcbaGaeGymaedabeaakiab+jdiIcGaayjkaiaawMcaaiabdAgaMnaabmaabaGaeyOeI0IafmiEaGNbaSaadaWgaaWcbaGaeGOmaidabeaakiab+jdiIcGaayjkaiaawMcaaiab=f7aHnaabmaabaGafmiEaGNbaSaadaWgaaWcbaGaeGymaedabeaakiab+jdiIkabgkHiTiqbdIha4zaalaWaaSbaaSqaaiabikdaYaqabaGccqGFYaIOcqGHsislcuWGZbWCgaWcaaGaayjkaiaawMcaaaWcbeqab0Gaey4kIipakiabg2da9maapeaabaGaemizaq2aaWbaaSqabeaacqaIZaWmaaGccuWG4baEgaWcamaaBaaaleaacqaIXaqmaeqaaOGae4NmGiQaemizaq2aaWbaaSqabeaacqaIZaWmaaGccuWG4baEgaWcamaaBaaaleaacqaIYaGmaeqaaOGae4NmGiQaemOzay2aaeWaaeaacuWG4baEgaWcamaaBaaaleaacqaIXaqmaeqaaOGae4NmGikacaGLOaGaayzkaaGaemOzay2aaeWaaeaacuWG4baEgaWcamaaBaaaleaacqaIYaGmaeqaaOGae4NmGikacaGLOaGaayzkaaGae8xSde2aaeWaaeaacuWG4baEgaWcamaaBaaaleaacqaIXaqmaeqaaOGae4NmGiQaeyOeI0IafmiEaGNbaSaadaWgaaWcbaGaeGOmaidabeaakiab+jdiIkabgkHiTiqbdohaZzaalaaacaGLOaGaayzkaaaaleqabeqdcqGHRiI8aaaakiaaxMaacaWLjaWaaeWaaeaacqaI0aancqaI5aqoaiaawIcacaGLPaaaaaa@27C0@

since *f *- (x→
 MathType@MTEF@5@5@+=feaafiart1ev1aaatCvAUfKttLearuWrP9MDH5MBPbIqV92AaeXatLxBI9gBaebbnrfifHhDYfgasaacH8akY=wiFfYdH8Gipec8Eeeu0xXdbba9frFj0=OqFfea0dXdd9vqai=hGuQ8kuc9pgc9s8qqaq=dirpe0xb9q8qiLsFr0=vr0=vr0dc8meaabaqaciaacaGaaeqabaqabeGadaaakeaacuWG4baEgaWcaaaa@2E37@) = ± *f*(x→
 MathType@MTEF@5@5@+=feaafiart1ev1aaatCvAUfKttLearuWrP9MDH5MBPbIqV92AaeXatLxBI9gBaebbnrfifHhDYfgasaacH8akY=wiFfYdH8Gipec8Eeeu0xXdbba9frFj0=OqFfea0dXdd9vqai=hGuQ8kuc9pgc9s8qqaq=dirpe0xb9q8qiLsFr0=vr0=vr0dc8meaabaqaciaacaGaaeqabaqabeGadaaakeaacuWG4baEgaWcaaaa@2E37@). Consider the scaling behavior as a function of s:

g(x→,εs→)=∫d3x→1′d3x→2′f(x→1′)f(x→2′)α(x→1′−x→2′−εs→)=∫d3x→1′d3x→2′f(x→1′)f(x→2′)α(ε[1εx→1′−1εx→2′−s→])x→1′′=1εx→1′x→2′′=1εx→2′g(x→,εs→)=ε2d∫d3x→1′′d3x→2′′f(εx→1′′)f(εx→2′′)α(ε[x→1′′−x→2′′−s→])     (50)
 MathType@MTEF@5@5@+=feaafiart1ev1aaatCvAUfKttLearuWrP9MDH5MBPbIqV92AaeXatLxBI9gBaebbnrfifHhDYfgasaacH8akY=wiFfYdH8Gipec8Eeeu0xXdbba9frFj0=OqFfea0dXdd9vqai=hGuQ8kuc9pgc9s8qqaq=dirpe0xb9q8qiLsFr0=vr0=vr0dc8meaabaqaciaacaGaaeqabaqabeGadaaakeaafaqaaeqbbaaaaeaacqWGNbWzdaqadaqaaiqbdIha4zaalaGaeiilaWccciGae8xTduMafm4CamNbaSaaaiaawIcacaGLPaaacqGH9aqpdaWdbaqaaiabdsgaKnaaCaaaleqabaGaeG4mamdaaaqabeqaniabgUIiYdGccuWG4baEgaWcamaaBaaaleaacqaIXaqmaeqaaGGaaOGae4NmGiQaemizaq2aaWbaaSqabeaacqaIZaWmaaGccuWG4baEgaWcamaaBaaaleaacqaIYaGmaeqaaOGae4NmGiQaemOzay2aaeWaaeaacuWG4baEgaWcamaaBaaaleaacqaIXaqmaeqaaOGae4NmGikacaGLOaGaayzkaaGaemOzay2aaeWaaeaacuWG4baEgaWcamaaBaaaleaacqaIYaGmaeqaaOGae4NmGikacaGLOaGaayzkaaGae8xSde2aaeWaaeaacuWG4baEgaWcamaaBaaaleaacqaIXaqmaeqaaOGae4NmGiQaeyOeI0IafmiEaGNbaSaadaWgaaWcbaGaeGOmaidabeaakiab+jdiIkabgkHiTiab=v7aLjqbdohaZzaalaaacaGLOaGaayzkaaaabaGaeyypa0Zaa8qaaeaacqWGKbazdaahaaWcbeqaaiabiodaZaaaaeqabeqdcqGHRiI8aOGafmiEaGNbaSaadaWgaaWcbaGaeGymaedabeaakiab+jdiIkabdsgaKnaaCaaaleqabaGaeG4mamdaaOGafmiEaGNbaSaadaWgaaWcbaGaeGOmaidabeaakiab+jdiIkabdAgaMnaabmaabaGafmiEaGNbaSaadaWgaaWcbaGaeGymaedabeaakiab+jdiIcGaayjkaiaawMcaaiabdAgaMnaabmaabaGafmiEaGNbaSaadaWgaaWcbaGaeGOmaidabeaakiab+jdiIcGaayjkaiaawMcaaiab=f7aHnaabmaabaGae8xTdu2aamWaaeaadaWcaaqaaiabigdaXaqaaiab=v7aLbaacuWG4baEgaWcamaaBaaaleaacqaIXaqmaeqaaOGae4NmGiQaeyOeI0YaaSaaaeaacqaIXaqmaeaacqWF1oqzaaGafmiEaGNbaSaadaWgaaWcbaGaeGOmaidabeaakiab+jdiIkabgkHiTiqbdohaZzaalaaacaGLBbGaayzxaaaacaGLOaGaayzkaaaabaGafmiEaGNbaSaadaWgaaWcbaGaeGymaedabeaakiab+jdiIkab+jdiIkabg2da9maalaaabaGaeGymaedabaGae8xTdugaaiqbdIha4zaalaWaaSbaaSqaaiabigdaXaqabaGccqGFYaIOaeaacuWG4baEgaWcamaaBaaaleaacqaIYaGmaeqaaOGae4NmGiQae4NmGiQaeyypa0ZaaSaaaeaacqaIXaqmaeaacqWF1oqzaaGafmiEaGNbaSaadaWgaaWcbaGaeGOmaidabeaakiab+jdiIcqaaiabdEgaNnaabmaabaGafmiEaGNbaSaacqGGSaalcqWF1oqzcuWGZbWCgaWcaaGaayjkaiaawMcaaiabg2da9iab=v7aLnaaCaaaleqabaGaeGOmaiJaemizaqgaaOWaa8qaaeaacqWGKbazdaahaaWcbeqaaiabiodaZaaaaeqabeqdcqGHRiI8aOGafmiEaGNbaSaadaWgaaWcbaGaeGymaedabeaakiab+jdiIkab+jdiIkabdsgaKnaaCaaaleqabaGaeG4mamdaaOGafmiEaGNbaSaadaWgaaWcbaGaeGOmaidabeaakiab+jdiIkab+jdiIkabdAgaMnaabmaabaGae8xTduMafmiEaGNbaSaadaWgaaWcbaGaeGymaedabeaakiab+jdiIkab+jdiIcGaayjkaiaawMcaaiabdAgaMnaabmaabaGae8xTduMafmiEaGNbaSaadaWgaaWcbaGaeGOmaidabeaakiab+jdiIkab+jdiIcGaayjkaiaawMcaaiab=f7aHnaabmaabaGae8xTdu2aamWaaeaacuWG4baEgaWcamaaBaaaleaacqaIXaqmaeqaaOGae4NmGiQae4NmGiQaeyOeI0IafmiEaGNbaSaadaWgaaWcbaGaeGOmaidabeaakiab+jdiIkab+jdiIkabgkHiTiqbdohaZzaalaaacaGLBbGaayzxaaaacaGLOaGaayzkaaaaaiaaxMaacaWLjaWaaeWaaeaacqaI1aqncqaIWaamaiaawIcacaGLPaaaaaa@02F5@

where d = 3. If:

α(εx→)=εpα(x→)     (51)
 MathType@MTEF@5@5@+=feaafiart1ev1aaatCvAUfKttLearuWrP9MDH5MBPbIqV92AaeXatLxBI9gBaebbnrfifHhDYfgasaacH8akY=wiFfYdH8Gipec8Eeeu0xXdbba9frFj0=OqFfea0dXdd9vqai=hGuQ8kuc9pgc9s8qqaq=dirpe0xb9q8qiLsFr0=vr0=vr0dc8meaabaqaciaacaGaaeqabaqabeGadaaakeaaiiGacqWFXoqycqGGOaakcqWF1oqzcuWG4baEgaWcaiabcMcaPiabg2da9iab=v7aLnaaCaaaleqabaGaemiCaahaaOGae8xSdeMaeiikaGIafmiEaGNbaSaacqGGPaqkcaWLjaGaaCzcamaabmaabaGaeGynauJaeGymaedacaGLOaGaayzkaaaaaa@4105@

(ie the correlations between neural structures declines as |x→
 MathType@MTEF@5@5@+=feaafiart1ev1aaatCvAUfKttLearuWrP9MDH5MBPbIqV92AaeXatLxBI9gBaebbnrfifHhDYfgasaacH8akY=wiFfYdH8Gipec8Eeeu0xXdbba9frFj0=OqFfea0dXdd9vqai=hGuQ8kuc9pgc9s8qqaq=dirpe0xb9q8qiLsFr0=vr0=vr0dc8meaabaqaciaacaGaaeqabaqabeGadaaakeaacuWG4baEgaWcaaaa@2E37@|^*P*^) for some number p, it is possible to write:

g(x→,εs→)=ε2d+p∫d3x→1′′d3x→2′′f(εx→1′′)f(εx→2′′)α([x→1′′−x→2′′−s→])     (52)
 MathType@MTEF@5@5@+=feaafiart1ev1aaatCvAUfKttLearuWrP9MDH5MBPbIqV92AaeXatLxBI9gBaebbnrfifHhDYfgasaacH8akY=wiFfYdH8Gipec8Eeeu0xXdbba9frFj0=OqFfea0dXdd9vqai=hGuQ8kuc9pgc9s8qqaq=dirpe0xb9q8qiLsFr0=vr0=vr0dc8meaabaqaciaacaGaaeqabaqabeGadaaakeaacqWGNbWzcqGGOaakcuWG4baEgaWcaiabcYcaSGGaciab=v7aLjqbdohaZzaalaGaeiykaKIaeyypa0Jae8xTdu2aaWbaaSqabeaacqaIYaGmcqWGKbazcqGHRaWkcqWGWbaCaaGcdaWdbaqaaiabdsgaKnaaCaaaleqabaGaeG4mamdaaaqabeqaniabgUIiYdGccuWG4baEgaWcamaaBaaaleaacqaIXaqmaeqaaGGaaOGae4NmGiQae4NmGiQaemizaq2aaWbaaSqabeaacqaIZaWmaaGccuWG4baEgaWcamaaBaaaleaacqaIYaGmaeqaaOGae4NmGiQae4NmGiQaemOzayMaeiikaGIae8xTduMafmiEaGNbaSaadaWgaaWcbaGaeGymaedabeaakiab+jdiIkab+jdiIkabcMcaPiabdAgaMjabcIcaOiab=v7aLjqbdIha4zaalaWaaSbaaSqaaiabikdaYaqabaGccqGFYaIOcqGFYaIOcqGGPaqkcqWFXoqycqGGOaakcqGGBbWwcuWG4baEgaWcamaaBaaaleaacqaIXaqmaeqaaOGae4NmGiQae4NmGiQaeyOeI0IafmiEaGNbaSaadaWgaaWcbaGaeGOmaidabeaakiab+jdiIkab+jdiIkabgkHiTiqbdohaZzaalaGaeiyxa0LaeiykaKIaaCzcaiaaxMaadaqadaqaaiabiwda1iabikdaYaGaayjkaiaawMcaaaaa@7CAA@

Because of the restriction (47), there can be no exact relation of the form:

f(εx→1′′)=εqf(x→1′′)
 MathType@MTEF@5@5@+=feaafiart1ev1aaatCvAUfKttLearuWrP9MDH5MBPbIqV92AaeXatLxBI9gBaebbnrfifHhDYfgasaacH8akY=wiFfYdH8Gipec8Eeeu0xXdbba9frFj0=OqFfea0dXdd9vqai=hGuQ8kuc9pgc9s8qqaq=dirpe0xb9q8qiLsFr0=vr0=vr0dc8meaabaqaciaacaGaaeqabaqabeGadaaakeaacqWGMbGzcqGGOaakiiGacqWF1oqzcuWG4baEgaWcamaaBaaaleaacqaIXaqmaeqaaGGaaOGae4NmGiQae4NmGiQaeiykaKIaeyypa0Jae8xTdu2aaWbaaSqabeaacqWGXbqCaaGccqWGMbGzcqGGOaakcuWG4baEgaWcamaaBaaaleaacqaIXaqmaeqaaOGae4NmGiQae4NmGiQaeiykaKcaaa@43FE@

However, if:

f(x→)=h(x→)θ(|x→|>a)h(εx→)=εqh(x→)     (53)
 MathType@MTEF@5@5@+=feaafiart1ev1aaatCvAUfKttLearuWrP9MDH5MBPbIqV92AaeXatLxBI9gBaebbnrfifHhDYfgasaacH8akY=wiFfYdH8Gipec8Eeeu0xXdbba9frFj0=OqFfea0dXdd9vqai=hGuQ8kuc9pgc9s8qqaq=dirpe0xb9q8qiLsFr0=vr0=vr0dc8meaabaqaciaacaGaaeqabaqabeGadaaakeaafaqaaeGabaaabaGaemOzayMaeiikaGIafmiEaGNbaSaacqGGPaqkcqGH9aqpcqWGObaAcqGGOaakcuWG4baEgaWcaiabcMcaPGGaciab=H7aXjabcIcaOiabcYha8jqbdIha4zaalaGaeiiFaWNaeyOpa4JaemyyaeMaeiykaKcabaGaemiAaGMaeiikaGIae8xTduMafmiEaGNbaSaacqGGPaqkcqGH9aqpcqWF1oqzdaahaaWcbeqaaiabdghaXbaakiabdIgaOjabcIcaOiqbdIha4zaalaGaeiykaKcaaiaaxMaacaWLjaWaaeWaaeaacqaI1aqncqaIZaWmaiaawIcacaGLPaaaaaa@5504@

for some function h and a number q:

g(x→,εs→)=ε2d+p+2q∫d3x→1′′d3x→2′′h(x→1′′)θ(|x→1′′|>aε)h(x→2′′)θ(|x→2′′|>aε)α([x→1′′−x→2′′−s→])=ε2d+p+2q∫d3x→1′′d3x→2′′h(x→1′′)h(x→2′′)θ(|x→1′′|>a)θ(|x→2′′|>a)α([x→1′′−x→2′′−s→])+ε2d+p+2q∫d3x→1′′d3x→2′′h(x→1′′)h(x→2′′)[θ(|x→1′′|>aε)θ(|x→2′′|>aε)−θ(|x→1′′|>a)θ(|x→2′′|>a)]α([x→1′′−x→2′′−s→])     (54)
 MathType@MTEF@5@5@+=feaafiart1ev1aaatCvAUfKttLearuWrP9MDH5MBPbIqV92AaeXatLxBI9gBaebbnrfifHhDYfgasaacH8akY=wiFfYdH8Gipec8Eeeu0xXdbba9frFj0=OqFfea0dXdd9vqai=hGuQ8kuc9pgc9s8qqaq=dirpe0xb9q8qiLsFr0=vr0=vr0dc8meaabaqaciaacaGaaeqabaqabeGadaaakeaafaqaaeWabaaabaGaem4zaC2aaeWaaeaacuWG4baEgaWcaiabcYcaSGGaciab=v7aLjqbdohaZzaalaaacaGLOaGaayzkaaGaeyypa0Jae8xTdu2aaWbaaSqabeaacqaIYaGmcqWGKbazcqGHRaWkcqWGWbaCcqGHRaWkcqaIYaGmcqWGXbqCaaGcdaWdbaqaaiabdsgaKnaaCaaaleqabaGaeG4mamdaaOGafmiEaGNbaSaadaWgaaWcbaGaeGymaedabeaaiiaakiab+jdiIkab+jdiIcWcbeqab0Gaey4kIipakiabdsgaKnaaCaaaleqabaGaeG4mamdaaOGafmiEaGNbaSaadaWgaaWcbaGaeGOmaidabeaakiab+jdiIkab+jdiIkabdIgaOnaabmaabaGafmiEaGNbaSaadaWgaaWcbaGaeGymaedabeaakiab+jdiIkab+jdiIcGaayjkaiaawMcaaiab=H7aXnaabmaabaWaaqWaaeaacuWG4baEgaWcamaaBaaaleaacqaIXaqmaeqaaOGae4NmGiQae4NmGikacaGLhWUaayjcSdGaeyOpa4ZaaSaaaeaacqWGHbqyaeaacqWF1oqzaaaacaGLOaGaayzkaaGaemiAaG2aaeWaaeaacuWG4baEgaWcamaaBaaaleaacqaIYaGmaeqaaOGae4NmGiQae4NmGikacaGLOaGaayzkaaGae8hUde3aaeWaaeaadaabdaqaaiqbdIha4zaalaWaaSbaaSqaaiabikdaYaqabaGccqGFYaIOcqGFYaIOaiaawEa7caGLiWoacqGH+aGpdaWcaaqaaiabdggaHbqaaiab=v7aLbaaaiaawIcacaGLPaaacqWFXoqydaqadaqaamaadmaabaGafmiEaGNbaSaadaWgaaWcbaGaeGymaedabeaakiab+jdiIkab+jdiIkabgkHiTiqbdIha4zaalaWaaSbaaSqaaiabikdaYaqabaGccqGFYaIOcqGFYaIOcqGHsislcuWGZbWCgaWcaaGaay5waiaaw2faaaGaayjkaiaawMcaaaqaaiabg2da9iab=v7aLnaaCaaaleqabaGaeGOmaiJaemizaqMaey4kaSIaemiCaaNaey4kaSIaeGOmaiJaemyCaehaaOWaa8qaaeaacqWGKbazdaahaaWcbeqaaiabiodaZaaakiqbdIha4zaalaWaaSbaaSqaaiabigdaXaqabaGccqGFYaIOcqGFYaIOaSqabeqaniabgUIiYdGccqWGKbazdaahaaWcbeqaaiabiodaZaaakiqbdIha4zaalaWaaSbaaSqaaiabikdaYaqabaGccqGFYaIOcqGFYaIOcqWGObaAdaqadaqaaiqbdIha4zaalaWaaSbaaSqaaiabigdaXaqabaGccqGFYaIOcqGFYaIOaiaawIcacaGLPaaacqWGObaAdaqadaqaaiqbdIha4zaalaWaaSbaaSqaaiabikdaYaqabaGccqGFYaIOcqGFYaIOaiaawIcacaGLPaaacqWF4oqCdaqadaqaamaaemaabaGafmiEaGNbaSaadaWgaaWcbaGaeGymaedabeaakiab+jdiIkab+jdiIcGaay5bSlaawIa7aiabg6da+iabdggaHbGaayjkaiaawMcaaiab=H7aXnaabmaabaWaaqWaaeaacuWG4baEgaWcamaaBaaaleaacqaIYaGmaeqaaOGae4NmGiQae4NmGikacaGLhWUaayjcSdGaeyOpa4JaemyyaegacaGLOaGaayzkaaGae8xSde2aaeWaaeaadaWadaqaaiqbdIha4zaalaWaaSbaaSqaaiabigdaXaqabaGccqGFYaIOcqGFYaIOcqGHsislcuWG4baEgaWcamaaBaaaleaacqaIYaGmaeqaaOGae4NmGiQae4NmGiQaeyOeI0Iafm4CamNbaSaaaiaawUfacaGLDbaaaiaawIcacaGLPaaaaeaacqGHRaWkcqWF1oqzdaahaaWcbeqaaiabikdaYiabdsgaKjabgUcaRiabdchaWjabgUcaRiabikdaYiabdghaXbaakmaapeaabaGaemizaq2aaWbaaSqabeaacqaIZaWmaaGccuWG4baEgaWcamaaBaaaleaacqaIXaqmaeqaaOGae4NmGiQae4NmGikaleqabeqdcqGHRiI8aOGaemizaq2aaWbaaSqabeaacqaIZaWmaaGccuWG4baEgaWcamaaBaaaleaacqaIYaGmaeqaaOGae4NmGiQae4NmGiQaemiAaG2aaeWaaeaacuWG4baEgaWcamaaBaaaleaacqaIXaqmaeqaaOGae4NmGiQae4NmGikacaGLOaGaayzkaaGaemiAaG2aaeWaaeaacuWG4baEgaWcamaaBaaaleaacqaIYaGmaeqaaOGae4NmGiQae4NmGikacaGLOaGaayzkaaWaamWaaeaacqWF4oqCdaqadaqaamaaemaabaGafmiEaGNbaSaadaWgaaWcbaGaeGymaedabeaakiab+jdiIkab+jdiIcGaay5bSlaawIa7aiabg6da+maalaaabaGaemyyaegabaGae8xTdugaaaGaayjkaiaawMcaaiab=H7aXnaabmaabaWaaqWaaeaacuWG4baEgaWcamaaBaaaleaacqaIYaGmaeqaaOGae4NmGiQae4NmGikacaGLhWUaayjcSdGaeyOpa4ZaaSaaaeaacqWGHbqyaeaacqWF1oqzaaaacaGLOaGaayzkaaGaeyOeI0Iae8hUde3aaeWaaeaadaabdaqaaiqbdIha4zaalaWaaSbaaSqaaiabigdaXaqabaGccqGFYaIOcqGFYaIOaiaawEa7caGLiWoacqGH+aGpcqWGHbqyaiaawIcacaGLPaaacqWF4oqCdaqadaqaamaaemaabaGafmiEaGNbaSaadaWgaaWcbaGaeGOmaidabeaakiab+jdiIkab+jdiIcGaay5bSlaawIa7aiabg6da+iabdggaHbGaayjkaiaawMcaaaGaay5waiaaw2faaiab=f7aHnaabmaabaWaamWaaeaacuWG4baEgaWcamaaBaaaleaacqaIXaqmaeqaaOGae4NmGiQae4NmGiQaeyOeI0IafmiEaGNbaSaadaWgaaWcbaGaeGOmaidabeaakiab+jdiIkab+jdiIkabgkHiTiqbdohaZzaalaaacaGLBbGaayzxaaaacaGLOaGaayzkaaaaaiaaxMaacaWLjaWaaeWaaeaacqaI1aqncqaI0aanaiaawIcacaGLPaaaaaa@7AFF@

Thus:

g(x→,εs→)=ε2d+p+2qg(x→,s→)+ε2d+p+2q∫d3x→1′′d3x→2′′h(x→1′′)h(x→2′′)[θ(|x→1′′|>aε)θ(|x→2′′|>aε)−θ(|x→1′′|>a)θ(|x→2′′|>a)]α([x→1′′−x→2′′−s→])     (55)
 MathType@MTEF@5@5@+=feaafiart1ev1aaatCvAUfKttLearuWrP9MDH5MBPbIqV92AaeXatLxBI9gBaebbnrfifHhDYfgasaacH8akY=wiFfYdH8Gipec8Eeeu0xXdbba9frFj0=OqFfea0dXdd9vqai=hGuQ8kuc9pgc9s8qqaq=dirpe0xb9q8qiLsFr0=vr0=vr0dc8meaabaqaciaacaGaaeqabaqabeGadaaakeaafaqaaeGabaaabaGaem4zaC2aaeWaaeaacuWG4baEgaWcaiabcYcaSGGaciab=v7aLjqbdohaZzaalaaacaGLOaGaayzkaaGaeyypa0Jae8xTdu2aaWbaaSqabeaacqaIYaGmcqWGKbazcqGHRaWkcqWGWbaCcqGHRaWkcqaIYaGmcqWGXbqCaaGccqWGNbWzdaqadaqaaiqbdIha4zaalaGaeiilaWIafm4CamNbaSaaaiaawIcacaGLPaaaaeaacqGHRaWkcqWF1oqzdaahaaWcbeqaaiabikdaYiabdsgaKjabgUcaRiabdchaWjabgUcaRiabikdaYiabdghaXbaakmaapeaabaGaemizaq2aaWbaaSqabeaacqaIZaWmaaGccuWG4baEgaWcamaaBaaaleaacqaIXaqmaeqaaGGaaOGae4NmGiQae4NmGikaleqabeqdcqGHRiI8aOGaemizaq2aaWbaaSqabeaacqaIZaWmaaGccuWG4baEgaWcamaaBaaaleaacqaIYaGmaeqaaOGae4NmGiQae4NmGiQaemiAaG2aaeWaaeaacuWG4baEgaWcamaaBaaaleaacqaIXaqmaeqaaOGae4NmGiQae4NmGikacaGLOaGaayzkaaGaemiAaG2aaeWaaeaacuWG4baEgaWcamaaBaaaleaacqaIYaGmaeqaaOGae4NmGiQae4NmGikacaGLOaGaayzkaaWaamWaaeaacqWF4oqCdaqadaqaamaaemaabaGafmiEaGNbaSaadaWgaaWcbaGaeGymaedabeaakiab+jdiIkab+jdiIcGaay5bSlaawIa7aiabg6da+maalaaabaGaemyyaegabaGae8xTdugaaaGaayjkaiaawMcaaiab=H7aXnaabmaabaWaaqWaaeaacuWG4baEgaWcamaaBaaaleaacqaIYaGmaeqaaOGae4NmGiQae4NmGikacaGLhWUaayjcSdGaeyOpa4ZaaSaaaeaacqWGHbqyaeaacqWF1oqzaaaacaGLOaGaayzkaaGaeyOeI0Iae8hUde3aaeWaaeaadaabdaqaaiqbdIha4zaalaWaaSbaaSqaaiabigdaXaqabaGccqGFYaIOcqGFYaIOaiaawEa7caGLiWoacqGH+aGpcqWGHbqyaiaawIcacaGLPaaacqWF4oqCdaqadaqaamaaemaabaGafmiEaGNbaSaadaWgaaWcbaGaeGOmaidabeaakiab+jdiIkab+jdiIcGaay5bSlaawIa7aiabg6da+iabdggaHbGaayjkaiaawMcaaaGaay5waiaaw2faaiab=f7aHnaabmaabaWaamWaaeaacuWG4baEgaWcamaaBaaaleaacqaIXaqmaeqaaOGae4NmGiQae4NmGiQaeyOeI0IafmiEaGNbaSaadaWgaaWcbaGaeGOmaidabeaakiab+jdiIkab+jdiIkabgkHiTiqbdohaZzaalaaacaGLBbGaayzxaaaacaGLOaGaayzkaaaaaiaaxMaacaWLjaWaaeWaaeaacqaI1aqncqaI1aqnaiaawIcacaGLPaaaaaa@CC7D@

If |s→
 MathType@MTEF@5@5@+=feaafiart1ev1aaatCvAUfKttLearuWrP9MDH5MBPbIqV92AaeXatLxBI9gBaebbnrfifHhDYfgasaacH8akY=wiFfYdH8Gipec8Eeeu0xXdbba9frFj0=OqFfea0dXdd9vqai=hGuQ8kuc9pgc9s8qqaq=dirpe0xb9q8qiLsFr0=vr0=vr0dc8meaabaqaciaacaGaaeqabaqabeGadaaakeaacuWGZbWCgaWcaaaa@2E2D@| >> *a *then *α*([x→
 MathType@MTEF@5@5@+=feaafiart1ev1aaatCvAUfKttLearuWrP9MDH5MBPbIqV92AaeXatLxBI9gBaebbnrfifHhDYfgasaacH8akY=wiFfYdH8Gipec8Eeeu0xXdbba9frFj0=OqFfea0dXdd9vqai=hGuQ8kuc9pgc9s8qqaq=dirpe0xb9q8qiLsFr0=vr0=vr0dc8meaabaqaciaacaGaaeqabaqabeGadaaakeaacuWG4baEgaWcaaaa@2E37@_1_" - x→
 MathType@MTEF@5@5@+=feaafiart1ev1aaatCvAUfKttLearuWrP9MDH5MBPbIqV92AaeXatLxBI9gBaebbnrfifHhDYfgasaacH8akY=wiFfYdH8Gipec8Eeeu0xXdbba9frFj0=OqFfea0dXdd9vqai=hGuQ8kuc9pgc9s8qqaq=dirpe0xb9q8qiLsFr0=vr0=vr0dc8meaabaqaciaacaGaaeqabaqabeGadaaakeaacuWG4baEgaWcaaaa@2E37@_2_" - s→
 MathType@MTEF@5@5@+=feaafiart1ev1aaatCvAUfKttLearuWrP9MDH5MBPbIqV92AaeXatLxBI9gBaebbnrfifHhDYfgasaacH8akY=wiFfYdH8Gipec8Eeeu0xXdbba9frFj0=OqFfea0dXdd9vqai=hGuQ8kuc9pgc9s8qqaq=dirpe0xb9q8qiLsFr0=vr0=vr0dc8meaabaqaciaacaGaaeqabaqabeGadaaakeaacuWGZbWCgaWcaaaa@2E2D@]) ≈ *α*(s→
 MathType@MTEF@5@5@+=feaafiart1ev1aaatCvAUfKttLearuWrP9MDH5MBPbIqV92AaeXatLxBI9gBaebbnrfifHhDYfgasaacH8akY=wiFfYdH8Gipec8Eeeu0xXdbba9frFj0=OqFfea0dXdd9vqai=hGuQ8kuc9pgc9s8qqaq=dirpe0xb9q8qiLsFr0=vr0=vr0dc8meaabaqaciaacaGaaeqabaqabeGadaaakeaacuWGZbWCgaWcaaaa@2E2D@) since the largest contributions will come from values of |x→
 MathType@MTEF@5@5@+=feaafiart1ev1aaatCvAUfKttLearuWrP9MDH5MBPbIqV92AaeXatLxBI9gBaebbnrfifHhDYfgasaacH8akY=wiFfYdH8Gipec8Eeeu0xXdbba9frFj0=OqFfea0dXdd9vqai=hGuQ8kuc9pgc9s8qqaq=dirpe0xb9q8qiLsFr0=vr0=vr0dc8meaabaqaciaacaGaaeqabaqabeGadaaakeaacuWG4baEgaWcaaaa@2E37@_1_"|, |x→
 MathType@MTEF@5@5@+=feaafiart1ev1aaatCvAUfKttLearuWrP9MDH5MBPbIqV92AaeXatLxBI9gBaebbnrfifHhDYfgasaacH8akY=wiFfYdH8Gipec8Eeeu0xXdbba9frFj0=OqFfea0dXdd9vqai=hGuQ8kuc9pgc9s8qqaq=dirpe0xb9q8qiLsFr0=vr0=vr0dc8meaabaqaciaacaGaaeqabaqabeGadaaakeaacuWG4baEgaWcaaaa@2E37@_2_"| near *a *and so:

g(x→,εs→)=ε2d+p+2qg(x→,s→)+ε2d+p+2qα(s→)∫d3x→1′′d3x→2′′h(x→1′′)h(x→2′′)[θ(|x→1′′|>aε)θ(|x→2′′|>aε)−θ(|x→1′′|>a)θ(|x→2′′|>a)]     (56)
 MathType@MTEF@5@5@+=feaafiart1ev1aaatCvAUfKttLearuWrP9MDH5MBPbIqV92AaeXatLxBI9gBaebbnrfifHhDYfgasaacH8akY=wiFfYdH8Gipec8Eeeu0xXdbba9frFj0=OqFfea0dXdd9vqai=hGuQ8kuc9pgc9s8qqaq=dirpe0xb9q8qiLsFr0=vr0=vr0dc8meaabaqaciaacaGaaeqabaqabeGadaaakeaafaqaaeGabaaabaGaem4zaC2aaeWaaeaacuWG4baEgaWcaiabcYcaSGGaciab=v7aLjqbdohaZzaalaaacaGLOaGaayzkaaGaeyypa0Jae8xTdu2aaWbaaSqabeaacqaIYaGmcqWGKbazcqGHRaWkcqWGWbaCcqGHRaWkcqaIYaGmcqWGXbqCaaGccqWGNbWzdaqadaqaaiqbdIha4zaalaGaeiilaWIafm4CamNbaSaaaiaawIcacaGLPaaaaeaacqGHRaWkcqWF1oqzdaahaaWcbeqaaiabikdaYiabdsgaKjabgUcaRiabdchaWjabgUcaRiabikdaYiabdghaXbaakiab=f7aHnaabmaabaGafm4CamNbaSaaaiaawIcacaGLPaaadaWdbaqaaiabdsgaKnaaCaaaleqabaGaeG4mamdaaOGafmiEaGNbaSaadaWgaaWcbaGaeGymaedabeaaiiaakiab+jdiIkab+jdiIcWcbeqab0Gaey4kIipakiabdsgaKnaaCaaaleqabaGaeG4mamdaaOGafmiEaGNbaSaadaWgaaWcbaGaeGOmaidabeaakiab+jdiIkab+jdiIkabdIgaOnaabmaabaGafmiEaGNbaSaadaWgaaWcbaGaeGymaedabeaakiab+jdiIkab+jdiIcGaayjkaiaawMcaaiabdIgaOnaabmaabaGafmiEaGNbaSaadaWgaaWcbaGaeGOmaidabeaakiab+jdiIkab+jdiIcGaayjkaiaawMcaamaadmaabaGae8hUde3aaeWaaeaadaabdaqaaiqbdIha4zaalaWaaSbaaSqaaiabigdaXaqabaGccqGFYaIOcqGFYaIOaiaawEa7caGLiWoacqGH+aGpdaWcaaqaaiabdggaHbqaaiab=v7aLbaaaiaawIcacaGLPaaacqWF4oqCdaqadaqaamaaemaabaGafmiEaGNbaSaadaWgaaWcbaGaeGOmaidabeaakiab+jdiIkab+jdiIcGaay5bSlaawIa7aiabg6da+maalaaabaGaemyyaegabaGae8xTdugaaaGaayjkaiaawMcaaiabgkHiTiab=H7aXnaabmaabaWaaqWaaeaacuWG4baEgaWcamaaBaaaleaacqaIXaqmaeqaaOGae4NmGiQae4NmGikacaGLhWUaayjcSdGaeyOpa4JaemyyaegacaGLOaGaayzkaaGae8hUde3aaeWaaeaadaabdaqaaiqbdIha4zaalaWaaSbaaSqaaiabikdaYaqabaGccqGFYaIOcqGFYaIOaiaawEa7caGLiWoacqGH+aGpcqWGHbqyaiaawIcacaGLPaaaaiaawUfacaGLDbaaaaGaaCzcaiaaxMaadaqadaqaaiabiwda1iabiAda2aGaayjkaiaawMcaaaaa@BD6F@

It is possible to estimate the last integral as:

[−h0∫aεad|x→1′′|xq+d−1]2={[h0q+d1aq+d(εq+d−1)]2;q+d≠0[h0ln⁡ε]2;q+d=0}
MathType@MTEF@5@5@+=feaafiart1ev1aaatCvAUfKttLearuWrP9MDH5MBPbIqV92AaeXatLxBI9gBaebbnrfifHhDYfgasaacH8akY=wiFfYdH8Gipec8Eeeu0xXdbba9frFj0=OqFfea0dXdd9vqai=hGuQ8kuc9pgc9s8qqaq=dirpe0xb9q8qiLsFr0=vr0=vr0dc8meaabaqaciaacaGaaeqabaqabeGadaaakeaadaWadaqaaiabgkHiTiabdIgaOnaaBaaaleaacqaIWaamaeqaaOWaa8qCaeaacqWGKbazdaabdaqaaiqbdIha4zaalaWaaSbaaSqaaiabigdaXaqabaaccaGccqWFYaIOcqWFYaIOaiaawEa7caGLiWoacqWG4baEdaahaaWcbeqaaiabdghaXjabgUcaRiabdsgaKjabgkHiTiabigdaXaaaaeaadaWcaaqaaGqaciab+fgaHbqaaGGaciab9v7aLbaaaeaacqWGHbqya0Gaey4kIipaaOGaay5waiaaw2faamaaCaaaleqabaGaeGOmaidaaOGaeyypa0ZaaiWabeaafaqabeGabaaabaWaamWaaeaadaWcaaqaaiabdIgaOnaaBaaaleaacqaIWaamaeqaaaGcbaGaemyCaeNaey4kaSIaemizaqgaamaalaaabaGaeGymaedabaGaemyyae2aaWbaaSqabeaacqWGXbqCcqGHRaWkcqWGKbazaaaaaOWaaeWaaeaacqqF1oqzdaahaaWcbeqaaiabdghaXjabgUcaRiabdsgaKbaakiabgkHiTiabigdaXaGaayjkaiaawMcaaaGaay5waiaaw2faamaaCaaaleqabaGaeGOmaidaaOGaei4oaSJaemyCaeNaey4kaSIaemizaqMaeyiyIKRaeGimaadabaWaamWaaeaacqWGObaAdaWgaaWcbaGaeGimaadabeaakiGbcYgaSjabc6gaUjab9v7aLbGaay5waiaaw2faamaaCaaaleqabaGaeGOmaidaaOGaei4oaSJaemyCaeNaey4kaSIaemizaqMaeyypa0JaeGimaadaaaGaay5Eaiaaw2haaaaa@7E97@

if *h*(x→
 MathType@MTEF@5@5@+=feaafiart1ev1aaatCvAUfKttLearuWrP9MDH5MBPbIqV92AaeXatLxBI9gBaebbnrfifHhDYfgasaacH8akY=wiFfYdH8Gipec8Eeeu0xXdbba9frFj0=OqFfea0dXdd9vqai=hGuQ8kuc9pgc9s8qqaq=dirpe0xb9q8qiLsFr0=vr0=vr0dc8meaabaqaciaacaGaaeqabaqabeGadaaakeaacuWG4baEgaWcaaaa@2E37@) ≈ *h*_0_|x→
 MathType@MTEF@5@5@+=feaafiart1ev1aaatCvAUfKttLearuWrP9MDH5MBPbIqV92AaeXatLxBI9gBaebbnrfifHhDYfgasaacH8akY=wiFfYdH8Gipec8Eeeu0xXdbba9frFj0=OqFfea0dXdd9vqai=hGuQ8kuc9pgc9s8qqaq=dirpe0xb9q8qiLsFr0=vr0=vr0dc8meaabaqaciaacaGaaeqabaqabeGadaaakeaacuWG4baEgaWcaaaa@2E37@|^*q *^; |x→
 MathType@MTEF@5@5@+=feaafiart1ev1aaatCvAUfKttLearuWrP9MDH5MBPbIqV92AaeXatLxBI9gBaebbnrfifHhDYfgasaacH8akY=wiFfYdH8Gipec8Eeeu0xXdbba9frFj0=OqFfea0dXdd9vqai=hGuQ8kuc9pgc9s8qqaq=dirpe0xb9q8qiLsFr0=vr0=vr0dc8meaabaqaciaacaGaaeqabaqabeGadaaakeaacuWG4baEgaWcaaaa@2E37@| <*a*. Thus:

g(x→,εs→)≈ε2d+p+2qg(x→,s→)+ε2d+p+2qα(s→)[h0q+d1aq+d(εq+d−1)]2     (57)
 MathType@MTEF@5@5@+=feaafiart1ev1aaatCvAUfKttLearuWrP9MDH5MBPbIqV92AaeXatLxBI9gBaebbnrfifHhDYfgasaacH8akY=wiFfYdH8Gipec8Eeeu0xXdbba9frFj0=OqFfea0dXdd9vqai=hGuQ8kuc9pgc9s8qqaq=dirpe0xb9q8qiLsFr0=vr0=vr0dc8meaabaqaciaacaGaaeqabaqabeGadaaakeaacqWGNbWzcqGGOaakcuWG4baEgaWcaiabcYcaSGGaciab=v7aLjqbdohaZzaalaGaeiykaKIaeyisISRae8xTdu2aaWbaaSqabeaacqaIYaGmcqWGKbazcqGHRaWkcqWGWbaCcqGHRaWkcqaIYaGmcqWGXbqCaaGccqWGNbWzcqGGOaakcuWG4baEgaWcaiabcYcaSiqbdohaZzaalaGaeiykaKIaey4kaSIae8xTdu2aaWbaaSqabeaacqaIYaGmcqWGKbazcqGHRaWkcqWGWbaCcqGHRaWkcqaIYaGmcqWGXbqCaaGccqWFXoqycqGGOaakcuWGZbWCgaWcaiabcMcaPmaadmaabaWaaSaaaeaacqWGObaAdaWgaaWcbaGaeGimaadabeaaaOqaaiabdghaXjabgUcaRiabdsgaKbaadaWcaaqaaiabigdaXaqaaiabdggaHnaaCaaaleqabaGaemyCaeNaey4kaSIaemizaqgaaaaakmaabmaabaGae8xTdu2aaWbaaSqabeaacqWGXbqCcqGHRaWkcqWGKbazaaGccqGHsislcqaIXaqmaiaawIcacaGLPaaaaiaawUfacaGLDbaadaahaaWcbeqaaiabikdaYaaakiaaxMaacaWLjaWaaeWaaeaacqaI1aqncqaI3aWnaiaawIcacaGLPaaaaaa@73F4@

In the case d = 3 and q = -2 (dipole sources) then:

g(x→,εs→)≈εp+2g(x→,s→)+εp+2α(s→)[h0a(ε−1)]2     (58)
 MathType@MTEF@5@5@+=feaafiart1ev1aaatCvAUfKttLearuWrP9MDH5MBPbIqV92AaeXatLxBI9gBaebbnrfifHhDYfgasaacH8akY=wiFfYdH8Gipec8Eeeu0xXdbba9frFj0=OqFfea0dXdd9vqai=hGuQ8kuc9pgc9s8qqaq=dirpe0xb9q8qiLsFr0=vr0=vr0dc8meaabaqaciaacaGaaeqabaqabeGadaaakeaacqWGNbWzcqGGOaakcuWG4baEgaWcaiabcYcaSGGaciab=v7aLjqbdohaZzaalaGaeiykaKIaeyisISRae8xTdu2aaWbaaSqabeaacqWGWbaCcqGHRaWkcqaIYaGmaaGccqWGNbWzcqGGOaakcuWG4baEgaWcaiabcYcaSiqbdohaZzaalaGaeiykaKIaey4kaSIae8xTdu2aaWbaaSqabeaacqWGWbaCcqGHRaWkcqaIYaGmaaGccqWFXoqycqGGOaakcuWGZbWCgaWcaiabcMcaPmaadmaabaWaaSaaaeaacqWGObaAdaWgaaWcbaGaeGimaadabeaaaOqaaiabdggaHbaacqGGOaakcqWF1oqzcqGHsislcqaIXaqmcqGGPaqkaiaawUfacaGLDbaadaahaaWcbeqaaiabikdaYaaakiaaxMaacaWLjaWaaeWaaeaacqaI1aqncqaI4aaoaiaawIcacaGLPaaaaaa@5EB7@

Although this equation is complex, it is clear that if only the first term is considered, then

g(x→,εs→)≈εp+2g(x→,s→)+εp+2α(s→)[h0a(ε−1)]2g(x→,s→)≈|s→|2α(s→)     (59)
 MathType@MTEF@5@5@+=feaafiart1ev1aaatCvAUfKttLearuWrP9MDH5MBPbIqV92AaeXatLxBI9gBaebbnrfifHhDYfgasaacH8akY=wiFfYdH8Gipec8Eeeu0xXdbba9frFj0=OqFfea0dXdd9vqai=hGuQ8kuc9pgc9s8qqaq=dirpe0xb9q8qiLsFr0=vr0=vr0dc8meaabaqaciaacaGaaeqabaqabeGadaaakeaafaqaaeGabaaabaGaem4zaCMaeiikaGIafmiEaGNbaSaacqGGSaaliiGacqWF1oqzcuWGZbWCgaWcaiabcMcaPiabgIKi7kab=v7aLnaaCaaaleqabaGaemiCaaNaey4kaSIaeGOmaidaaOGaem4zaCMaeiikaGIafmiEaGNbaSaacqGGSaalcuWGZbWCgaWcaiabcMcaPiabgUcaRiab=v7aLnaaCaaaleqabaGaemiCaaNaey4kaSIaeGOmaidaaOGae8xSdeMaeiikaGIafm4CamNbaSaacqGGPaqkdaWadaqaamaalaaabaGaemiAaG2aaSbaaSqaaiabicdaWaqabaaakeaacqWGHbqyaaGaeiikaGIae8xTduMaeyOeI0IaeGymaeJaeiykaKcacaGLBbGaayzxaaWaaWbaaSqabeaacqaIYaGmaaaakeaacqWGNbWzcqGGOaakcuWG4baEgaWcaiabcYcaSiqbdohaZzaalaGaeiykaKIaeyisISRaeiiFaWNafm4CamNbaSaacqGG8baFdaahaaWcbeqaaiabikdaYaaakiab=f7aHjabcIcaOiqbdohaZzaalaGaeiykaKcaaiaaxMaacaWLjaWaaeWaaeaacqaI1aqncqaI5aqoaiaawIcacaGLPaaaaaa@71E2@

If only the second term is studied for large values of *ε*, then:

g(x→,εs→)≈εp+2g(x→,s→)+εp+2α(s→)[h0a(ε−1)]2g(x→,s→)≈|s→|4[h0a]2α(s→)     (60)
 MathType@MTEF@5@5@+=feaafiart1ev1aaatCvAUfKttLearuWrP9MDH5MBPbIqV92AaeXatLxBI9gBaebbnrfifHhDYfgasaacH8akY=wiFfYdH8Gipec8Eeeu0xXdbba9frFj0=OqFfea0dXdd9vqai=hGuQ8kuc9pgc9s8qqaq=dirpe0xb9q8qiLsFr0=vr0=vr0dc8meaabaqaciaacaGaaeqabaqabeGadaaakeaafaqaaeGabaaabaGaem4zaCMaeiikaGIafmiEaGNbaSaacqGGSaaliiGacqWF1oqzcuWGZbWCgaWcaiabcMcaPiabgIKi7kab=v7aLnaaCaaaleqabaGaemiCaaNaey4kaSIaeGOmaidaaOGaem4zaCMaeiikaGIafmiEaGNbaSaacqGGSaalcuWGZbWCgaWcaiabcMcaPiabgUcaRiab=v7aLnaaCaaaleqabaGaemiCaaNaey4kaSIaeGOmaidaaOGae8xSdeMaeiikaGIafm4CamNbaSaacqGGPaqkdaWadaqaamaalaaabaGaemiAaG2aaSbaaSqaaiabicdaWaqabaaakeaacqWGHbqyaaGaeiikaGIae8xTduMaeyOeI0IaeGymaeJaeiykaKcacaGLBbGaayzxaaWaaWbaaSqabeaacqaIYaGmaaaakeaacqWGNbWzcqGGOaakcuWG4baEgaWcaiabcYcaSiqbdohaZzaalaGaeiykaKIaeyisISRaeiiFaWNafm4CamNbaSaacqGG8baFdaahaaWcbeqaaiabisda0aaakmaadmaabaWaaSaaaeaaieGacqGFObaAdaWgaaWcbaacbaGae0hmaadabeaaaOqaaiab+fgaHbaaaiaawUfacaGLDbaadaahaaWcbeqaaiabikdaYaaakiab=f7aHjabcIcaOiqbdohaZzaalaGaeiykaKcaaiaaxMaacaWLjaWaaeWaaeaacqaI2aGncqaIWaamaiaawIcacaGLPaaaaaa@78C8@

In either case, the recorded cross covariance function decays slower with |s→
 MathType@MTEF@5@5@+=feaafiart1ev1aaatCvAUfKttLearuWrP9MDH5MBPbIqV92AaeXatLxBI9gBaebbnrfifHhDYfgasaacH8akY=wiFfYdH8Gipec8Eeeu0xXdbba9frFj0=OqFfea0dXdd9vqai=hGuQ8kuc9pgc9s8qqaq=dirpe0xb9q8qiLsFr0=vr0=vr0dc8meaabaqaciaacaGaaeqabaqabeGadaaakeaacuWGZbWCgaWcaaaa@2E2D@| for large distances between the electrodes than the neuronal correlation function *α*(s→
 MathType@MTEF@5@5@+=feaafiart1ev1aaatCvAUfKttLearuWrP9MDH5MBPbIqV92AaeXatLxBI9gBaebbnrfifHhDYfgasaacH8akY=wiFfYdH8Gipec8Eeeu0xXdbba9frFj0=OqFfea0dXdd9vqai=hGuQ8kuc9pgc9s8qqaq=dirpe0xb9q8qiLsFr0=vr0=vr0dc8meaabaqaciaacaGaaeqabaqabeGadaaakeaacuWGZbWCgaWcaaaa@2E2D@). For q = -3 (quadrupole sources):

g(x→,εs→)≈εpg(x→,s→)+εpα(s→)[h0ln⁡ε]2     (61)
 MathType@MTEF@5@5@+=feaafiart1ev1aaatCvAUfKttLearuWrP9MDH5MBPbIqV92AaeXatLxBI9gBaebbnrfifHhDYfgasaacH8akY=wiFfYdH8Gipec8Eeeu0xXdbba9frFj0=OqFfea0dXdd9vqai=hGuQ8kuc9pgc9s8qqaq=dirpe0xb9q8qiLsFr0=vr0=vr0dc8meaabaqaciaacaGaaeqabaqabeGadaaakeaacqWGNbWzcqGGOaakcuWG4baEgaWcaiabcYcaSGGaciab=v7aLjqbdohaZzaalaGaeiykaKIaeyisISRae8xTdu2aaWbaaSqabeaacqWGWbaCaaGccqWGNbWzcqGGOaakcuWG4baEgaWcaiabcYcaSiqbdohaZzaalaGaeiykaKIaey4kaSIae8xTdu2aaWbaaSqabeaacqWGWbaCaaGccqWFXoqycqGGOaakcuWGZbWCgaWcaiabcMcaPiabcUfaBjabdIgaOnaaBaaaleaacqaIWaamaeqaaOGagiiBaWMaeiOBa4Mae8xTduMaeiyxa01aaWbaaSqabeaacqaIYaGmaaGccaWLjaGaaCzcamaabmaabaGaeGOnayJaeGymaedacaGLOaGaayzkaaaaaa@596D@

Since the logarithm of *ε *is a very slowly varying function, the measured cross correlation function scales very nearly as the neuronal correlation function.
